# Chemoenzymatic Diazo Synthesis Enabled by Enzymatic Halide Recycling with Vanadium-Dependent Haloperoxidases

**DOI:** 10.1021/jacs.5c17554

**Published:** 2026-01-05

**Authors:** Manik Sharma, Yue Li, Kirklin L. McWhorter, Tiffany V. Alvarez, Lorenzo Layug, Abbas Khambatti, Katherine M. Davis, Kyle F. Biegasiewicz

**Affiliations:** † Department of Chemistry, 1371 Emory University, Atlanta, Georgia 30322, United States; ‡ School of Molecular Sciences, Arizona State University, Tempe, Arizona 85281, United States

## Abstract

Diazo compounds are privileged carbene precursors in synthetic organic chemistry. Despite their versatility in both chemo- and biocatalytic chemical synthesis, their preparation typically requires the use of reagents that are expensive, toxic, and unsustainable. Herein, we describe a chemoenzymatic strategy for the preparation of stabilized diazo compounds enabled by enzymatic halide recycling with vanadium-dependent haloperoxidase (VHPO) enzymes. The process involves the conversion of a carbonyl-containing compound to an intermediate hydrazone that is subjected directly to a VHPO-catalyzed nitrogen–nitrogen (N–N) bond oxidation to afford the corresponding diazo compound. The protocol is applied to a broad range of carbonyls with moderate to high yield and excellent chemoselectivity. A series of molecular docking, molecular dynamics, microscale thermophoresis, and mutagenesis experiments provide insight into reactivity rate differences between (E)- and (Z)-configured hydrazones in the VHPO-mediated oxidation process. Finally, the developed method is interfaced with lipase-mediated transacylation to produce a collection of diazo derivatives starting from a single benzoylformate starting material.

## Graphical abstract

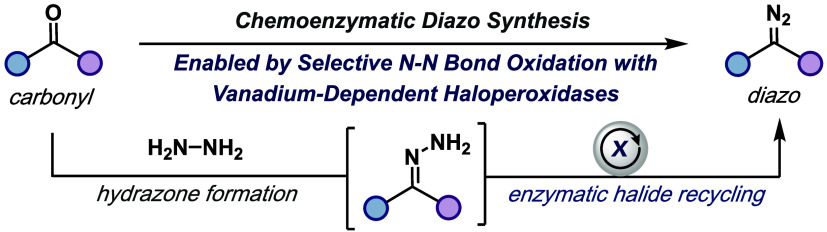

## Introduction

Diazo compounds are a privileged class of carbene-transfer reagents in organic chemistry. Their synthetic versatility has been demonstrated in a broad range of chemo- and biocatalytic transformations including cyclopropanation,^1–3^ cycloaddition,^4,5^ and insertion reactions.^6,7^ Despite the advancements made in the synthetic application of diazo compounds, their high reactivity profiles present significant technical challenges affiliated with their isolation and storage,^8^ necessitating the development of safe and scalable methods for their preparation. Traditional methods include the base-promoted Bamford-Stevens reaction with tosylhydrazones,^9^ Regitz diazo transfer,^10^ and oxidative dehydrogenation of hydrazones using toxic metal reagents that suffer from poor atom economy and/or the generation of undesired byproduct formation.^11–15^ While significant progress has been made toward more sustainable preparation of diazo compounds using milder oxidants,^11,15–20^ electrochemical oxidation,^21^ and aerobic oxidation of hydrazones over metals on nitrogen-doped carbon (M–N-C),^22^ these methods rely on organic solvents that limit their application in chemoenzymatic synthesis (Figure 1a).

**Figure 1:**
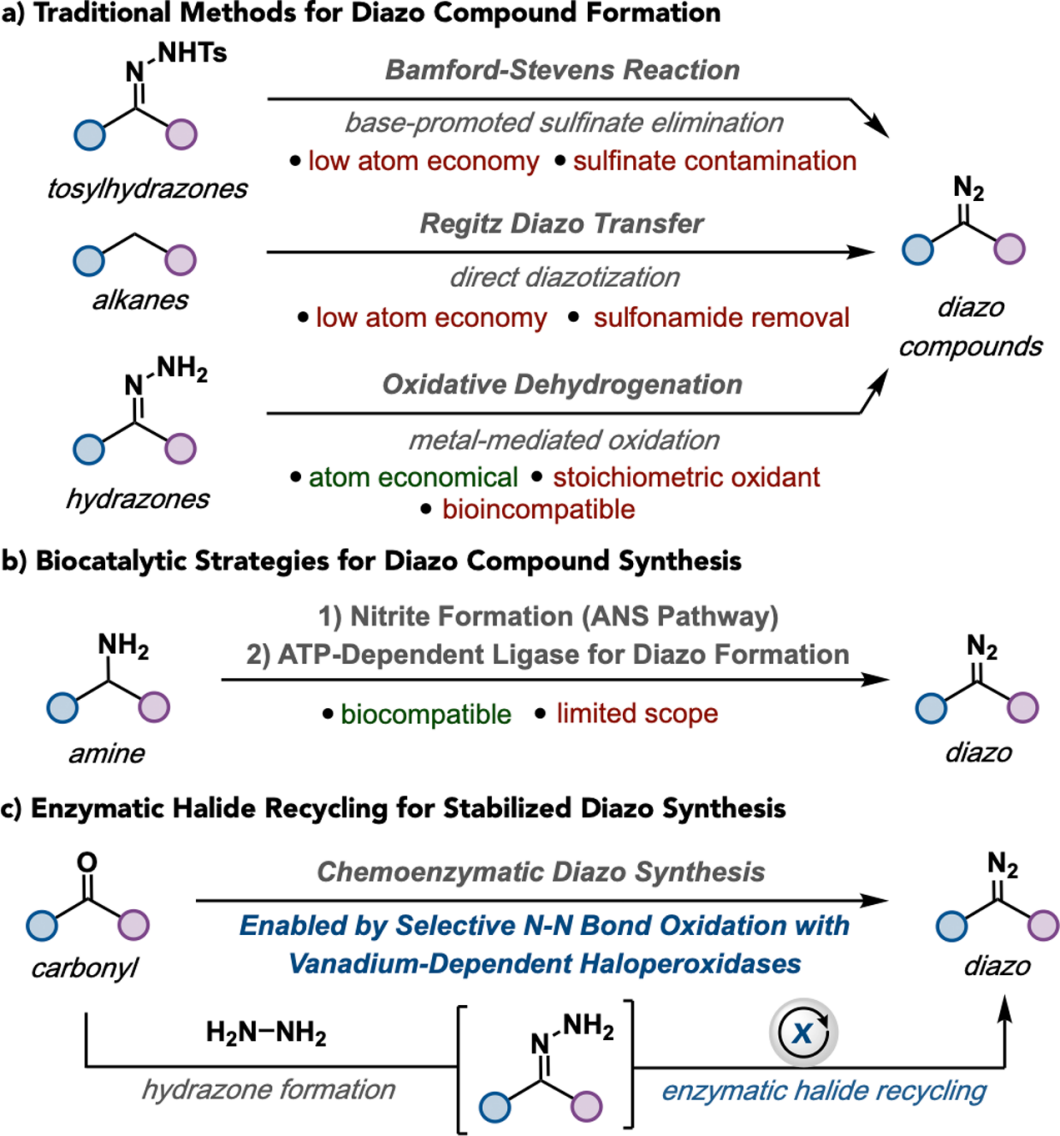
Diazo synthesis overview. (a) Traditional methods for diazo formation. (b) Biocatalytic strategies for diazo compound synthesis. (c) enzymatic halide recycling for diazo compound synthesis.

Enzymes are an attractive alternative for diazo compound synthesis because of their efficiency, selectivity, and sustainability parameters.^23,24^ While a small collection of diazo-forming enzymes have been characterized in the context of natural product biosynthesis,^25–28^ they are all dependent on the production of nitrite by the l-aspartate-nitro-succinate (ANS) pathway, followed by a separate ATP-dependent ligase for diazo formation (Figure 1b),^29^ inherently limiting their practical application in chemoenzymatic synthesis. Alternative modes of diazo formation are known to be employed during the biosynthesis of kinamycin^30^ and azaserine,^31^ however, the enzymes responsible have yet to be identified. Moreover, the suite of characterized diazo forming enzymes only produces a small collection of acceptor-only diazos, leaving enzymatic options to produce other diazo types highly desired. To dramatically expand the synthetic utility of diazos in chemical synthesis and synthetic biology, we were particularly drawn to the establishment of a chemoenzymatic system for the synthesis of donor–acceptor diazos because of their superior performance in a wide range of C–H functionalization reactions.^32–38^ To this end, we hypothesized that haloperoxidase enzymes could be repurposed for halogenation-mediated oxidation of hydrazones. Among the variety of halogenating enzymes in nature,^39–41^ vanadium-dependent haloperoxidase (VHPO) enzymes have been recognized as an attractive biocatalyst platform for chemical synthesis because of their facile recombinant expression in *E. coli*, lack of a required turnover system, resistance to oxidative degradation, stability in organic solvents, and tolerance of high temperatures.^42–45^ VHPOs from marine algae, fungi, and bacteria perform catalytic oxidation of halides using hydrogen peroxide (H_2_O_2_) as the terminal oxidant.^39–45^ In nature, this mechanism is used to perform selective electrophilic halogenation of arenes and enolizable carbon centers,^39–46^ haloetherification,^47–51^ halolactonization,^52^ and polyene cyclizations.^53^ Because of their ease of operation and extensive reaction profile in nature, VHPOs have emerged as a robust biocatalytic system for performing a wide range of new reactions outside of their native reaction scope.^42,54–59^ We recently discovered that VHPOs facilitate the formation of nitrogen-halogen (N-X) bonds in the context of aromatic heterocycle synthesis.^60^ Despite the advancement of this work that enables heteroatom-heteroatom bond formation through a cryptic halogenation event using superstoichiometric quantities of halide,^61^ a mechanism for halogenation-mediated nitrogen activation using a catalytic quantity of halide would be advantageous from an operational, mass intensity, and cost effectiveness standpoint. We hypothesized that hydrazone oxidation could be accomplished using an enzymatic halide recycling (EHR) mechanism, whereby the oxidation event could be performed by repetitive oxidation of a catalytic quantity of halide using H_2_O_2_ as the terminal oxidant.^62–64^ Our proposed chemoenzymatic system would feature hydrazone formation through condensation between hydrazine (NH_2_–NH_2_) and a carbonyl compound followed by VHPO-mediated nitrogen–nitrogen (N–N) bond oxidation using EHR. Herein, we report that VHPOs are effective biocatalysts for hydrazone oxidation.

## Results and Discussion

Studies were initiated by examining the oxidation of ethyl-2-hydrazineylidene-2-phenylacetate (**1**) to produce ethyl 2-diazo-2-phenylacetate (**2**). The chloroperoxidase from *Curvularia inaequalis* (*Ci*VCPO) was initially interrogated based on its established synthetic applicability in a wide range of synthetic transformations.^42,54,56,57,64–70^ Subjection of **1** to *Ci*VCPO (0.025 mol %), sodium orthovanadate (Na_3_VO_4_, 1 mM), potassium bromide (KBr, 1.0 equiv), and H_2_O_2_ (2.0 equiv) in citrate buffer (100 mM, pH = 6) and 2-methyltetrahydrofuran (2-MeTHF) as cosolvent (40% v/v) provided hydrazone **2** in 77% yield in 18 h (Figure 2, entry 1). To identify a more suitable biocatalyst for hydrazone oxidation, a small collection of bromoperoxidases from *Acaryochloris marina* (*Am*VBPO),^71^ *Corallina officinalis* (*Co*VBPO),^72^ and *Corallina pilulifera* (*Cp*VBPO)^73^ were trialed under the same conditions (Figure 2, entries 2–4). Gratifyingly, *Co*VBPO and *Cp*VBPO performed comparably well, producing **2** in 84% yield. The reaction could be run under enzymatic halide recycling conditions with *Cp*VBPO by lowering the KBr loading to 0.3 equiv and increasing the H_2_O_2_ loading to 6.0 equiv, respectively, providing **2** in 90% yield (Figure 2, entry 5). Control experiments were run in sequence to ensure the necessity of all reaction components including enzyme (*Cp*VBPO), Na_3_VO_4_, KBr, and H_2_O_2_ (Figure 2, Entries 6–9). Individual runs were performed on the (Z)- and (E)-configured hydrazones in isolation to provide **2** in 88 and 94% yield, respectively (Figure 2, entries 10–11). Notably, KBr loadings between 0.1 and 1.0 equiv were tolerated, with a marked decrease in yield when increasing the loading above 1.0 equiv (Supporting Figure S1). Increasing the H_2_O_2_ loading past 8.0 equiv led to a notable decrease in yield (Supporting Figure S2). While the reaction performed in moderate yields in select polar aprotic solvents (DMSO, EtOAc, MeCN, 45–67% yield), 2-MeTHF served as the premier solvent for enzymatic hydrazone oxidation (Supporting Figure S3), particularly at 30–50% loading (v/v) (Supporting Figure S4). Importantly, it was observed that the pH and buffer type play critical roles in reaction performance (Supporting Figures S5–S6). Finally, increasing the substrate loading past 2 mM led to a notable decrease in reaction (Supporting Figure S7). To further simplify the protocol for diazo access, the reaction was performed through generation of hydrazone **1** with hydrazine hydrate (NH_2_NH_2_–H_2_O) and acetic acid (AcOH) followed by direct subjection to enzymatic hydrazone oxidation to generate **2** in 87% yield over two steps (Figure 2, entry 12).

**Figure 2:**
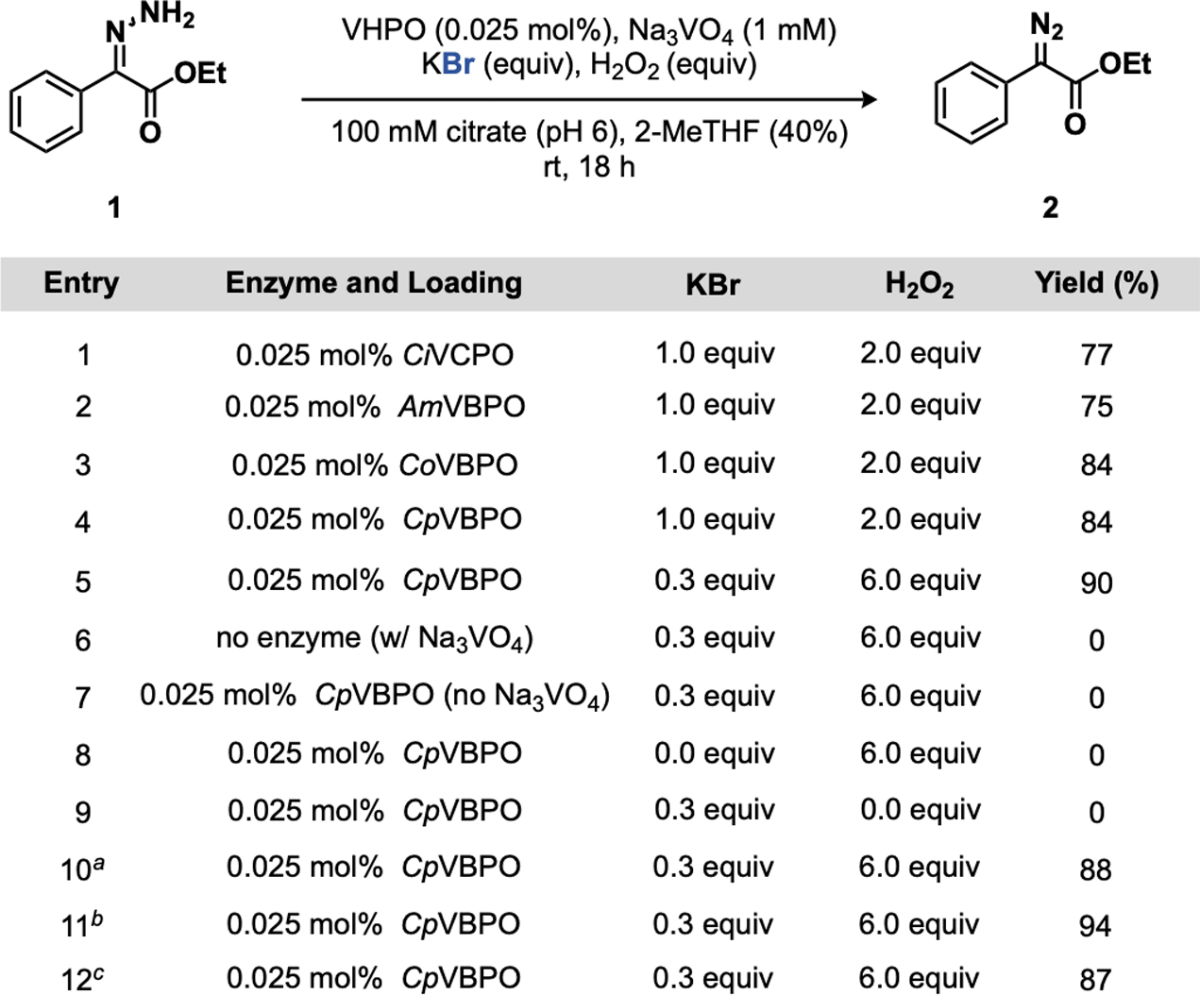
Optimization experiments for biocatalytic oxidation of hydrazones. Reaction conditions: **1** (2.0 μmol, 0.4 mg), VHPO (0.025 mol %), Na_3_VO_4_ (1 mM), KBr (0.3–1.0 equiv), H_2_O_2_ (1.0–6.0 equiv), citrate buffer (100 mM, pH = 6, 200 μL), 2-MeTHF (400 μL), 1 mL total reaction volume, 18 h, rt. Yields determined by HPLC based on a calibration curve. See Supporting Information for details. ^*a*^(*Z*)-configured hydrazone was used. ^*b*^(*E*)-configured hydrazone was used. ^*c*^Performed with crude generation of **1**.

Following reaction optimization, the substrate scope of the developed VHPO-catalyzed hydrazone oxidation was interrogated. The one-pot two-step protocol accommodates methyl-and *tert*-butyl substitution at the *para* position of the starting α-ketoester, furnishing the corresponding diazos in 79% yield and 3160 total turnover number (TTN) and 60% and 2400 TTN, respectively (Figure 3, 3–4). A substrate containing an electron-donating methoxy group in the same position performs in 81% yield and 3240 TTN (Figure 3, 5). Substrates bearing electron withdrawing groups in the *para*-position including chloro-, bromo-, fluoro-, and cyano-substitution are tolerated in a range of 65–74% yield and 2600–2960 TTN (Figure 3, 6–9). Additionally, a methoxy group was tolerated in the *meta*-position of the aryl α-ketoester in 80% yield and 3200 TTN (Figure 3, **10**). Gratifyingly, this reaction protocol could be extended to isatin substrates. *N*-phenyl- and *N*-methyl-substituted isatins perform in 85% yield and 3400 TTN and 64% yield and 2560 TTN, respectively (Figure 3, **11–12**). The reaction accommodates a variety of *N*-benzyl-substituted isatins including unsubstituted, 5- methyl- and methoxy-, and 7-methyl substituted derivatives in 70–84% yield and with 2800–3360 TTN (Figure 3, **13–16**). Gratifyingly, an isatin without *N*-substitution is accommodated in 66% yield and 2640 TTN (Figure 3, **17**). The protocol was also applied to the synthesis of an alkyl-containing diazo in 78% yield and 3120 TTN (Figure 3, **18**). Finally, the method was effective for generating diaryl diazos in 11–22% yield and 440–880 TTN (Figure 3, **19–21**). Notably, low yields of these substrates can be attributed to observed hydrolysis of the hydrazone under the reaction conditions.

**Figure 3:**
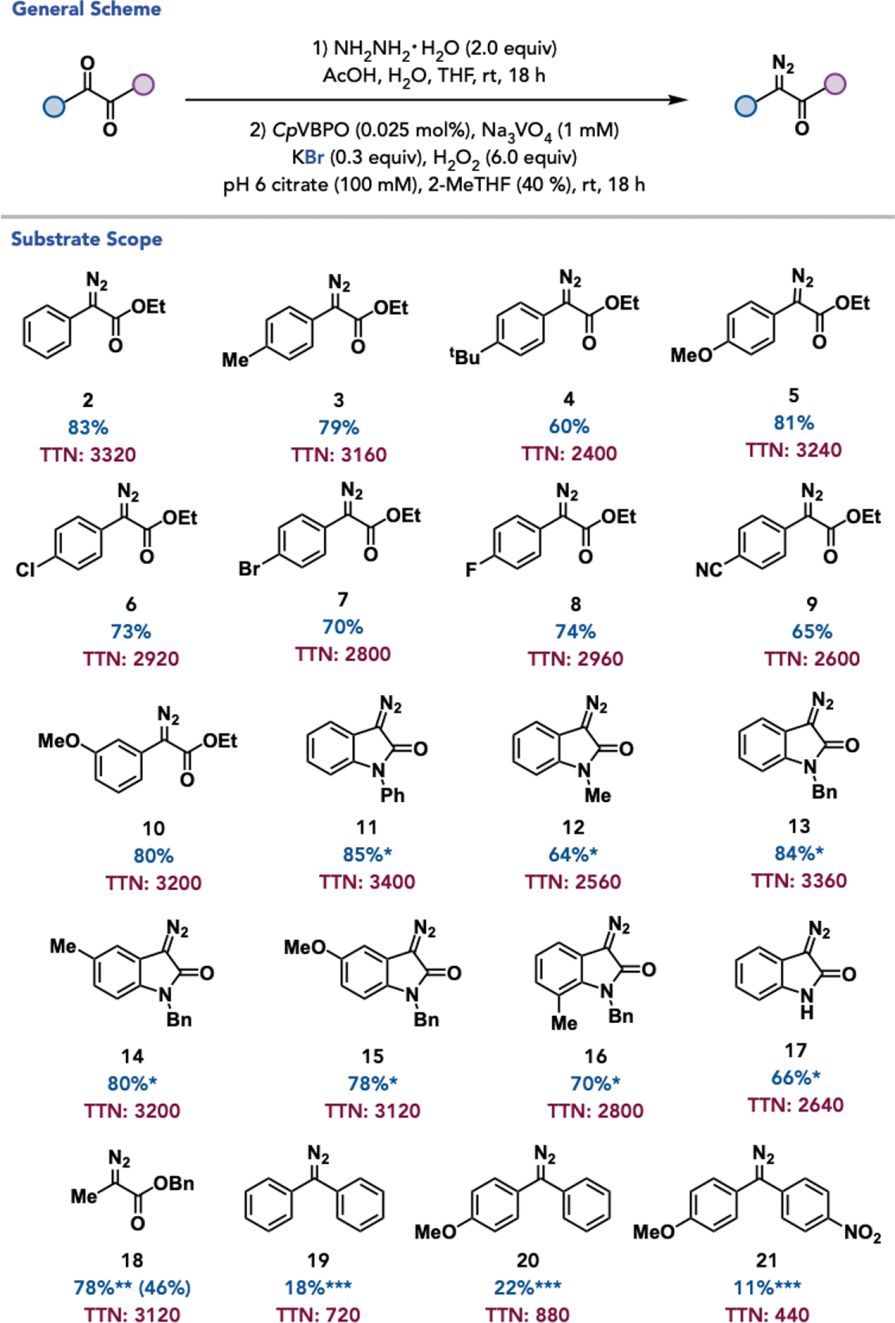
Substrate scope for VHPO-catalyzed hydrazone oxidation. Reaction conditions: (1) substrate (0.4 mmol), NH_2_NH_2_^.^H_2_O (2.0 equiv), AcOH (100 uL), H_2_O (100 μL), THF (100 μL), rt, 18 h; (2) *Cp*VBPO (0.025 mol %), Na_3_VO_4_ (1 mM final concentration), KBr (0.3 equiv), H_2_O_2_ (6.0 equiv), citrate buffer (100 mM, pH = 6), 2-MeTHF (40%), 18 h, rt. used. *(1) substrate (0.4 mmol), NH_2_NH_2_^.^H_2_O (2.0 equiv), AcOH (100 uL), H_2_O (100 μL), THF (300 μL), rt, 18 h; (2) *Cp*VBPO (0.025 mol %), Na_3_VO_4_ (1 mM final concentration), KBr (0.3 equiv), H_2_O_2_ (6.0 equiv), citrate buffer (100 mM, pH = 6), 2-MeTHF (40%), 18 h, rt. used. **analytical yield. ***substrate (0.4 mmol), NH_2_NH_2_^.^H_2_O (10.0 equiv), AcOH (50 uL), EtOH (500 μL), reflux, 18 h; (2) *Cp*VBPO (0.025 mol %), Na_3_VO_4_ (1 mM final concentration), KBr (0.3 equiv), H_2_O_2_ (6.0 equiv), tris buffer (100 mM, pH = 8), 2-MeTHF (40%), 18 h, rt. used. Yields determined by isolation. TTNs were determined by dividing the quantity of the resulting product by the concentration of the enzyme used and assuming quantitative conversion of hydrazone formation. See the Supporting Information for more details.

A proposed mechanism for the established VHPO-mediated oxidation of hydrazones is outlined in Figure 4.^39–42^ VHPOs are dependent on a histidine-coordinated vanadate cofactor (**I**). Upon H_2_O_2_ exposure, two water molecules are displaced to generate the corresponding peroxovanadium intermediate (**II**) that is poised for nucleophilic attack by a halide anion (X^–^), generating a vanadium-bound hypohalite species (**III**) that can participate directly as the halogenating species or is released from the coordination sphere as hypohalous acid for a subsequent halogenation event. We propose that one of these halogenation pathways is responsible for the N-halogenation of the starting amino nitrogen of the hydrazone to generate the corresponding *N*-halohydrazone. An ensuing elimination of the halide would result in the diazo formation and regeneration of halide that is recycled by the enzyme for a new halogenation event driven by H_2_O_2_ as the terminal oxidant.

**Figure 4:**
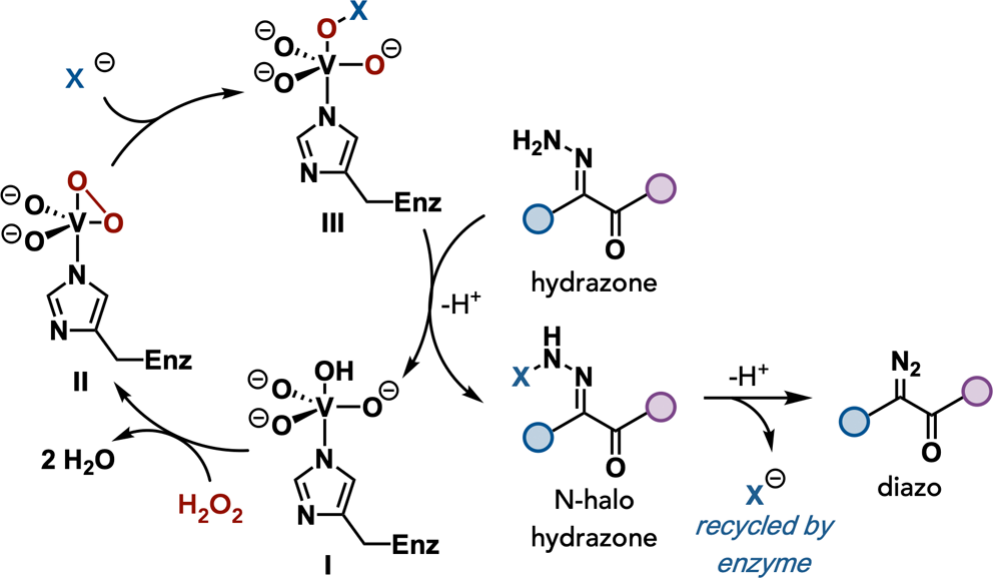
Proposed mechanism for VHPO-mediated oxidation of hydrazones.

Throughout our investigation, we observed a notable reaction performance difference for the more reactive (E)-configured hydrazone (**(E)-1**) and (Z)-configured hydrazone (**(Z)-1**). To gain additional insight into the reaction efficiency of each hydrazone stereoisomer, a time course study was conducted. The production of diazo **2** from hydrazone **(E)-1** reaches its optimized yield of 95% within 6 h, whereas **(Z)-1** requires the full 18 h reaction time to achieve its maximum yield of 88% (Figure 5a). To gain insight into this reaction rate discrepancy, we performed molecular docking with **(E)-1** and **(Z)-1**.

**Figure 5:**
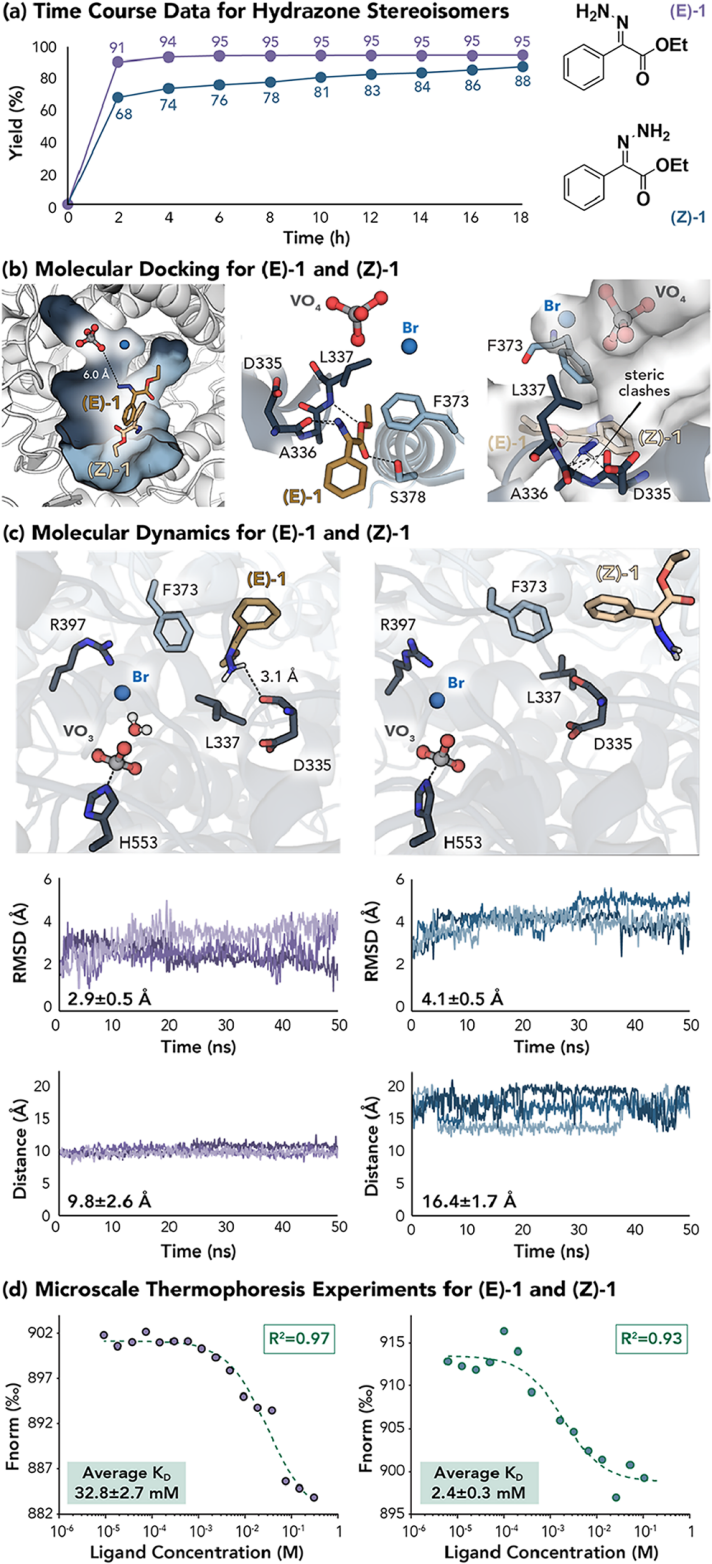
Reactivity and binding studies for (E)- and (Z)-configured hydrazones. (a) Time course data, (b) molecular docking, (c) molecular dynamics simulations, and (d) microscale thermophoresis experiments for **(E)-1** and **(Z)-1**. MD snapshots were taken at 50 ns. RMSDs were calculated for each substrate following alignment of the associated protein backbone to the energy-minimized docking model. Distances were calculated between V and the terminal N of the hydrazones.

More specifically, the substrates were parametrized with SwissParam^74^ and docked to *Cp*VBPO using AutoDock.^75–78^ Previous studies suggest that bromide binding to *Cp*VBPO induces conformational changes essential for forming the hydrophobic substrate-binding pocket at the dimer interface. A recent investigation by Gulder and co-workers has confirmed a similar substrate binding site formation in another VBPO by X-ray crystallography.^55^ Accordingly, we selected a structure of *Cp*VBPO containing the halogen ion (PDB accession code 7QWI) as the docking target and defined the search space along the surface where chains A (dark blue) and B (light blue) interact (Figure 5b, left).^79^ Resultant docking models place the amine nitrogen of the hydrazone moiety of **(E)-1** approximately 6 Å from the vanadate ion (Figure 5b, left). While hydrophobic interactions are expected to facilitate substrate binding, our simulations additionally predict several polar contacts, the majority of which occur with chain A. In particular, both hydrazone nitrogen atoms appear to interact with the backbone carbonyl of D335 via hydrogen bonding and dipole–dipole interactions, respectively (Figure 5b, middle). The ester oxygen, by contrast, forms a hydrogen bond with L337, rearrangement of which helps shape the active site upon bromide-binding. We, therefore, hypothesize that this region (V332-L337) is crucial for substrate recognition and positioning by *Cp*VBPO. An additional H-bonding interaction is observed between the **(E)-1** carbonyl and the side chain of S378 from chain B (Figure 5b, middle).

The top docking pose for **(Z)-1** places the phenyl group in a similar location to that of **(E)-1**. However, the preferred molecular orientation is flipped 180° such that the alkoxycarbonyl group points toward the active site entrance rather than the narrow channel of the main cavity (Figure 5b, left). This positioning significantly extends the distance between the vanadate ion and the amino nitrogen (to ∼14.4 Å), consistent with longer reaction times to reach maximum yield due to reduced reaction efficiency. Alignment of **(Z)-1** to the docking pose for **(E)-1** suggests that steric hindrance of the amino nitrogen necessitates this alternative binding mode, as numerous clashes are observed with the loop preceding L337 (namely, D335 and A336, Figure 5b, right).

To evaluate if steric hindrance could be alleviated through introduction of smaller residues specifically targeting the amidino-ester tail of the substrate, four mutants of *Cp*VBPO were interrogated including A336G, L337G, F373G, and S378A (Supporting Figure S8). A336 directly clashes with **(Z)-1** when forced into the **(E)-1** binding pose, whereas L337, F373, and S378 form the tunnel that accommodates the amidino–ester tail (Figure 5b, middle). We anticipated that these substitutions might permit subtle rearrangements to relieve the predicted clashes with **(Z)-1**, but this was not observed. A336G, F373G, and S378A showed no change in yield or E/Z selectivity, and mutation of L337 instead reduced activity for both isomers, albeit inconsistently. This outcome is somewhat reassuring, as the L337 variant was intended as a negative control given its established role in halide binding, and correspondingly substrate binding, in *Cp*VBPO.^79^ Collectively, these findings suggest that the steric constraints limiting **(Z)-1** binding are imposed by the backbone conformation itself rather than by side-chain bulk, and that even when additional space is introduced, **(Z)-1** either cannot adopt the productive **(E)-1**–like pose, or it preferentially associates with an alternative, less-active site.

The robustness of docking-derived binding modes was subsequently validated via a series of 50 ns molecular dynamics (MD) simulations. Because bond-length constraints could not be applied reliably to orthovanadate, we explored metavanadate, proposed to serve as a reactive metallocofactor in VHPOs, as an alternative model (details in SI).^80–82^ Resultant trajectories confirm that both **(E)-1** and **(Z)-1** maintain binding orientations highly like the previously described docking poses (Figure 5c). The hydrazone nitrogen of **(E)-1** remains pointed toward the vanadate ion, anchored by the aforementioned H-bonding interactions with the D335 backbone carbonyl. While slightly more mobile, **(Z)-1** consistently occupies a distant position with its amino nitrogen largely inaccessible. Interestingly, a more open conformation of the leucine implicated in bromide binding (L337) is also sampled (Supporting Figure S9). These observations are in alignment with the extended reaction time observed experimentally and supports the hypothesis that **(Z)-1** engages the enzyme through an alternative but less catalytically favorable binding mode. Notably, microscale thermophoresis (MST) data suggest that **(Z)-1** may, in fact, bind with higher affinity than **(E)-1** (Figure 5d, Supporting Tables S2–S3), underscoring the distinction between binding strength and productive positioning. We note, however, that MST measurements were limited by challenges in ligand solubility at the high concentrations required to reach a fully bound state. Following solvent screening, DMSO provided the most consistent results, but still did not allow for full binding curve saturation.

Guided by the above computational observations, we attempted to test their validity experimentally via mutagenesis. Although the majority of contacts between **(E)-1** and the enzyme occur with the V332-L337 backbone, our earlier study evaluating the reactivity of *Cp*VBPO with thioamides revealed the importance of D335 for substrate binding.^62^ Whether or not the acidic side chain plays a direct role in anchoring **(E)-1**, we hypothesized that mutation of D335 would likely disrupt the H-bonding network crucial to active site organization and therefore impact substrate binding. Accordingly, we generated the D335G mutant, as reported previously. When the reaction was conducted using model **1** with *Cp*VBPO D335G, complete abolition of activity was accomplished (Figure 6a). To probe the effect of D335G on substrate binding, we again performed 50 ns MD simulations using the same docked pose of **(E)-1** as in the wild-type enzyme. These trajectories placed the substrate slightly farther from the vanadate center (Supporting Figure S10) and more frequently sampled conformations in which the amino group is flipped away from the active site, analogous to **(Z)-1** (Figure 6b, left). Consequently, the interaction with residue 335 is weakened relative to the native simulations (Figures 5c, left and 6b, left).

**Figure 6:**
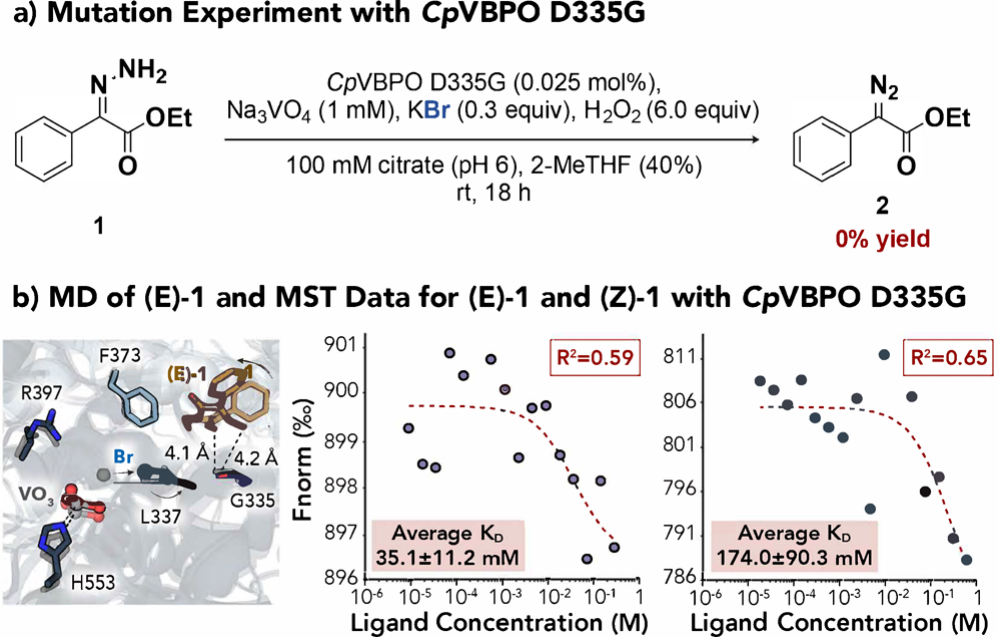
Mutation studies for *Cp*VBPO. (a) Reactivity with *Cp*VBPO D335G. (b) MD simulations for **(E)-1**, as well as MST experiments for **(E)-1** and **(Z)-1** bound to *Cp*VBPO D335G. MD snapshots were taken at 5 (transparent) and 50 ns (solid), respectively.

Bromide was also disrupted in the mutant, with the average RMSDs for the halide both higher and more variable than in the wild-type simulations (Supporting Figures S11–S13). Inspection of a 50 ns snapshot depicts the ion escaping interaction with R397 (Figure 6b, left), recently identified as essential for halide binding.^79^ This contrasts sharply with the native simulations, during which Br^–^ remained stably coordinated to the active site arginine throughout. Thus, the loss of activity in D335G likely reflects a combination of impaired substrate positioning and disruption of the bromide-binding network, which are inherently coupled because halide binding is required for productive substrate association.

MST experiments with the mutant were considerably less reliable than those with the wild-type enzyme, perhaps consistent with decreased affinity for the substrate. Binding curves were poorly defined and trends difficult to extract (Figure 6b middle and right, Supporting Tables S4–S5), likely due to increased *K*_D_ values, which, together with solubility limits, prevented access to a fully bound state. While estimates for dissociation constants should be interpreted with caution, the qualitative agreement between MST, mutagenesis, and MD strengthens the conclusion that disruption of the D335 network compromises both halide and substrate binding.

To assess the applicability of the developed VHPO-catalyzed hydrazone oxidation protocol, reactions were conducted using both lysate and whole cells. To our delight, the reaction performed comparably well with both dry and wet lysate and whole cells with yields ranging from 85 to 87% yield (Figure 7a). The whole cell protocol was subsequently incorporated into our developed protocol to perform the tandem hydrazone formation and VHPO-catalyzed hydrazone oxidation on ethyl benzoylformate (**22**) to access diazo **2** in 78% yield over two steps (Figure 7b). Finally, VHPO-mediated hydrazone oxidation was coupled to a lipase catalyzed transacylation reaction starting from **22**. This three step chemoenzymatic protocol was successful in the preparation of methyl-, propyl, isopropyl- and butyl diazos in 74–79% yield and 2960–3160 TTN (Figure 7c, **23–26**).

**Figure 7:**
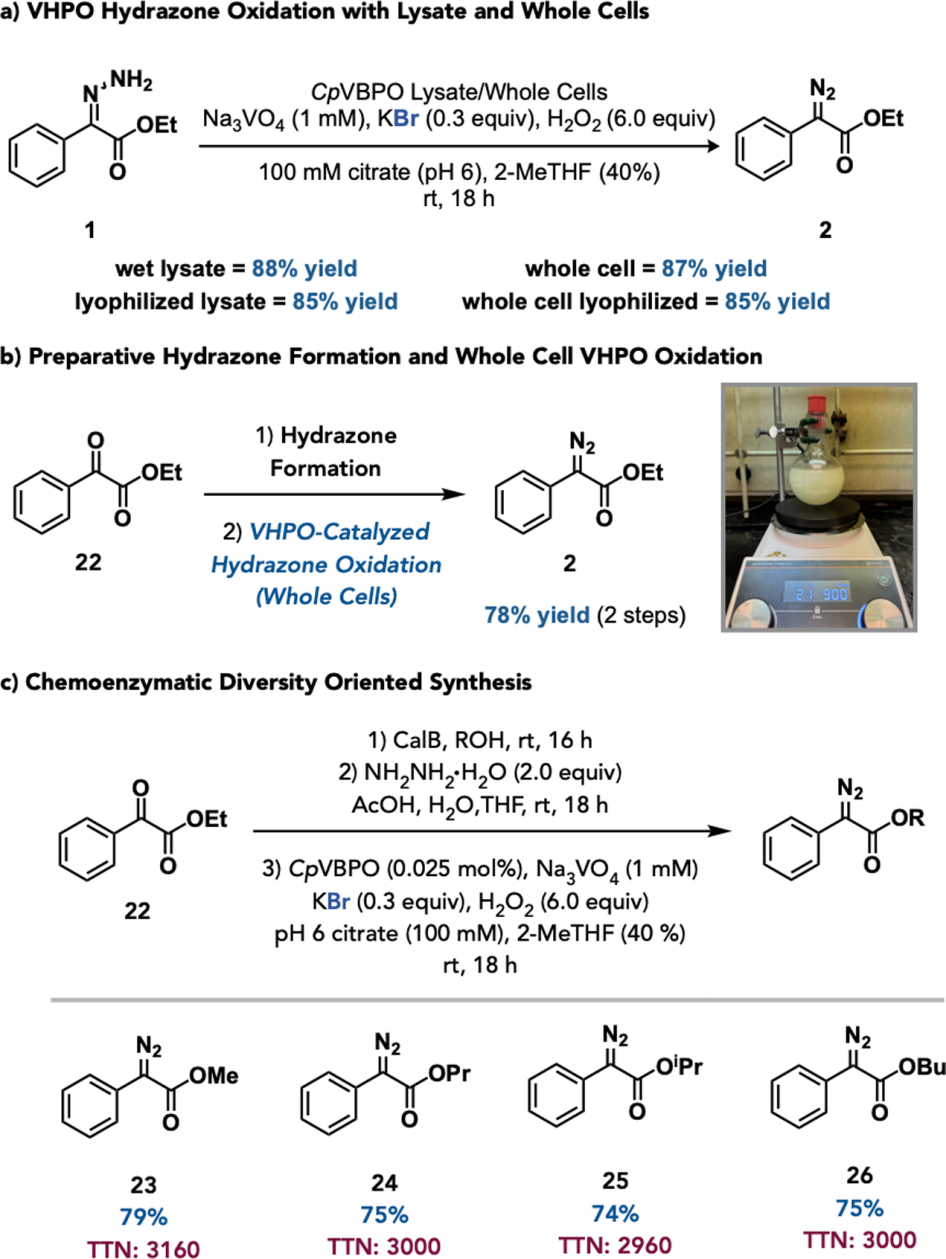
(a–c) Synthetic applicability of VHPO-mediated hydrazone oxidation.

## Conclusion

In summary, a two-step chemoenzymatic protocol has been developed to convert carbonyls to the corresponding diazos. The method is applicable to a wide range of substrates, operates in cell lysate and whole cells, and provides a safe and biocompatible strategy for accessing diazos. This study provides a useful protocol for the preparation of diazo compounds and expands the synthetic application of enzymatic halide recycling by VHPOs.

## Supplementary Material



## Notes

Experimental procedures, DNA and protein sequences, characterization data, NMR spectra, Supporting Figures S1–S13, and Supporting Tables S1–S5 (PDF)
