# Supplementary material for: Chemoenzymatic Diazo Synthesis Enabled by Enzymatic Halide Recycling with Vanadium-Dependent Haloperoxidases
Source: J Am Chem Soc. 2026 Jan 5;148(2):2491–500. doi: 10.1021/jacs.5c17554 (PMC12833808; doi:10.1021/jacs.5c17554)

Supplementary Materials for

Chemoenzymatic Diazo Synthesis Enabled by Enzymatic Halide Recycling with Vanadium-Dependent Haloperoxidases

Manik Sharma[1,2], Yue Li[1+], Kirklin L. McWhorter[1+], Tiffany V. Alvarez[1‡], Lorenzo Layug[1‡], Abbas Khambatti[1], Katherine M. Davis[1*], Kyle F. Biegasiewicz[1,2*]

[1] Department of Chemistry, Emory University, Atlanta, Georgia 30322, USA

[2] School of Molecular Sciences, Arizona State University, Tempe, Arizona 85281, USA

This PDF file includes:

Materials and Methods
Product Characterizations
Supplementary Text
Figs. S1-S13
Spectral Data
References

Table of Contents

General Experimental Information………………………………………………………. 3

Procedures for the Preparation of Lysate and Whole Cells Expressing *Cp*VBPO……. 9

Synthesis and Characterization of Hydrazone Substrates…….…………………….…... 10

Synthesis and Characterization of 1-Benzylindoline-2,3-Dione Substrates………….... 12

General Procedures for VHPO-Catalyzed Diazo Formation…………………...…..... 16

Product Characterization for Diazo Compounds…………………………….……. 19

Additional Reaction Procedures……………………………………………….…..… 39

Optimization Data…………………………………………………………,……... 45

Molecular Docking and Dynamics Experiments….……………………………….……….. 50

Microscale Thermophoresis Experiments………………………………..…...…. 56

References……………………………………………………………………..…,…. 58

Spectroscopic Data………………………………………………………………..…. 62

General Experimental Information

General: Unless specified, all reagents and solvents used in this study were purchased from commercial suppliers and used as received (Combi-Blocks, Sigma-Aldrich, Oakwood Chemicals, Fischer Scientific, VWR). CalB immo Plus™ was purchased from Strem Chemicals (Catalogue No. 07-3130). All nonaqueous reactions were performed using glassware that was flame-dried and capped with a rubber septum under nitrogen atmosphere using an inlet and outlet needle connected to a mineral oil bubbler. All aqueous reactions were conducted using glassware without flame-drying prior to experimental set up and without nitrogen atmosphere. For experiments requiring dried or degassed solvent, it was obtained from a solvent purification system from Pure Process Technology. Unless otherwise indicated, deionized water (H_2O) was used in any experiments where H_2O is included in the procedure.

Chromatography: Flash chromatography was performed on SiliaFlash® P60 (230-400 mesh, particle size 0.040-0.063 mm) using the listed solvent systems in each procedure. Thin-layer chromatography (TLC) was performed using Uniplate HLF 250 micron F254 precoated glass plates and preparative TLC was performed on Uniplate GF 1000 micron F254 precoated glass plates. For TLC analysis, a short-wave UV lamp and/or plate staining was used.

Spectroscopy and HRMS Anaylsis: ^{1}H- and ^{13}C-NMR were obtained on a Bruker AVIII or Bruker NEO (400 and 101 MHz, respectively). Chemical shifts are reported in ppm (δ) downfield from tetramethylsilane and are internally referenced to the internal deuterated solvent indicated. ^{1}H-NMR data is reported as follows: chemical shift [multiplicity, coupling constant (Hz), number of hydrogens]. Multiplicities are reported as follows: s (singlet), b (broad signal), d (doublet), dd (doublet of doublets), ddd (doublet of doublet of doublets), t (triplet), dt (doublet of triplets), tt (triplet of triplets), q (quartet), dq (doublet of quartets), p (pentet), m (multiplet). High-resolution mass spectra were obtained on a Thermo Finnigan LTQ-FTMS spectrometer using APCI or ESI with an orbitrap mass analyzer.

Analytical: Analytical high-performance liquid chromatography (HPLC) was carried out using a Shimadzu LCMS-2020 System with a Kromasil EternityXT-2.5-C18 column (Dimensions: 4.6x50mm, Batch/Serial: 0000016627/A, Part No. XH2CLA05).

Protein Expression and Purification: All protein expression and purification were performed using previously reported methods.[1,2]

<u>Sequence Information for *Cp*VBPO Mutants</u>

<u>Optimized DNA Sequence for *Cp*VBPO A336G Mutant:</u>

ATGGGGATCCCCGCAGACAATTTACAAAGCAGAGCTAAAGCCTCTTTCGACACTCG
GGTCGCAGCAGCCGAGTTGGCACTCAATCGTGGTGTAGTTCCTTCTTTCGCAAATGG
AGAGGAGTTGCTCTACCGGAATCCAGACCCCGACAATACTGACCCATCGTTCATCGC
CAGTTTCACAAAGGGACTTCCTCACGACGACAATGGTGCAATCATAGACCCAGACG
ACTTCCTTGCCTTCGTCCGAGCAATCAATAGTGGGGACGAGAAGGAGATAGCTGAC
CTCACGTTAGGGCCCGCCCGTGACCCTGAGACAGGTCTCCCAATATGGCGTTCTGAC
CTTGCTAATAGTTTAGAGTTAGAGGTTCGTGGGTGGGAGAATAGTTCAGCTGGATTA
ACATTCGACCTTGAGGGGCCAGACGCACAATCTATCGCCATGCCACCTGCTCCAGTT
TTAACTTCTCCCGAGCTCGTTGCCGAGATAGCCGAGCTTTACTTAATGGCCCTCGGTC
GAGAGATAGAGTTCTCTGAGTTCGACTCACCTAAGAATGCCGAGTACATACAATTCG
CAATCGACCAACTTAATGGACTCGAGTGGTTCAATACTCCCGCAAAGCTTGGTGACC
CACCCGCTGAGATACGACGTAGACGTGGTGAGGTAACGGTAGGTAATTTGTTCCGTG
GGATACTCCCAGGGTCTGAGGTCGGTCCTTACTTGTCTCAATACATAATAGTAGGTA
GTAAGCAAATAGGGTCAGCCACTGTAGGGAATAAGACGTTAGTATCGCCAAATGCT
GCCGACGAGTTCGACGGTGAGATCGCTTACGGTTCCATCACTATAAGTCAACGTGTT
CGTATCGCTACTCCAGGTCGTGACTTCATGACTGACCTCAAGGTATTCCTTGACGTTC
AAGACGCTGCCGACTTCCGGGGTTTCGAGTCCTACGAGCCCGGTGCTCGGTTAATCA
GAACGATCAGAGACTTAGCCACATGGGTACACTTCGACGGTTTATACGAGGCTTACT
TAAATGCATGTCTTATCCTTCTCGCCAATGGAGTACCCTTCGACCCAAATTTGCCATT
CCAACAAGAGGACAAGTTAGACAATCAAGACGTTTTCGTAAATTTCGGTTCTGCTCA
CGTCTTGTCTTTAGTAACCGAGGTCGCTACACGTGCTTTGAAGGCTGTAAGATACCA
AAAGTTCAATATCCACAGACGTCTCCGTCCTGAGGCAACGGGTGGGTTAATCTCAGT
TAATAAGATAGCTGCTCAAAAGGGTGAGTCTATCTTCCCAGAGGTTGACCTTGCTGT
TGAGGAGTTGGGTGACATCCTTGAGAAGGCAGAGATCAGTAATCGTAAGCAAAATA
TCGCAGACGGTGACCCAGACCCAGACCCTTCATTCTTATTGCCTATGGCCTTCGCCG
AGGGGTCGCCTTTCCACCCCTCGTACGGGAGTGGGCACGCTGTCGTCGCAGGTGCCT
GTGTAACAATCTTGAAGGCCTTCTTCGACTCTGGAATAGAGATCGACCAAGTATTCG
AGGTAGACAAGGACGAGGACAAGCTCGTCAAGTCCTCATTCAAGGGAACTTTAACT
GTTGCCGGTGAGTTAAATAAGTTGGCAGACAATATCGCTATCGGGCGGAATATGGC
CGGTGTCCACTACTTCTCGGACCAATTCGAGTCTCTTTTGTTAGGGGAGCAAGTAGC
CATAGGTATCTTAGAGGAGCAATCTTTAACATACGGTGAGAATTTCTTCTTCAATTT
GCCAAAGTTCGACGGGACGACTATACAAATCTAA

<u>Amino Acid Sequence *Cp*VBPO A336G Mutant:</u>

MGIPADNLQSRAKASFDTRVAAAELALNRGVVPSFANGEELLYRNPDPDNTDPSFIASFT
KGLPHDDNGAIIDPDDFLAFVRAINSGDEKEIADLTLGPARDPETGLPIWRSDLANSLELE
VRGWENSSAGLTFDLEGPDAQSIAMPPAPVLTSPELVAEIAELYLMALGREIEFSEFDSPK
NAEYIQFAIDQLNGLEWFNTPAKLGDPPAEIRRRRGEVTVGNLFRGILPGSEVGPYLSQYI
IVGSKQIGSATVGNKTLVSPNAADEFDGEIAYGSITISQRVRIATPGRDFMTDLKVFLDVQ
DAADFRGFESYEPGARLIRTIRDLATWVHFDGLYEAYLNACLILLANGVPFDPNLPFQQE
DKLDNQDVFVNFGSAHVLSLVTEVATRALKAVRYQKFNIHRRLRPEATGGLISVNKIAA

QKGESIFPEVDLAVEELGDILEKAEISNRKQNIADGDPDPDPSFLLPMAFAEGSPFHPSYG
SGHAVVAGACVTILKAFFDSGIEIDQVFEVDKDEDKLVKSSFKGTLTVAGELNKLADNI
AIGRNMAGVHYFSDQFESLLLGEQVAIGILEEQSLTYGENFFFNLPKFDGTTIQI

Optimized DNA Sequence for *Cp*VBPO L337G Mutant:

ATGGGTATCCCCGCAGACAATTTGCAATCACGCGCTAAAGCCTCATTCGACACTCGT
GTTGCAGCAGCTGAGCTTGCCTTAAATCGTGGGGTCGTACCTAGTTTCGCTAATGGA
GAGGAGTTACTTTACCGTAATCCAGACCCTGACAATACAGACCCATCATTCATCGCT
TCATTCACGAAGGGGTTGCCACACGACGACAATGGTGCCATAATCGACCCTGACGA
CTTCTTAGCATTCGTACGTGCAATAAATTCCGGTGACGAGAAGGAGATCGCCGACTT
AACTCTCGGGCCCGCTCGTGACCCAGAGACTGGTTTGCCTATCTGGCGTTCCGACTT
GGCTAATTCCCTTGAGTTAGAGGTACGGGGTTGGGAGAATAGTTCGGCCGGATTAAC
GTTCGACCTTGAGGGACCTGACGCACAAAGTATCGCTATGCCACCCGCTCCCGTTCT
TACATCACCCGAGTTGGTAGCAGAGATAGCCGAGCTCTACTTGATGGCCTTAGGACG
TGAGATCGAGTTCAGTGAGTTCGACAGTCCTAAGAATGCAGAGTACATCCAATTCGC
TATCGACCAATTAAATGGGTTGGAGTGGTTCAATACGCCCGCAAAGCTTGGAGACCC
GCCCGCTGAGATCAGAAGACGTCGTGGTGAGGTTACTGTCGGAAATTTGTTCAGAG
GTATCTTACCCGGGTCGGAAGTAGGACCATACTTGAGTCAATACATAATCGTTGGAT
CAAAGCAAATAGGGTCAGCCACAGTCGGAAATAAGACGTTAGTCTCACCTAATGCC
GCAGACGAGTTCGACGGTGAGATAGCATACGGTTCGATAACGATAAGTCAACGTGT
TCGTATAGCAACTCCCGGGCGTGACTTCATGACAGACCTTAAGGTCTTCCTTGACGT
TCAAGACGCTGCCGACTTCCGTGGGTTCGAGTCGTACGAGCCAGGGGCTCGGTTGAT
CCGTACAATCCGAGACTTAGCAACGTGGGTTCACTTCGACGCCGGTTACGAGGCCTA
CTTGAATGCATGTTTAATACTTCTTGCCAATGGAGTACCATTCGACCCTAATCTTCCT
TTCCAACAAGAGGACAAGTTGGACAATCAAGACGTATTCGTTAATTTCGGTTCTGCT
CACGTCTTATCGTTGGTTACAGAGGTTGCCACACGGGCACTCAAGGCCGTACGATAC
CAAAAGTTCAATATCCACAGACGGCTCCGTCCAGAGGCCACAGGTGGTTTAATCTCG
GTTAATAAGATAGCCGCACAAAAGGGAGAGTCTATATTCCCCGAGGTCGACCTTGC
CGTTGAGGAGTTAGGAGACATACTCGAGAAGGCCGAGATATCTAATCGAAAGCAAA
ATATCGCAGACGGTGACCCAGACCCAGACCCCTCATTCCTTTTACCCATGGCATTCG
CAGAGGGATCCCCTTTCCACCCTTCTTACGGAAGTGGTCACGCCGTAGTCGCAGGAG
CCTGTGTTACTATCCTCAAGGCATTCTTCGACTCTGGTATCGAGATCGACCAAGTATT
CGAGGTCGACAAGGACGAGGACAAGCTTGTTAAGTCTAGTTTCAAGGGTACGTTAA
CGGTAGCAGGGGAGCTCAATAAGTTGGCAGACAATATAGCAATCGGTCGGAATATG
GCCGGTGTCCACTACTTCTCTGACCAATTCGAGTCCTTGTTATTAGGTGAGCAAGTTG
CAATAGGAATACTTGAGGAGCAATCTCTCACGTACGGTGAGAATTTCTTCTTCAATT
TGCCTAAGTTCGACGGTACAACGATCCAAATCTAA

Amino Acid Sequence *Cp*VBPO L337G Mutant:

MGIPADNLQSRAKASFDTRVAAAELALNRGVVPSFANGEELLYRNPDPDNTDPSFIASFT
KGLPHDDNGAIIDPDDFLAFVRAINSGDEKEIADLTLGPARDPETGLPIWRSDLANSLELE
VRGWENSSAGLTFDLEGPDAQSIAMPPAPVLTSPELVAEIAELYLMALGREIEFSEFDSPK
NAEYIQFAIDQLNGLEWFNTPAKLGDPPAEIRRRRGEVTVGNLFRGILPGSEVGPYLSQYI

IVGSKQIGSATVGNKTLVSPNAADEFDGEIAYGSITISQRVRIATPGRDFMTDLKVFLDVQ
DAADFRGFESYEPGARLIRTIRDLATWVHFDAGYEAYLNACLILLANGVPFDPNLPFQQ
EDKLDNQDVFVNFGSAHVLSLVTEVATRALKAVRYQKFNIHRRLRPEATGGLISVNKIA
AQKGESIFPEVDLAVEELGDILEKAEISNRKQNIADGDPDPDPSFLLPMAFAEGSPFHPSY
GSGHAVVAGACVTILKAFFDSGIEIDQVFEVDKDEDKLVKSSFKGTLTVAGELNKLADN
IAIGRNMAGVHYFSDQFESLLLGEQVAIGILEEQSLTYGENFFFNLPKFDGTTIQI

<u>Optimized DNA Sequence for *Cp*VBPO F373G Mutant:</u>

ATGGGGATCCCCGCCGACAATCTCCAATCTCGGGCTAAAGCTTCATTCGACACGAGA
GTTGCAGCTGCAGAGTTAGCTTTGAATCGTGGTGTCGTACCTTCATTCGCAAATGGA
GAGGAGCTTTTGTACCGTAATCCTGACCCAGACAATACTGACCCTAGTTTCATCGCT
TCTTTCACTAAGGGGCTCCCACACGACGACAATGGGGCAATCATCGACCCAGACGA
CTTCCTTGCATTCGTCAGAGCTATAAATAGTGGTGACGAGAAGGAGATAGCAGACCT
TACTTTAGGACCAGCTCGTGACCCCGAGACTGGTTTGCCTATATGGCGTTCAGACCT
CGCCAATTCGTTGGAGCTTGAGGTCCGAGGTTGGGAGAATTCGTCAGCAGGTTTGAC
GTTCGACTTGGAAGGGCCCGACGCTCAATCAATCGCCATGCCCCCTGCACCTGTCTT
GACATCTCCAGAGTTAGTTGCCGAGATAGCTGAGTTGTACCTCATGGCTTTGGGGAG
AGAGATCGAGTTCAGTGAGTTCGACTCGCCTAAGAATGCCGAGTACATCCAATTCGC
AATCGACCAACTTAATGGTCTCGAGTGGTTCAATACGCCAGCTAAGCTCGGTGACCC
GCCCGCCGAGATAAGACGACGTCGCGGCGAGGTAACAGTCGGTAATTTATTCCGTG
GTATACTTCCCGGTTCAGAGGTCGGACCTTACCTTTCACAATACATAATCGTAGGTT
CCAAGCAAATCGGTTCGGCAACGGTAGGTAATAAGACACTTGTAAGTCCTAATGCT
GCAGACGAGTTCGACGGTGAGATCGCATACGGTAGTATCACTATCTCTCAACGGGTA
CGTATCGCAACGCCCGGTCGGGACTTCATGACGGACTTGAAGGTTTTCCTCGACGTT
CAAGACGCAGCCGACTTCCGTGGATTCGAGTCTTACGAGCCTGGTGCCCGTCTTATA
CGTACGATAAGAGACTTGGCCACATGGGTTCACTTCGACGCACTTTACGAGGCTTAC
CTTAATGCCTGTTTAATCTTGTTGGCTAATGGAGTACCCTTCGACCCTAATCTCCCAT
TCCAACAAGAGGACAAGCTTGACAATCAAGACGTTGGAGTCAATTTCGGAAGTGCT
CACGTTCTCTCGCTTGTCACAGAGGTAGCCACACGAGCACTCAAGGCCGTAAGATAC
CAAAAGTTCAATATCCACCGTCGTTTGAGACCTGAGGCAACTGGTGGACTCATCTCC
GTTAATAAGATAGCCGCCCAAAAGGGAGAGAGTATATTCCCTGAGGTCGACTTGGC
AGTAGAGGAGTTAGGTGACATCCTCGAGAAGGCAGAGATCTCGAATCGTAAGCAAA
ATATCGCTGACGGTGACCCAGACCCAGACCCATCCTTCCTCTTACCTATGGCTTTCG
CAGAGGGTAGTCCATTCCACCCTAGTTACGGATCTGGTCACGCCGTTGTAGCCGGTG
CATGTGTTACTATATTAAAGGCATTCTTCGACAGTGGTATCGAGATCGACCAAGTCT
TCGAGGTTGACAAGGACGAGGACAAGTTAGTTAAGTCGTCCTTCAAGGGTACATTG
ACGGTAGCAGGGGAGTTAAATAAGTTAGCTGACAATATCGCCATCGGTCGGAATAT
GGCCGGAGTCCACTACTTCTCCGACCAATTCGAGTCACTTTTATTGGGAGAGCAAGT
AGCTATCGGTATCTTAGAGGAGCAATCTTTAACTTACGGAGAGAATTTCTTCTTCAA
TTTACCTAAGTTCGACGGTACGACAATACAAATATAA

<u>Amino Acid Sequence *Cp*VBPO F373G Mutant:</u>

MGIPADNLQSRAKASFDTRVAAAELALNRGVVPSFANGEELLYRNPDPDNTDPSFIASFT
KGLPHDDNGAIIDPDDFLAFVRAINSGDEKEIADLTLGPARDPETGLPIWRSDLANSLELE

VRGWENSSAGLTFDLEGPDAQSIAMPPAPVLTSPELVAEIAELYLMALGREIEFSEFDSPK
NAEYIQFAIDQLNGLEWFNTPAKLGDPPAEIRRRRGEVTVGNLFRGILPGSEVGPYLSQYI
IVGSKQIGSATVGNKTLVSPNAADEFDGEIAYGSITISQRVRIATPGRDFMTDLKVFLDVQ
DAADFRGFESYEPGARLIRTIRDLATWVHFDALYEAYLNACLILLANGVPFDPNLPFQQE
DKLDNQDVGVNFGSAHVLSLVTEVATRALKAVRYQKFNIHRRLRPEATGGLISVNKIAA
QKGESIFPEVDLAVEELGDILEKAEISNRKQNIADGDPDPDPSFLLPMAFAEGSPFHPSYG
SGHAVVAGACVTILKAFFDSGIEIDQVFEVDKDEDKLVKSSFKGTLTVAGELNKLADNI
AIGRNMAGVHYFSDQFESLLLGEQVAIGILEEQSLTYGENFFFNLPKFDGTTIQI

<u>Optimized DNA Sequence for *Cp*VBPO S378A Mutant:</u>

ATGGGTATACCTGCTGACAATTTACAATCGCGTGCTAAGGCCAGTTTCGACACACGG
GTCGCTGCAGCTGAGTTAGCCTTAAATAGAGGTGTTGTTCCATCTTTCGCAAATGGA
GAGGAGTTGTTATACCGTAATCCTGACCCAGACAATACAGACCCATCCTTCATCGCA
TCTTTCACGAAGGGTTTACCTCACGACGACAATGGTGCAATCATAGACCCCGACGAC
TTCTTGGCCTTCGTCAGAGCAATCAATAGTGGAGACGAGAAGGAGATCGCAGACCT
TACTTTGGGGCCTGCACGAGACCCTGAGACGGGTTTGCCAATATGGAGATCAGACCT
TGCCAATTCTTTGGAGTTAGAGGTCCGTGGGTGGGAGAATTCGTCCGCAGGTTTGAC
ATTCGACCTCGAGGGTCCTGACGCTCAAAGTATAGCTATGCCACCTGCACCAGTCCT
CACTAGTCCCGAGCTCGTAGCCGAGATCGCCGAGCTTTACCTCATGGCTCTCGGACG
AGAGATCGAGTTCTCAGAGTTCGACTCCCCCAAGAATGCCGAGTACATCCAATTCGC
TATCGACCAACTTAATGGGCTTGAGTGGTTCAATACACCCGCCAAGTTAGGTGATCC
CCCTGCTGAGATACGTCGACGTCGAGGTGAGGTTACTGTTGGTAATCTTTTCCGAGG
AATCCTTCCAGGGTCTGAGGTTGGTCCTTACTTATCACAATACATCATCGTTGGAAG
TAAGCAAATCGGGAGTGCAACTGTTGGTAATAAGACTTTGGTCTCGCCTAATGCAGC
CGACGAGTTCGACGGAGAGATCGCATACGGGTCTATCACAATCTCGCAACGTGTTCG
GATCGCAACGCCTGGGCGTGACTTCATGACAGACTTGAAGGTCTTCTTGGACGTTCA
AGACGCTGCTGACTTCCGAGGATTCGAGTCCTACGAGCCTGGGGCCAGATTAATCCG
TACTATACGTGACTTAGCCACATGGGTACACTTCGACGCTCTTTACGAGGCATACCT
CAATGCTTGTCTCATACTTCTCGCCAATGGGGTACCTTTCGACCCTAATTTGCCTTTC
CAACAAGAGGACAAGCTTGACAATCAAGACGTTTTCGTTAATTTCGGGGCTGCACAC
GTACTCAGTTTGGTTACAGAGGTTGCAACGCGAGCCCTCAAGGCTGTTCGGTACCAA
AAGTTCAATATCCACCGGAGATTGCGGCCTGAGGCAACGGGTGGTTTGATCTCTGTA
AATAAGATAGCTGCCCAAAAGGGAGAGTCTATCTTCCCTGAGGTTGACTTGGCTGTC
GAGGAGCTCGGGGACATACTCGAGAAGGCTGAGATATCGAATCGGAAGCAAAATAT
CGCCGACGGTGACCCCGACCCCGACCCTAGTTTTCTCCTTCCAATGGCATTCGCCGA
GGGATCGCCCTTCCACCCCAGTTACGGTAGTGGACACGCAGTTGTAGCTGGAGCCTG
TGTTACTATATTAAAGGCATTCTTCGACTCAGGGATAGAGATCGACCAAGTTTTCGA
GGTTGACAAGGACGAGGACAAGCTCGTAAAGTCCTCATTCAAGGGTACTCTCACGG
TTGCTGGTGAGTTAAATAAGTTGGCCGACAATATCGCCATCGGGCGTAATATGGCAG
GGGTTCACTACTTCTCTGACCAATTCGAGTCGCTTCTTCTCGGGGAGCAAGTAGCAA
TCGGTATCCTTGAGGAGCAATCTTTGACTTACGGTGAGAATTTCTTCTTCAATTTGCC
AAAGTTCGACGGTACGACTATCCAAATATAA

Amino Acid Sequence *Cp*VBPO S378A Mutant:

MGIPADNLQSRAKASFDTRVAAAELALNRGVVPSFANGEELLYRNPDPDNTDPSFIASFT
KGLPHDDNGAIIDPDDFLAFVRAINSGDEKEIADLTLGPARDPETGLPIWRSDLANSLELE
VRGWENSSAGLTFDLEGPDAQSIAMPPAPVLTSPELVAEIAELYLMALGREIEFSEFDSPK
NAEYIQFAIDQLNGLEWFNTPAKLGDPPAEIRRRRGEVTVGNLFRGILPGSEVGPYLSQYI
IVGSKQIGSATVGNKTLVSPNAADEFDGEIAYGSITISQRVRIATPGRDFMTDLKVFLDVQ
DAADFRGFESYEPGARLIRTIRDLATWVHFDALYEAYLNACLILLANGVPFDPNLPFQQE
DKLDNQDVFVNFGAAHVLSLVTEVATRALKAVRYQKFNIHRRLRPEATGGLISVNKIAA
QKGESIFPEVDLAVEELGDILEKAEISNRKQNIADGDPDPDPSFLLPMAFAEGSPFHPSYG
SGHAVVAGACVTILKAFFDSGIEIDQVFEVDKDEDKLVKSSFKGTLTVAGELNKLADNI
AIGRNMAGVHYFSDQFESLLLGEQVAIGILEEQSLTYGENFFFNLPKFDGTTIQI

Procedures for the Preparation of Lysate and Whole Cells Expressing *Cp*VBPO

Procedure for the Preparation of Wet Lysate Expressing *Cp*VBPO: *E. coli* cells expressing *Cp*VBPO were pelleted using centrifugation at 3.5 krpm for 20 minutes at 10 °C in a Sorvall ST Plus centrifuge, resuspended in buffer (25 mM pH 6.5 PIPES/H_2SO_4 buffer, 2.5 mM $Ca(NO_3)_2$, 25 mM Na_2SO_4) adjusted to an of OD_{600} = 18.5. Cells were lysed using Qsonica Q500 sonicator with a ½ inch probe on ice in 15 s pulses at 32% amplitude, 30 W in 10 bursts with 55 s between bursts. The lysed cell solution was clarified using centrifugation at 13.0 krpm for 20 minutes at 10 °C to remove cell debris and subsequently transferred into a separate 50 mL conical tube. The clarified cell lysate solution was then aliquoted into 2 mL microcentrifuge tubes, which were then flash frozen using liquid nitrogen and stored at -80 °C until further use.

Procedure for the Preparation of Lyophilized Cell Lysate Expressing *Cp*VBPO: *E. coli* cells expressing *Cp*VBPO were pelleted using centrifugation at 3.5 krpm for 20 minutes at 10 °C in a Sorvall ST Plus centrifuge, resuspended in buffer (25 mM pH 6.5 PIPES/H_2SO_4 buffer, 2.5 mM $Ca(NO_3)_2$, 25 mM Na_2SO_4) adjusted to the OD_{600} = 18.5. Cells were lysed using Qsonica Q500 sonicator with a ½ inch probe on ice in 15 s pulses at 32% amplitude, 30 W in 10 bursts with 55 s between bursts. The lysed cell solution was clarified using centrifugation at 13.0 krpm for 20 minutes at 10 °C to remove cell debris and subsequently transferred into a separate 50 mL conical tube. The clarified cell lysate solution was then flash frozen and lyophilized to yield dried cell lysate that was stored in 4 °C until further use.

Procedure for the Preparation of Whole Cells Expressing *Cp*VBPO: *E. coli* cells expressing *Cp*VBPO were pelleted using centrifugation at 3.5 krpm for 20 minutes at 10 °C in a Sorvall ST Plus centrifuge, resuspended in buffer (25 mM pH 6.5 PIPES/H_2SO_4 buffer, 2.5 mM $Ca(NO_3)_2$, 25 mM Na_2SO_4) adjusted to the OD_{600} = 18.5. The whole cell solution was then aliquoted into 2 mL microcentrifuge tubes and were then flash frozen using liquid nitrogen and stored at -80 °C until further use.

Procedure for the Preparation of Lyophilized Whole Cells Expressing *Cp*VBPO: *E. coli* cells expressing *Cp*VBPO were pelleted using centrifugation at 3.5 krpm for 20 minutes at 10 °C in a Sorvall ST Plus centrifuge, resuspended in buffer (25 mM pH 6.5 PIPES/H_2SO_4 buffer, 2.5 mM $Ca(NO_3)_2$, 25 mM Na_2SO_4) adjusted to the OD_{600} = 18.5. The whole cell solution was then flash frozen and lyophilized to yield dried cell lysate and was stored at 4 °C until further use.

Synthesis and Characterization of Hydrazone Substrates

Ethyl-2-hydrazineylidene-2-phenylacetate (1)

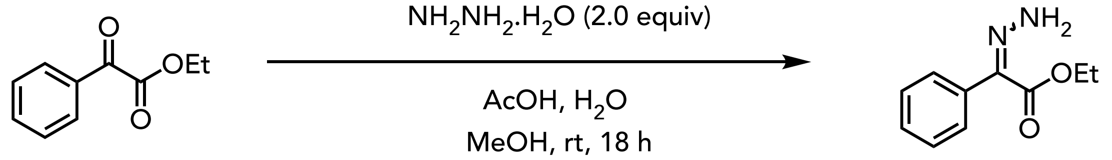

The following procedure was adapted based on a reported literature procedure.[3] A solution of hydrazine hydrate (50-60% in water, 0.62 mL, 10.0 mmol, 2.0 equiv) was slowly added to a 20-dram vial containing a mixture of glacial acetic acid (0.4 mL) and water (0.4 mL) cooled to 0° C in an ice bath. The ethyl 2-oxo-2-phenylacetate (5.00 mmol, 1.0 equiv) was added to the mixture at room temperature and MeOH (0.3 mL) was added to homogenize the solution. The reaction was allowed to stir at room temperature for 18 hr. Upon the completion of reaction, the mixture was quenched by adding saturated $NaHCO_3$ (100 mL) and the MeOH was evaporated under reduced pressure. The resulting residue was extracted with ethyl acetate (3 x 30 mL). The combined organic layers were then washed with brine (70 mL), dried over sodium sulfate (Na_2SO_4), and concentrated under reduced pressure. The resulting crude sample was purified via flash column chromatography to obtain the pure mixture of Z- and E-isomer (**1**).

Ethyl (E)- and (Z)-2-hydrazineylidene-2-phenylacetate [(E)-1) & (Z)-1]

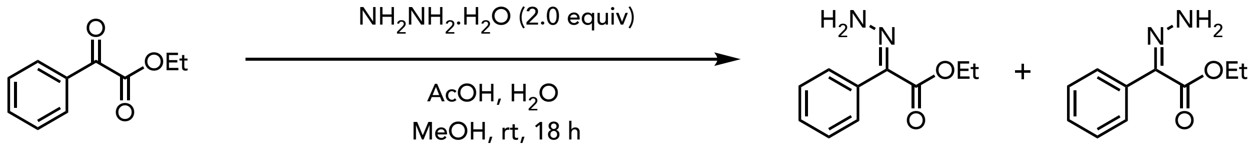

The following procedure was adapted based on a reported literature procedure.[3] A solution of hydrazine hydrate (50-60% in water, 0.62 mL, 10.0 mmol, 2.0 equiv) was slowly added to a 20-dram vial containing a mixture of glacial acetic acid (0.4 mL) and water (0.4 mL) cooled to 0° C in an ice bath. The ethyl 2-oxo-2-phenylacetate (5.00 mmol, 1.0 equiv) was added to the mixture at room temperature and MeOH (0.3 mL) was added to homogenize the solution. The reaction was allowed to stir at room temperature for 18 hr. Upon the completion of reaction, the mixture was quenched by adding saturated $NaHCO_3$ (100 mL) and the MeOH was evaporated under reduced pressure. The resulting residue was extracted with ethyl acetate (3 x 30 mL). The combined organic layers were then washed with brine (70 mL), dried over sodium sulfate (Na_2SO_4), and concentrated under reduced pressure. The resulting crude sample was purified via flash column chromatography to obtain the pure *Z*- (**(E)-1**) and *E*-isomer (**(Z)-1**).

Ethyl (E)-2-hydrazineylidene-2-phenylacetate ((E)-1)

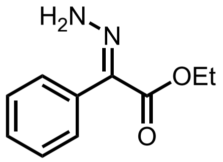

Yield: 25%

Purification: Eluted with 30% EtOAc in Hexanes (R_f = 0.59)

^{1}H NMR (400 MHz, $CDCl_3$) δ 7.51 – 7.45 (m, 2H), 7.44 – 7.38 (m, 1H), 7.34 – 7.27 (m, 2H), 6.21 (s, 2H), 4.30 (q, *J* = 7.1 Hz, 2H), 1.33 (t, *J* = 7.1 Hz, 3H).

^{13}C NMR (101 MHz, $CDCl_3$) δ 164.55, 137.65, 129.65, 129.46, 129.28, 128.93, 61.42, 14.46.

HRMS (ESI) m/z: calculated for $C_{10}H_{13}N_2O_2$ $[M+H]^+$: 193.0972. Found: 193.0972.

Ethyl (Z)-2-hydrazineylidene-2-phenylacetate ((Z)-1)

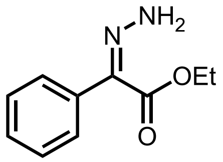

Yield: 75%

Purification: Eluted with 50% EtOAc in Hexanes (R_f = 0.37)

^{1}H NMR (400 MHz, $CDCl_3$) δ 8.43 (s, 2H), 7.57 – 7.46 (m, 2H), 7.38 – 7.27 (m, 3H), 4.31 (q, *J* = 7.1 Hz, 2H), 1.33 (t, *J* = 7.1 Hz, 3H).

^{13}C NMR (101 MHz, $CDCl_3$) δ 163.04, 136.79, 131.21, 128.29, 127.98, 127.62, 60.80, 14.30.

HRMS (ESI) m/z: calculated for $C_{10}H_{13}N_2O_2$ $[M+H]^+$: 193.0972. Found: 193.0972.

Synthesis and Characterization of 1-Benzylindoline-2,3-Dione Substrates

General Procedure for Preparation of 1-Benzylindoline-2,3-dione Substrates (General Procedure A):

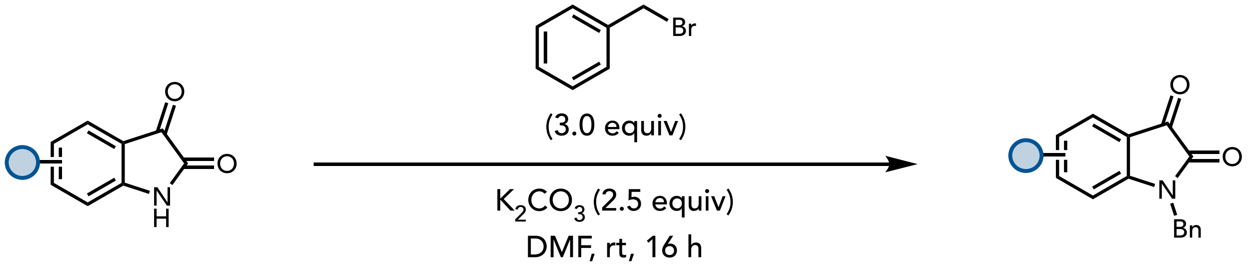

The following procedure was adapted based on a reported literature procedure.[4] Benzyl bromide (3.0 equiv) was added to a stirred solution of corresponding indoline-2,3-dione (6.00 mmol, 1.0 equiv) and K_2CO_3 (15.0 mmol, 2.5 equiv) in DMF (10 mL) and stirred at room temperature for 16 h. After the indicated time, the reaction mixture was quenched with water and extracted with dichloromethane (3 x 50 mL). Combined organic extracts were washed with brine (50 mL), dried over Na_2SO_4, filtered and concentrated in vacuo. The crude residue was purified via flash column chromatography.

1-Benzyl-5-methylindoline-2,3-dione (SM-1)

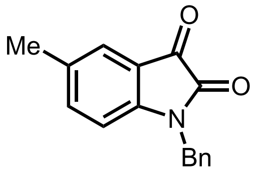

Synthesized from commercially available 5-methylindoline-2,3-dione following General Procedure A.

Yield: 72%

Purification: Eluted with 30% EtOAc in Hexanes (R_f = 0.42)

^{1}H NMR (400 MHz, $CDCl_3$) δ 7.52 (dd, J = 7.4, 1.4 Hz, 1H), 7.36 – 7.25 (m, 4H), 7.23 – 7.17 (m, 2H), 7.01 (t, J = 7.5 Hz, 1H), 5.19 (s, 2H), 2.25 (s, 3H).

^{13}C NMR (101 MHz, $CDCl_3$) δ 183.80, 159.72, 148.71, 142.67, 136.35, 128.72, 127.82, 125.78, 124.22, 123.80, 122.18, 118.95, 45.46, 18.78.

HRMS (ESI) m/z: calculated for $C_{16}H_{14}NO_2$ $[M+H]^+$: 252.1019. Found: 252.1020.

1-Benzyl-5-methoxyindoline-2,3-dione (SM-2)

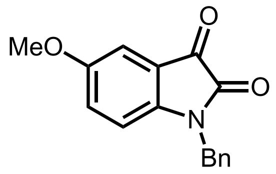

Synthesized from commercially available 5-methoxyindoline-2,3-dione following General Procedure A.

Yield: 68%

Purification: Eluted with 30% EtOAc in Hexanes (R_f = 0.31)

^{1}H NMR (400 MHz, $CDCl_3$) δ 7.33 (qd, J = 7.2, 3.5 Hz, 5H), 7.15 (d, J = 2.7 Hz, 1H), 7.02 (dd, J = 8.6, 2.7 Hz, 1H), 6.67 (d, J = 8.6 Hz, 1H), 4.91 (s, 2H), 3.77 (s, 3H).

^{13}C NMR (101 MHz, $CDCl_3$) δ 183.78, 158.50, 156.66, 144.74, 134.70, 129.18, 128.27, 127.54, 124.85, 118.21, 112.18, 109.64, 56.07, 44.19.

HRMS (ESI) m/z: calculated for $C_{16}H_{14}NO_3$ $[M+H]^+$: 268.0968. Found: 268.0968.

\

1-Benzyl-7-methylindoline-2,3-dione (SM-3)

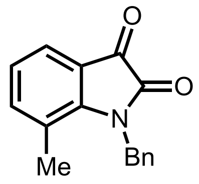

Synthesized from commercially available 7-methylindoline-2,3-dione following General Procedure A.

Yield: 78%

Purification: Eluted with 30% EtOAc in Hexanes (R_f = 0.43)

^{1}H NMR (400 MHz, $CDCl_3$) δ 7.42 (s, 1H), 7.35 – 7.26 (m, 6H), 6.65 (d, J = 8.1 Hz, 1H), 4.91 (s, 2H), 2.30 (s, 3H).

^{13}C NMR (101 MHz, $CDCl_3$) δ 183.67, 158.52, 148.65, 138.86, 134.74, 133.85, 129.14, 128.23, 127.53, 125.89, 117.80, 110.96, 44.14, 20.80.

HRMS (ESI) m/z: calculated for $C_{16}H_{14}NO_2$ $[M+H]^+$: 252.1019. Found: 252.1020.

Procedure for the Preparation of benzyl 2-oxopropanoate (SM-4):

The following procedure was adapted based on a reported literature procedure.[5] To a solution of pyruvic acid (10 mmol, 1.0 equiv), the alcohol (20 mmol, 2.0 equiv) and pyridine (25 mmol, 2.5 equiv) in THF (10 ml), methanesulfonyl chloride (12 mmol, 1.2 equiv) was added dropwise at 0 °C. The reaction was allowed to warm to room temperature and stirred for 18 h. After the indicated time, the reaction was quenched with water (30 ml) and extracted with Et_2O (30 ml × 3). The combined organic layers were washed with brine (50 mL), dried over Na_2SO_4, filtered and concentrated in vacuo. The crude residue was purified via flash column chromatography.

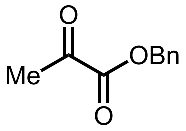

Yield: 35%

Purification: Eluted with 10% EtOAc in Hexanes (R_f = 0.32)

^{1}H NMR (400 MHz, $CDCl_3$) δ 7.44 – 7.34 (m, 5H), 5.28 (s, 2H), 2.48 (s, 3H).

^{13}C NMR (101 MHz, $CDCl_3$) δ 191.75, 160.62, 134.52, 128.96, 128.85, 128.81, 68.17, 26.92.

General Procedures for VHPO-Catalyzed Diazo Formation

General Analytical Procedure for the Synthesis of Diazo Compounds from Hydrazones (General Procedure B):

An enzyme aliquot of the VHPO from *Corallina pilulifera* (*Cp*VBPO, 10 µM, 50 µL) was removed from a -80 °C freezer and warmed to room temperature over 5 min. After thawing, a 250 mM solution of aqueous Na_3VO_4 (4 µL) was added to the enzyme aliquot, and the resulting mixture was centrifuged for 10 seconds using a Chemglass Life Sciences MLX-108-CLS mini centrifuge and then placed at room temperature until further use. To a 1-dram vial was then added H_2O purified by an Elga purification system (338.6 µL), 500 mM pH 6 citrate buffer (200 µL), and 176 mM aqueous KBr (3.41 µL, 0.3 equiv) followed by addition of 350 µL 2-Me THF. A 40 mM solution of corresponding hydrazone in 2-Me THF (50 µL, 1.0 equiv, 0.002 mmol substrate) was added. The aliquot containing the *Cp*VBPO (0.025 mol%, 1 µM in reaction) and Na_3VO_4 (0.5 equiv) was then added to the reaction mixture followed by the addition of a 10% stock of H_2O_2 (3.95 µL, 6.0 equiv). The vial was then capped and placed on a shaker at room temperature for 18 hr. After this time, the reaction mixture was diluted with MeCN (650 µL), transferred to an Eppendorf tube, and centrifuged in a Benchmark MC-24^{TM} Touch Centrifuge at 12,500 rpm for 5 min. After centrifugation, 650 µL of the top layer of the reaction mixture was transferred to an LCMS vial, which was then placed on an LCMS for analysis.*

**100 µL of 1,3,5-trimethoxybenzene was added as an internal standard for yield confirmation, where applicable.*

General Preparative Procedure for the Synthesis of Diazo Compounds from α-Ketoesters (General Procedure C):

A solution of hydrazine hydrate (50-60% in water, 51.8 µL, 0.8 mmol) was slowly added to a 1-dram vial containing a mixture of glacial acetic acid (100 µL) and water (100 µL) and cooled to 0° C in an ice bath. The starting α-ketoester (0.4 mmol) was added to the mixture and THF (100 µL) was added to homogenize the solution. The reaction was allowed to stir at room temperature at 1000 rpm for 18 hr. After this time, the reaction mixture was concentrated under reduced pressure to remove acetic acid and THF, yielding a crude hydrazone mixture that was transferred to a 250 mL round-bottom flask containing 2-Me THF (80 mL). An enzyme aliquot of VHPO from *Corallina pilulifera* (*Cp*VBPO, 10 µM, 10 mL) was taken from a -80 °C freezer, thawed at room temperature for 10 min, mixed with 800 µL of a 250 mM aqueous solution of Na_3VO_4, and was allowed to sit at room temperature for 30 minutes. The crude hydrazone mixture in the 250 mL flask was then combined with purified water (67.7 mL), 500 mM pH 6 citrate buffer (40 mL), and 176 mM aqueous KBr (682 µL, 0.3 equiv). The contents of the centrifuge tube containing *Cp*VBPO (0.0125 mol%, 0.5 µM in reaction) and Na_3VO_4 (0.5 equiv) was then added to the

reaction mixture followed by addition of 10% stock of H_2O_2 (790 μL, 6.0 equiv). The reaction was stirred for 18 hours at 900 rpm at room temperature. Upon completion, the resulting reaction mixture was washed three times with ethyl acetate (3 x 40 mL) to extract the product. The combined organic layers were then washed with brine (70 mL), dried over Na_2SO_4, and concentrated under reduced pressure. The resulting crude sample was purified via flash column chromatography to obtain the pure product.

General Preparative Procedure for the Synthesis of Diazo Compounds from α-Ketoamides (General Procedure D):

A solution of hydrazine hydrate (50-60% in water, 51.8 μL, 0.8 mmol) was slowly added to a 1-dram vial containing a mixture of glacial acetic acid (100 μL) and water (100 μL) and cooled to 0°C in an ice bath. The starting α-ketoamide (0.4 mmol) was added to the mixture and THF (300 μL) was added to homogenize the solution. The reaction was allowed to stir at room temperature at 1000 rpm for 18 hr. After this time, the reaction mixture was concentrated under reduced pressure to remove acetic acid and THF, yielding a crude hydrazone mixture that was transferred to a 250 mL round-bottom flask containing 2-Me THF (80 mL). An enzyme aliquot of VHPO from *Corallina pilulifera* (*Cp*VBPO, 10 μM, 10 mL) was taken from a -80 °C freezer, thawed at room temperature for 10 minutes, mixed with 800 μL of a 250 mM aqueous solution of Na_3VO_4, and allowed to sit at room temperature for 30 minutes. The crude hydrazone mixture in the 250 mL flask was then combined with purified water (67.7 mL), 500 mM pH 6 citrate buffer (40 mL), and 176 mM aqueous KBr (682 μL, 0.3 equiv). The contents of the centrifuge tube containing *Cp*VBPO (0.0125 mol%, 0.5 μM in reaction) and Na_3VO_4 (0.5 equiv) was then added to the reaction mixture followed by addition of 10% stock of H_2O_2 (790 μL, 6.0 equiv). The reaction was stirred for 18 hours at 900 rpm at room temperature. Upon completion, the reaction mixture was washed three times with ethyl acetate (3 x 40 mL) to extract the product. The combined organic layers were then washed with brine (70 mL), dried over Na_2SO_4, and concentrated under reduced pressure. The resulting crude sample was purified via flash column chromatography the pure product.

General Preparative Procedure for the Synthesis of Diazo Compounds from Benzophenones (General Procedure E):

A solution of hydrazine hydrate (50-60% in water, 256 μL, 4.0 mmol) was slowly added to a 1-dram vial containing a mixture of glacial acetic acid (50 μL). The starting benzophenone (0.4 mmol) and ethanol (500 μL) were then added to the mixture The reaction was allowed to stir at reflux for 18 hr. After this time, the reaction mixture was extracted with ethyl acetate (30 mL x 3). The combined organic layers were concentrated under reduced pressure, yielding a crude hydrazone mixture that was transferred to a 250 mL round-bottom flask containing 2-Me THF (80 mL). An enzyme aliquot of VHPO from *Corallina pilulifera* (*Cp*VBPO, 10 μM, 10 mL) was taken

from a -80 °C freezer, thawed at room temperature for 10 min, mixed with 800 µL of a 250 mM aqueous solution of Na_3VO_4, and was allowed to sit at room temperature for 30 minutes. The crude hydrazone mixture in the 250 mL flask was then combined with purified water (67.7 mL), 500 mM pH 8 tris buffer (40 mL), and 176 mM aqueous KBr (682 µL, 0.3 equiv). The contents of the centrifuge tube containing *Cp*VBPO (0.0125 mol%, 0.5 µM in reaction) and Na_3VO_4 (0.5 equiv) was then added to the reaction mixture followed by addition of 10% stock of H_2O_2 (790 µL, 6.0 equiv). The reaction was stirred for 18 hours at 900 rpm at room temperature. Upon completion, the resulting reaction mixture was washed three times with ethyl acetate (3 x 40 mL) to extract the product. The combined organic layers were then washed with brine (70 mL), dried over Na_2SO_4, and concentrated under reduced pressure. The resulting crude sample was purified via flash column chromatography using silica gel (pretreated with 5% NEt_3 in hexanes) to obtain the pure product.

Product Characterization for Diazo Compounds

Ethyl 2-diazo-2-phenylacetate (2)[6]

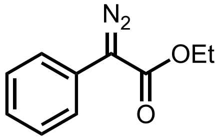

Synthesized from commercially available ethyl 2-oxo-2-phenylacetate following General Procedure C.

Yield: 83%

Purification: Eluted with 5% EtOAc in Hexanes (R_f = 0.46)

^{1}H NMR (400 MHz, $CDCl_3$) δ 7.54 – 7.45 (m, 2H), 7.43 – 7.35 (m, 2H), 7.23 – 7.13 (m, 1H), 4.34 (q, *J* = 7.1 Hz, 2H), 1.35 (t, *J* = 7.1 Hz, 3H).

^{13}C NMR (101 MHz, $CDCl_3$) δ 165.37, 129.05, 125.87, 125.75, 124.08, 61.12, 14.63.

HRMS (APCI) m/z: calculated for $C_{10}H_1O_2$ $[M+H-N_2]^+$: 163.07536. Found: 163.0751.

Standard Curve for Analytical Runs

Procedure for using standard curve is as follows: 1,3,5-trimethoxybenzene (8 mg/mL solution, 100 µL) is added to 900 µL of the reaction mixture and yield is determined by LCMS analysis based on the below standard curve. LCMS conditions: 2.5 µL injection volume, 0.5 mL/min mobile phase rate, 10-98% solvent B over 6.25 min. Mobile Phase: Solvent A – H_2O w/ 0.1% formic acid, Solvent B – MeCN w/ 0.1% formic acid.

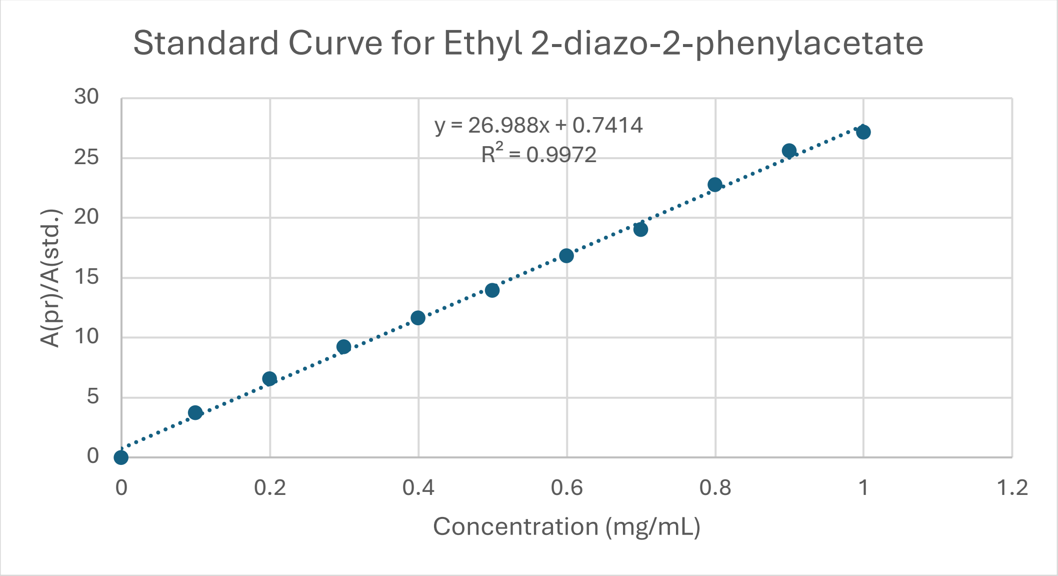

Ethyl 2-diazo-2-(p-tolyl)acetate (3)[7]

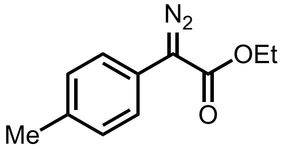

Synthesized from commercially available ethyl 2-oxo-2-(p-tolyl)acetate following General Procedure C.

Yield: 79%

Purification: Eluted with 5% EtOAc in Hexanes (R_f = 0.49)

^{1}H NMR (400 MHz, $CDCl_3$) δ 7.40 – 7.33 (m, 2H), 7.23 – 7.18 (m, 2H), 4.33 (q, J = 7.1 Hz, 2H), 2.34 (s, 3H), 1.34 (t, J = 7.1 Hz, 3H).

^{13}C NMR (101 MHz, $CDCl_3$) δ 165.65, 135.78, 129.79, 124.24, 122.39, 61.07, 21.14, 14.65.

HRMS (APCI) m/z: calculated for $C_{11}H_{13}O_2$ $[M+H-N_2]^+$: 177.0910. Found: 177.0906.

Ethyl 2-(4-(tert-butyl)phenyl)-2-diazoacetate (4)[6]

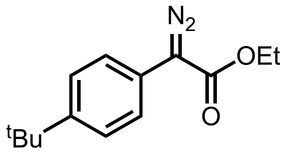

Synthesized from commercially available ethyl 2-(4-(tert-butyl)phenyl)-2-oxoacetate following General Procedure C.

Yield: 60%

Purification: Eluted with 5% EtOAc in Hexanes (R_f = 0.51)

^{1}H NMR (400 MHz, $CDCl_3$) δ 7.41 (s, 4H), 4.33 (q, J = 7.1 Hz, 2H), 1.36 – 1.33 (m, 3H), 1.32 (s, 9H).

^{13}C NMR (101 MHz, $CDCl_3$) δ 165.66, 149.10, 126.07, 124.12, 122.45, 61.09, 34.58, 31.39, 14.65.

HRMS (APCI) m/z: calculated for $C_{14}H_{19}O_2$ $[M+H-N_2]^+$: 219.1380. Found: 219.1375.

Ethyl 2-diazo-2-(4-methoxyphenyl)acetate (5)[7]

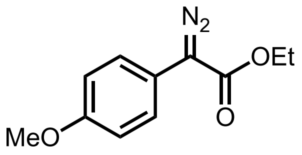

Synthesized from commercially available ethyl 2-(4-methoxyphenyl)-2-oxoacetate following General Procedure C.

Yield: 81%

Purification: Eluted with 5% EtOAc in Hexanes (R_f = 0.37)

^{1}H NMR (400 MHz, $CDCl_3$) δ 7.38 (d, J = 8.9 Hz, 2H), 6.94 (d, J = 9.0 Hz, 2H), 4.32 (q, J = 7.1 Hz, 2H), 3.81 (s, 3H), 1.33 (t, J = 7.1 Hz, 3H).

^{13}C NMR (101 MHz, $CDCl_3$) δ 165.93, 158.10, 126.05, 117.15, 114.70, 61.06, 55.47, 14.65.

HRMS (APCI) m/z: calculated for $C_{11}H_{13}O_3$ $[M+H-N_2]^+$: 193.0859. Found:193.0856.

Ethyl 2-(4-chlorophenyl)-2-diazoacetate (6)[8]

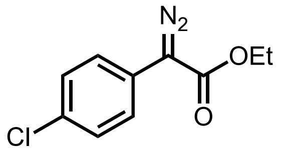

Synthesized from commercially available ethyl 2-(4-chlorophenyl)-2-oxoacetate following General Procedure C.

Yield: 73%

Purification: Eluted with 5% EtOAc in Hexanes (R_f = 0.54)

^{1}H NMR (400 MHz, $CDCl_3$) δ 7.48 – 7.39 (m, 2H), 7.38 – 7.30 (m, 2H), 4.33 (q, J = 7.1 Hz, 2H).

^{13}C NMR (101 MHz, $CDCl_3$) δ 165.04, 131.48, 129.18, 125.15, 124.40, 61.28, 14.60.

HRMS (APCI) m/z: calculated for $C_{10}H_{10}O_2{}^{35}Cl$ $[M+H-N_2]^+$: 197.0364. Found: 197.0362.

Ethyl 2-(4-bromophenyl)-2-diazoacetate (7)[8]

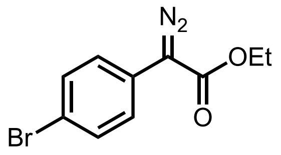

Synthesized from commercially available ethyl 2-(4-bromophenyl)-2-oxoacetate following General Procedure C.

Yield: 70%

Purification: Eluted with 5% EtOAc in Hexanes (R_f = 0.54)

^{1}H NMR (400 MHz, $CDCl_3$) δ 7.54 – 7.44 (m, 2H), 7.41 – 7.32 (m, 2H), 4.33 (q, *J* = 7.1 Hz, 2H), 1.34 (t, *J* = 7.1 Hz, 3H).

^{13}C NMR (101 MHz, $CDCl_3$) δ 164.98, 132.12, 125.44, 124.98, 119.36, 61.31, 14.61.

HRMS (APCI) m/z: calculated for $C_{10}H_{10}O_2{}^{79}Br$ $[M+H-N_2]^+$: 240.9859. Found: 240.9857.

Ethyl 2-diazo-2-(4-fluorophenyl)acetate (8)[8]

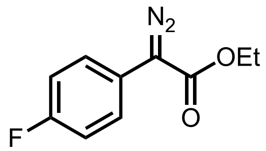

Synthesized from commercially available ethyl 2-(4-fluorophenyl)-2-oxoacetate following General Procedure C.

Yield: 74%

Purification: Eluted with 5% EtOAc in Hexanes (R_f = 0.52)

^{1}H NMR (400 MHz, $CDCl_3$) δ 7.50 – 7.39 (m, 2H), 7.13 – 7.05 (m, 2H), 4.33 (q, *J* = 7.1 Hz, 2H), 1.34 (t, *J* = 7.1 Hz, 3H).

^{13}C NMR (101 MHz, $CDCl_3$) δ 165.41, 161.06 (d, *J* = 237.2 Hz), 125.98 (d, *J* = 8.0 Hz), 121.50 (d, *J* = 3.2 Hz), 116.13 (d, *J* = 22.1 Hz), 61.23, 14.62.

HRMS m/z: calculated for $C_{10}H_{10}O_2F$ $[M+H-N_2]^+$: 181.0659. Found: 181.0657.

Ethyl 2-(4-cyanophenyl)-2-diazoacetate (9)[9]

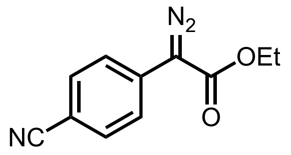

Synthesized from commercially available ethyl 2-(4-cyanophenyl)-2-oxoacetate following General Procedure C.

Yield: 65%

Purification: Eluted with 5% EtOAc in Hexanes (R_f = 0.17)

^{1}H NMR (400 MHz, $CDCl_3$) δ 7.69 – 7.54 (m, 4H), 4.34 (q, *J* = 7.1 Hz, 2H), 1.34 (t, *J* = 7.1 Hz, 3H).

^{13}C NMR (101 MHz, $CDCl_3$) δ 163.96, 132.62, 131.83, 123.44, 118.88, 108.60, 61.56, 14.50.

HRMS (APCI) m/z: calculated for $C_{11}H_{10}N_3O_2$ $[M+H]^+$: 216.0768. Found: 216.07644.

Ethyl 2-diazo-2-(3-methoxyphenyl)acetate (10)[10]

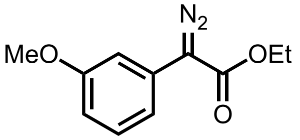

Synthesized from commercially available ethyl 2-(3-methoxyphenyl)-2-oxoacetate following General Procedure C.

Yield: 80%

Purification: Eluted with 5% EtOAc in Hexanes (R_f = 0.33)

^{1}H NMR (400 MHz, $CDCl_3$) δ 7.28 (t, *J* = 8.1 Hz, 1H), 7.17 (t, *J* = 2.2 Hz, 1H), 6.98 (ddd, *J* = 7.9, 1.8, 0.9 Hz, 1H), 6.72 (ddd, *J* = 8.3, 2.6, 0.9 Hz, 1H), 4.35 (d, *J* = 7.1 Hz, 2H), 3.82 (s, 3H), 1.34 (t, *J* = 7.1 Hz, 3H).

^{13}C NMR (101 MHz, $CDCl_3$) δ 165.28, 160.16, 129.96, 127.23, 116.06, 111.60, 109.69, 61.12, 55.37, 14.62.

HRMS (APCI) m/z: calculated for $C_{11}H_{13}O_3$ $[M+H-N_2]^+$: 193.0859. Found: 193.0856.

3-Diazo-1-phenylindolin-2-one (11)[11]

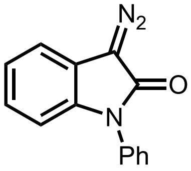

Synthesized from commercially available 1-phenylindoline-2,3-dione following General Procedure D.

Yield: 85%

Purification: Eluted with 30% EtOAc in Hexanes (R_f = 0.43)

^{1}H NMR (400 MHz, $CDCl_3$) δ 7.59 – 7.52 (m, 2H), 7.50 – 7.41 (m, 3H), 7.28 (ddd, *J* = 5.4, 2.7, 0.6 Hz, 1H), 7.18 – 7.12 (m, 2H), 6.98 – 6.91 (m, 1H).

^{13}C NMR (101 MHz, $CDCl_3$) δ 166.30, 134.51, 134.38, 129.73, 128.25, 126.92, 122.78, 118.41, 116.65, 110.09, 61.44.

HRMS (ESI) m/z: calculated for $C_{14}H_{10}N_3O$ $[M+H]^+$: 236.0818. Found: 236.0820.

3-Diazo-1-methylindolin-2-one (12)[11]

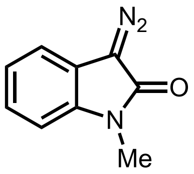

Synthesized from commercially available 1-methylindoline-2,3-dione following General Procedure D.

Yield: 64%

Purification: Eluted with 30% EtOAc in Hexanes (R_f = 0.26)

^{1}H NMR (400 MHz, $CDCl_3$) δ 7.23 – 7.14 (m, 2H), 7.08 (td, J = 7.4, 1.1 Hz, 1H), 6.94 – 6.87 (m, 1H), 3.32 (s, 3H).

^{13}C NMR (101 MHz, $CDCl_3$) δ 166.94, 134.58, 125.57, 122.19, 118.33, 116.79, 108.75, 26.92.

HRMS (APCI) m/z: calculated for $C_9H_8N_3O$ $[M+H]^+$: 174.0662. Found: 174.0663.

1-Benzyl-3-diazoindolin-2-one (13)[12]

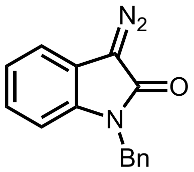

Synthesized from commercially available 1-benzylindoline-2,3-dione following General Procedure D.

Yield: 84%

Purification: Eluted with 30% EtOAc in Hexanes (R_f = 0.44)

^{1}H NMR (400 MHz, $CDCl_3$) δ 7.35 – 7.23 (m, 5H), 7.23 – 7.17 (m, 1H), 7.13 – 7.04 (m, 2H), 6.83 (dd, J = 7.8, 1.3 Hz, 1H), 5.03 (s, 2H).

^{13}C NMR (101 MHz, $CDCl_3$) δ 167.10, 136.19, 133.83, 128.98, 127.87, 127.50, 125.64, 122.37, 118.48, 116.96, 109.79, 44.49.

HRMS (APCI) m/z: calculated for $C_{15}H_{12}N_3O$ $[M+H]^+$: 250.0975. Found: 250.0976

1-Benzyl-3-diazo-5-methylindolin-2-one (14)[12]

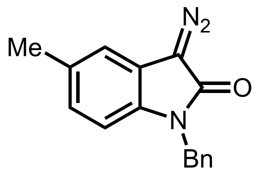

Synthesized from 1-Benzyl-5-methylindoline-2,3-dione (SM-1) following General Procedure D.

Yield: 80%

Purification: Eluted with 30% EtOAc in Hexanes (R_f = 0.48)

^{1}H NMR (400 MHz, $CDCl_3$) δ 7.24 – 7.14 (m, 5H), 6.95 (dt, *J* = 1.5, 0.7 Hz, 1H), 6.82 (ddd, *J* = 8.0, 1.7, 0.8 Hz, 1H), 6.63 (d, *J* = 8.0 Hz, 1H), 4.93 (s, 2H), 2.27 (s, 3H).

^{13}C NMR (101 MHz, $CDCl_3$) δ 167.07, 136.22, 131.95, 131.61, 128.84, 127.71, 126.19, 125.73 119.01, 116.89, 109.46, 60.81, 44.40, 21.27.

HRMS (ESI) m/z: calculated for $C_{16}H_{14}N_3O$ $[M+H]^+$: 264.1131. Found: 264.1133.

1-Benzyl-3-diazo-5-methoxyindolin-2-one (15)[12]

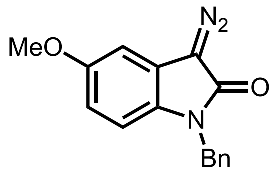

Synthesized from 1-Benzyl-5-methoxyindoline-2,3-dione (SM-2) following General Procedure D.

Yield: 78%

Purification: Eluted with 30% EtOAc in Hexanes (R_f = 0.36)

^{1}H NMR (400 MHz, $CDCl_3$) δ 7.24 – 7.12 (m, 5H), 6.68 (d, *J* = 2.4 Hz, 1H), 6.59 (d, *J* = 8.6 Hz, 1H), 6.53 (dd, *J* = 8.6, 2.4 Hz, 1H), 4.88 (s, 2H), 3.67 (s, 3H).

^{13}C NMR (101 MHz, $CDCl_3$) δ 166.85, 155.81, 136.19, 128.85, 127.73, 127.36, 117.91, 111.20, 110.17, 104.90, 61.36, 55.95, 44.47.

HRMS (ESI) m/z: calculated for $C_{11}H_{13}N_2O_3$ $[M+H]^+$: 280.1081. Found: 280.1082.

1-Benzyl-3-diazo-7-methylindolin-2-one (16)[13]

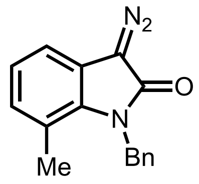

Synthesized from 1-Benzyl-7-methylindoline-2,3-dione (SM-3) following General Procedure A

Yield: 70%

Purification: Eluted with 30% EtOAc in Hexanes (R_f = 0.51)

^{1}H NMR (400 MHz, $CDCl_3$) δ 7.35 – 7.28 (m, 2H), 7.24 (t, *J* = 7.3 Hz, 1H), 7.18 – 7.11 (m, 2H), 7.08 (dd, *J* = 7.7, 1.3 Hz, 1H), 6.99 (t, *J* = 7.6 Hz, 1H), 6.90 – 6.81 (m, 1H), 5.31 (s, 2H), 2.31 (s, 3H).

^{13}C NMR (101 MHz, $CDCl_3$) δ 167.70, 138.00, 132.06, 129.39, 128.94, 127.32, 122.33, 120.84, 117.29, 116.37, 60.86, 45.64, 19.01.

HRMS (ESI) m/z: calculated for $C_{16}H_{14}N_3O$ $[M+H]^+$: 264.1131. Found: 264.11317.

3-Diazoindolin-2-one (17)[14]

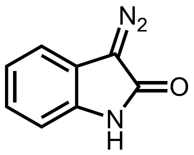

Synthesized from commercially available indoline-2,3-dione following General Procedure D.

Yield: 66%

Purification: Eluted with 50% EtOAc in Hexanes (R_f = 0.28)

^{1}H NMR (400 MHz, DMSO) δ 10.68 (s, 1H), 7.42 (dd, *J* = 7.7, 1.2 Hz, 1H), 7.10 (td, *J* = 7.7, 1.3 Hz, 1H), 7.00 (td, *J* = 7.6, 1.1 Hz, 1H), 6.91 (dt, *J* = 7.8, 0.8 Hz, 1H).

^{13}C NMR (101 MHz, DMSO) δ 168.29, 133.02, 125.62, 121.72, 119.79, 117.49, 110.41, 60.60.

HRMS (APCI) m/z: calculated for $C_8H_6N_3O$ $[M+H]^+$: 160.0505. Found: 160.0503.

Benzyl 2-diazopropanoate (18)[15]

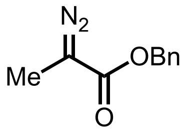

Synthesized from benzyl 2-oxopropanoate (SM-4) following General Procedure C.

Yield: 46%

Purification: Eluted with 5% EtOAc in Hexanes (R_f = 0.47)

^{1}H NMR (400 MHz, $CDCl_3$) δ 7.38 – 7.31 (m, 5H), 5.22 (s, 2H), 1.98 (s, 3H).

^{13}C NMR (101 MHz, $CDCl_3$) δ 167.96, 136.27, 128.65, 128.30, 128.16, 66.46, 29.55, 8.59.

Standard Curve for Analytical Runs

Procedure for using standard curve is as follows: 1,3,5-tribromobenzene (8 mg/mL solution, 100 µL) is added to 900 µL of the reaction mixture and yield is determined by LCMS analysis based on the below standard curve. LCMS conditions: 2.5 µL injection volume, 0.5 mL/min mobile phase rate, 10-98% solvent B over 6.25 min. Mobile Phase: Solvent A – H_2O w/ 0.1% formic acid, Solvent B – MeCN w/ 0.1% formic acid.

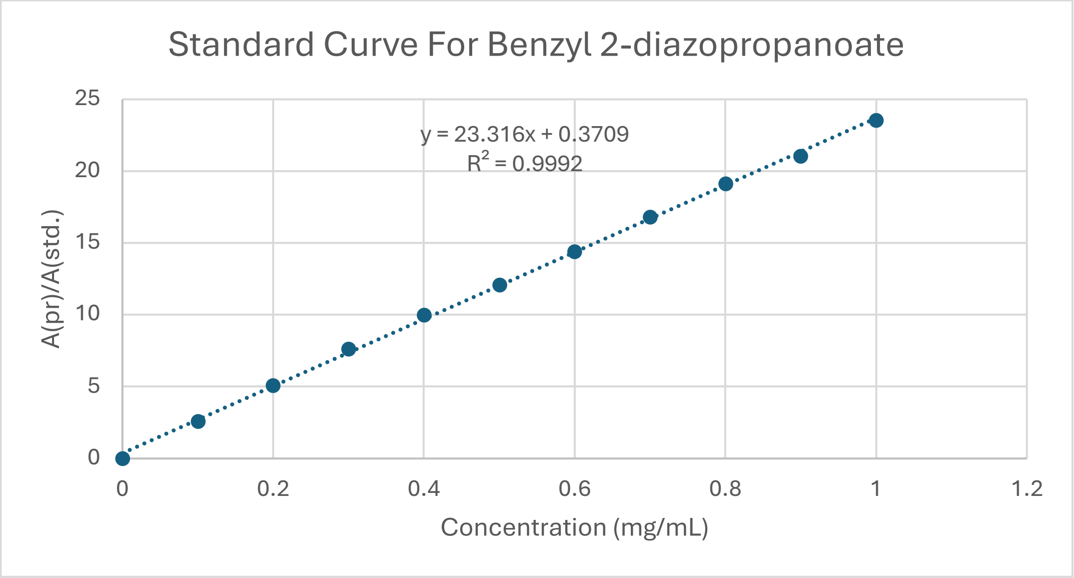

(Diazomethylene)dibenzene (19)[3]

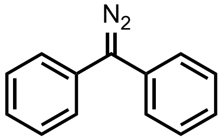

Synthesized from commercially available benzophenone following General Procedure E.

Yield: 18%

Purification: Eluted with 5% NEt_3 in Hexanes

^{1}H NMR (400 MHz, $CDCl_3$) δ 7.47 – 7.40 (m, 4H), 7.38 – 7.32 (m, 4H), 7.27 – 7.20 (m, 2H).

^{13}C NMR (101 MHz, $CDCl_3$) δ 129.62, 129.19, 125.67, 125.25.

1-(Diazo(phenyl)methyl)-4-methoxybenzene (20)[16]

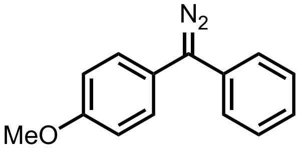

Synthesized from commercially available (4-Methoxyphenyl)(phenyl)methanone following General Procedure E.

Yield: 22%

Purification: Eluted with 5% NEt_3 in Hexanes

^{1}H NMR (400 MHz, $CDCl_3$) δ 7.41 – 7.35 (m, 2H), 7.33 – 7.27 (m, 2H), 7.25 – 7.19 (m, 2H), 7.19 – 7.12 (m, 1H), 7.02 – 6.96 (m, 2H), 3.85 (s, 3H).

^{13}C NMR (101 MHz, $CDCl_3$) δ 158.32, 130.49, 129.16, 127.80, 125.02, 124.24, 120.79, 114.93, 55.50.

1-(Diazo(4-methoxyphenyl)methyl)-4-nitrobenzene (21)[16]

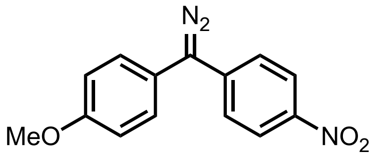

Synthesized from commercially available (4-Methoxyphenyl)(4-nitrophenyl)methanone following General Procedure E.

Yield: 11%

Purification: Eluted with 5% NEt_3 in Hexanes

^{1}H NMR (400 MHz, $CDCl_3$) δ 8.17 (d, J = 8.6 Hz, 2H), 7.40 – 7.29 (m, 2H), 7.20 – 7.11 (m, 2H), 7.02 (d, J = 8.2 Hz, 2H), 3.86 (s, 3H).

^{13}C NMR (101 MHz, $CDCl_3$) δ 159.70, 144.04, 139.93, 129.71, 124.75, 122.35, 118.61, 115.36, 55.60.

Additional Reaction Procedures

General Analytical Procedure for the Synthesis of Diazo Compounds using Wet Cell Lysate Expressing *Cp*VBPO (General Procedure F):

A wet cell lysate aliquot containing VHPO from *Corallina pilulifera* (*Cp*VBPO, 5 µL) was removed from a -80 °C freezer and warmed to room temperature over 5 min. After thawing, a 250 mM solution of aqueous Na_3VO_4 (4 µL) was added to the cell lysate aliquot, and the resulting mixture was centrifuged for 10 seconds using a Chemglass Life Sciences MLX-108-CLS mini centrifuge and then placed at room temperature until further use. To a 1-dram vial was then added H_2O purified by an Elga purification system (383.6 µL), 500 mM pH 6 citrate buffer (200 µL), and 176 mM aqueous KBr (3.41 µL, 0.3 equiv) followed by addition of 350 µL 2-Me THF. A 40 mM solution of corresponding hydrazone in 2-Me THF (50 µL, 1.0 equiv, 0.002 mmol substrate) was added. The cell lysate aliquot containing the *Cp*VBPO (5 µL) and Na_3VO_4 (0.5 equiv) was then added to the reaction mixture followed by the addition of a 10% stock of H_2O_2 (3.95 µL, 6.0 equiv). The vial was then capped and placed on a shaker at room temperature for 18 hr. After this time, the reaction mixture was diluted with MeCN (650 µL), transferred to an Eppendorf tube, and centrifuged in a Benchmark MC-24™ Touch Centrifuge at 12,500 rpm for 5 min. After centrifugation, 650 µL of the top layer of the reaction mixture was transferred to an LCMS vial, which was then placed on an LCMS for analysis.*

**100 µL of 1,3,5-trimethoxybenzene was added as an internal standard for yield confirmation, where applicable.*

General Analytical Procedure for the Synthesis of Diazo Compounds using Whole Cells Expressing *Cp*VBPO (General Procedure G):

An aliquot containing whole *E. coli* cell expressing VHPO from *Corallina pilulifera* (*Cp*VBPO, 5 µL, adjusted to OD_{600} = 18.5) was removed from a -80 °C freezer and warmed to room temperature over 5 min. After thawing, a 250 mM solution of aqueous Na_3VO_4 (4 µL) was added to the whole cell aliquot, and the resulting mixture was centrifuged for 10 seconds using a Chemglass Life Sciences MLX-108-CLS mini centrifuge and then placed at room temperature until further use. To a 1-dram vial was then added H_2O purified by an Elga purification system (383.6 µL), 500 mM pH 6 citrate buffer (200 µL), and 176 mM aqueous KBr (3.41 µL, 0.3 equiv) followed by addition of 350 µL 2-Me THF. A 40 mM solution of corresponding hydrazone in 2-Me THF (50 µL, 1.0 equiv, 0.002 mmol substrate) was added. The aliquot containing the whole *E. coli* cells expressing *Cp*VBPO (5 µL, adjusted to OD_{600} = 18.5) and Na_3VO_4 (0.5 equiv) was then added to the reaction mixture followed by the addition of a 10% stock of H_2O_2 (3.95 µL, 6.0 equiv). The vial was then capped and placed on a shaker at room temperature for 18 hr. After this time, the reaction mixture was diluted with MeCN (650 µL), transferred to an Eppendorf tube, and

centrifuged in a Benchmark MC-24^{TM} Touch Centrifuge at 12,500 rpm for 5 min. After centrifugation, 650 µL of the top layer of the reaction mixture was transferred to an LCMS vial, which was then placed on an LCMS for analysis.*

**100 µL of 1,3,5-trimethoxybenzene was added as an internal standard for yield confirmation, where applicable.*

General Analytical Procedure for the Synthesis of Diazo Compounds using Lyophilized Cell Lysate Expressing *Cp*VBPO (General Procedure H):

To a PCR tube, 0.3 mg of lyophilized cell lysate was added followed by addition of 500 mM pH 6 citrate buffer (100 µL) and 250 mM solution of aqueous Na_3VO_4 (4 µL). The resulting mixture was centrifuged for 10 seconds using a Chemglass Life Sciences MLX-108-CLS mini centrifuge and then placed at room temperature until further use. To a 1-dram vial was then added H_2O purified by an Elga purification system (388.6 µL), 500 mM pH 6 citrate buffer (100 µL), and 176 mM aqueous KBr (3.41 µL, 0.3 equiv) followed by addition of 350 µL 2-Me THF. A 40 mM solution of corresponding hydrazone in 2-Me THF (50 µL, 1.0 equiv, 0.002 mmol substrate) was added. The aliquot containing the lyophilized cell lysate and Na_3VO_4 (0.5 equiv) was then added to the reaction mixture followed by the addition of a 10% stock of H_2O_2 (3.95 µL, 6.0 equiv). The vial was then capped and placed on a shaker at room temperature for 18 hr. After this time, the reaction mixture was diluted with MeCN (650 µL), transferred to an Eppendorf tube, and centrifuged in a Benchmark MC-24^{TM} Touch Centrifuge at 12,500 rpm for 5 min. After centrifugation, 650 µL of the top layer of the reaction mixture was transferred to an LCMS vial, which was then placed on an LCMS for analysis.*

**100 µL of 1,3,5-trimethoxybenzene was added as an internal standard for yield confirmation, where applicable.*

General Analytical Procedure for the Synthesis of Diazo Compounds using Lyophilized Whole Cells Expressing *Cp*VBPO (General Procedure I):

To a PCR tube, 0.4 mg of lyophilized whole cell was added followed by addition of 500 mM pH 6 citrate buffer (100 µL) and 250 mM solution of aqueous Na_3VO_4 (4 µL). The resulting mixture was centrifuged for 10 seconds using a Chemglass Life Sciences MLX-108-CLS mini centrifuge and then placed at room temperature until further use. To a 1-dram vial was then added H_2O purified by an Elga purification system (388.6 µL), 500 mM pH 6 citrate buffer (100 µL), and 176 mM aqueous KBr (3.41 µL, 0.3 equiv) followed by addition of 350 µL 2-Me THF. A 40 mM solution of corresponding hydrazone in 2-Me THF (50 µL, 1.0 equiv, 0.002 mmol substrate) was added. The aliquot containing the lyophilized whole cell and Na_3VO_4 (0.5 equiv) was then added to the reaction mixture followed by the addition of a 10% stock of H_2O_2 (3.95 µL, 6.0 equiv).

The vial was then capped and placed on a shaker at room temperature for 18 hr. After this time, the reaction mixture was diluted with MeCN (650 µL), transferred to an Eppendorf tube, and centrifuged in a Benchmark MC-24™ Touch Centrifuge at 12,500 rpm for 5 min. After centrifugation, 650 µL of the top layer of the reaction mixture was transferred to an LCMS vial, which was then placed on an LCMS for analysis.*

**100 µL of 1,3,5-trimethoxybenzene was added as an internal standard for yield confirmation, where applicable.*

General Preparative Procedure for the Synthesis of Diazo Compounds from α-Ketoesters using Whole Cells Expressing *Cp*VBPO (General Procedure J):

A solution of hydrazine hydrate (50-60% in water, 51.8 µL, 0.8 mmol) was slowly added to a 1-dram vial containing a mixture of glacial acetic acid (100 µL) and water (100 µL) cooled in an ice bath. The α-keto-ester (0.4 mmol) was added to the mixture and THF (100 µL) was added to homogenize the solution. The reaction was allowed to stir at room temperature at 1000 rpm for 18 hr. After this time, the reaction mixture was concentrated under reduced pressure to remove acetic acid and THF, yielding a crude hydrazone mixture, which was transferred to a 250 mL round-bottom flask containing 2-Me THF (80 mL). A whole cell aliquot expressing VHPO from *Corallina pilulifera* (*Cp*VBPO, 1 mL, adjusted to OD_{600} = 18.5) was taken from a -80 °C freezer, thawed at room temperature for 10 minutes, and then mixed with 800 µL of a 250 mM aqueous solution of Na_3VO_4, allowing it to sit at room temperature for 30 minutes. The crude hydrazone mixture in the 250 mL flask was then combined with purified water (77.7 mL), 500 mM pH 6 citrate buffer (40 mL), and 176 mM aqueous KBr (682 µL, 0.3 equiv). The contents of the centrifuge tube containing whole cell expressing *Cp*VBPO and Na_3VO_4 (0.5 equiv) was then added to the reaction mixture followed by addition of 10% stock of H_2O_2 (790 µL, 6.0 equiv). The reaction mixture was stirred at room temperature for 18 hours at 900 rpm. After completion, the product was extracted using ethyl acetate (100 mL). The mixture was divided among six 50 mL centrifuge tubes and centrifuged (Sorvall ST Plus) at 13,000 rpm for 20 minutes at 10 °C to separate the organic layer from the aqueous layer. The aqueous layer underwent five additional extractions with ethyl acetate (50 mL each). The combined organic layers were then washed with brine (100 mL), dried over sodium sulfate, and concentrated under reduced pressure. The resulting crude sample was purified via flash column chromatography to obtain the pure product.

General Preparative Procedure for One Pot Transesterification, Hydrazone Formation and Diazo Formation (General Procedure K):

To a 1-dram vial, ethyl 2-oxo-2-phenylacetate (63.9 µL, 0.4 mmol) was added, followed by addition of corresponding alcohol (1 mL) and *Candida antarctica* lipase B (CalB immo Plus™, 10.0 mg). The reaction mixture was allowed to stir at room temperature for 16 hr at 900 rpm. Upon

completion of the reaction, the mixture was filtered through a Celite pad and concentrated in *vacuo*. The crude α-keto-ester mixture was then added to a 1-dram vial containing a stirred solution of hydrazine hydrate (50-60% in water, 51.8 μL, 0.8 mmol), glacial acetic acid (100 μL) and water (100 μL) followed by addition of THF (100 μL) to homogenize the solution. The reaction was allowed to stir at room temperature at 1000 rpm for 18 hr. After this time, the reaction mixture was concentrated under reduced pressure to remove acetic acid and THF, yielding a crude hydrazone mixture, which was transferred to a 250 mL round-bottom flask containing 2-Me THF (80 mL). An enzyme aliquot of VHPO from *Corallina pilulifera* (*Cp*VBPO, 10 μM, 10 mL) was taken from a -80 °C freezer, thawed at room temperature for 10 minutes, and then mixed with 800 μL of a 250 mM aqueous solution of Na_3VO_4, allowing it to sit at room temperature for 30 minutes. The crude hydrazone mixture in the 250 mL flask was then combined with purified water (67.7 mL), 500 mM pH 6 citrate buffer (40 mL), and 176 mM aqueous KBr (682 μL, 0.3 equiv). The contents of the centrifuge tube containing *Cp*VBPO (0.0125 mol%, 0.5 μM in reaction) and Na_3VO_4 (0.5 equiv) was then added to the reaction mixture followed by addition of 10% stock of H_2O_2 (790 μL, 6.0 equiv). The reaction was stirred for 18 hours at 900 rpm at room temperature. Upon completion, the reaction mixture was washed three times with ethyl acetate (3 x 40 mL) to extract the product. The combined organic layers were then washed with brine (70 mL), dried over sodium sulfate, and concentrated under reduced pressure. The resulting crude sample was purified via flash column chromatography to obtain the pure product.

Methyl 2-diazo-2-phenylacetate (23)[6]

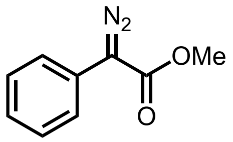

Synthesized from commercially available ethyl 2-oxo-2-phenylacetate following General Procedure K.

Yield: 79%

Purification: Eluted with 5% EtOAc in Hexanes (R_f = 0.45)

^{1}H NMR (400 MHz, $CDCl_3$) δ 7.54 – 7.44 (m, 2H), 7.43 – 7.36 (m, 2H), 7.23 – 7.15 (m, 1H), 3.87 (s, 3H).
^{13}C NMR (101 MHz, $CDCl_3$) δ 165.62, 128.97, 125.86, 125.49, 123.98, 52.01.

HRMS (APCI) m/z: calculated for $C_9H_{11}O_2$ $[M+H-N_2]^+$: 151.07536. Found: 151.0752.

Propyl 2-diazo-2-phenylacetate (24)

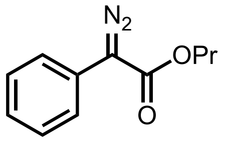

Synthesized from commercially available ethyl 2-oxo-2-phenylacetate following General Procedure K.

Yield: 75%

Purification: Eluted with 5% EtOAc in Hexanes ($R_f = 0.46$)

^{1}H NMR (400 MHz, $CDCl_3$) δ 7.54 – 7.44 (m, 2H), 7.42 – 7.35 (m, 2H), 7.22 – 7.15 (m, 1H), 4.24 (t, $J = 6.7$ Hz, 2H), 1.74 (dtd, $J = 14.1, 7.4, 6.6$ Hz, 2H), 0.99 (t, $J = 7.4$ Hz, 3H).

^{13}C NMR (101 MHz, $CDCl_3$) δ 165.42, 129.05, 125.88, 125.79, 124.10, 66.65, 22.34, 10.50.

HRMS (APCI) m/z: calculated for $C_{11}H_{13}O_2$ $[M+H-N_2]^+$: 177.09101. Found: 177.09071.

Isopropyl 2-diazo-2-phenylacetate (25)[6]

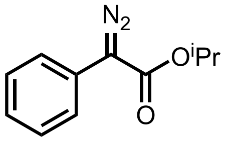

Synthesized from commercially available ethyl 2-oxo-2-phenylacetate following General Procedure K with the use of 30 mg CalB.

Yield: 74%

Purification: Eluted with 5% EtOAc in Hexanes ($R_f = 0.47$)

^{1}H NMR (400 MHz, $CDCl_3$) δ 7.57 – 7.45 (m, 2H), 7.38 (t, $J = 7.9$ Hz, 2H), 7.22 – 7.13 (m, 1H), 5.21 (hept, $J = 6.3$ Hz, 1H), 1.33 (d, $J = 6.3$ Hz, 6H).

^{13}C NMR (101 MHz, $CDCl_3$) δ 164.97, 129.03, 125.91, 125.80, 124.07, 68.79, 22.21.

HRMS (APCI) m/z: calculated for $C_{11}H_{13}O_2$ $[M+H-N_2]^+$: 177.0910. Found: 177.0906.

Butyl 2-diazo-2-phenylacetate (26)

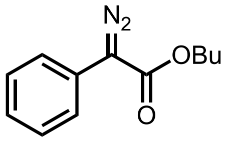

Synthesized from commercially available ethyl 2-oxo-2-phenylacetate following General Procedure K.

Yield: 75%

Purification: Eluted with 5% EtOAc in Hexanes (R_f = 0.47)

^{1}H NMR (400 MHz, $CDCl_3$) δ 7.49 (dd, J = 8.4, 1.4 Hz, 2H), 7.38 (t, J = 7.9 Hz, 2H), 7.21 – 7.14 (m, 1H), 4.28 (t, J = 6.6 Hz, 2H), 1.74 – 1.65 (m, 2H), 1.43 (h, J = 7.4 Hz, 2H), 0.96 (t, J = 7.4 Hz, 3H).

^{13}C NMR (101 MHz, $CDCl_3$) δ 165.44, 129.05, 125.87, 125.76, 124.06, 64.96, 30.97, 19.26, 13.87.

HRMS (APCI) m/z: calculated for $C_{12}H_{15}O_2$ $[M+H-N_2]^+$: 2191.10666. Found: 191.10643.

Optimization Data

All optimization reactions for the synthesis of Ethyl 2-diazo-2-phenylacetate (**2**) were performed using General Procedure B). The only variable changed is the one indicated in the Figures below. (*Note: 100 µL of a 8 mg/mL solution of 1,3,5-trimethoxybenzene was added as an internal standard for yield confirmation, where applicable).*

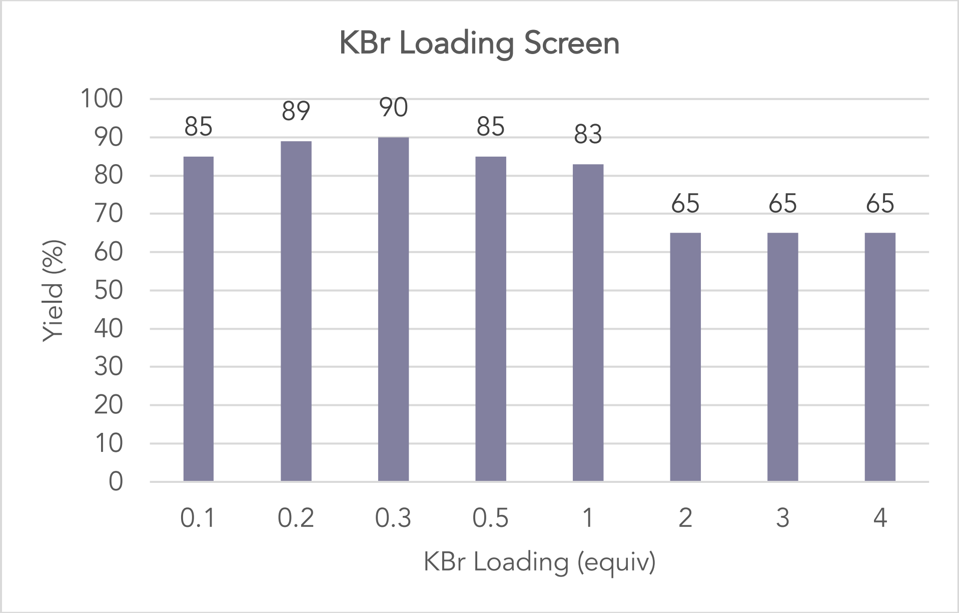

Figure S1. Potassium Bromide (KBr) Loading Screen

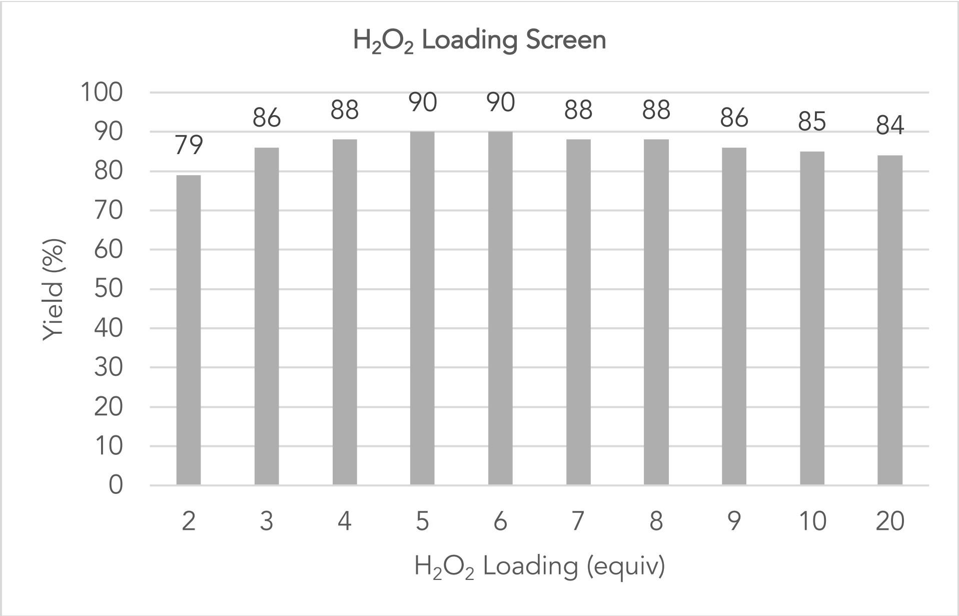

Figure S2. Hydrogen Peroxide (H_2O_2) Loading Screen

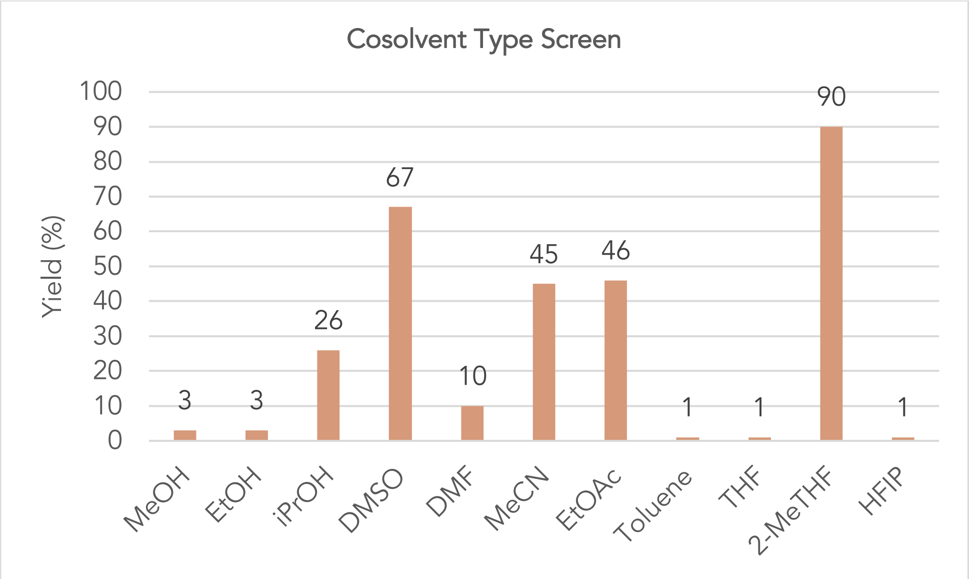

Figure S3. Cosolvent Type Screen

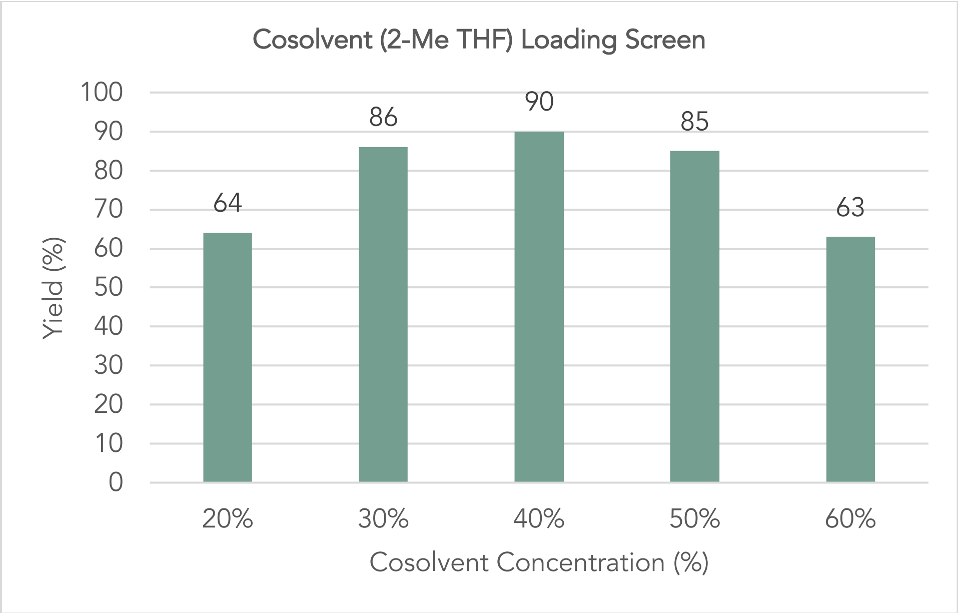

Figure S4. Cosolvent (2-MeTHF) Loading Screen

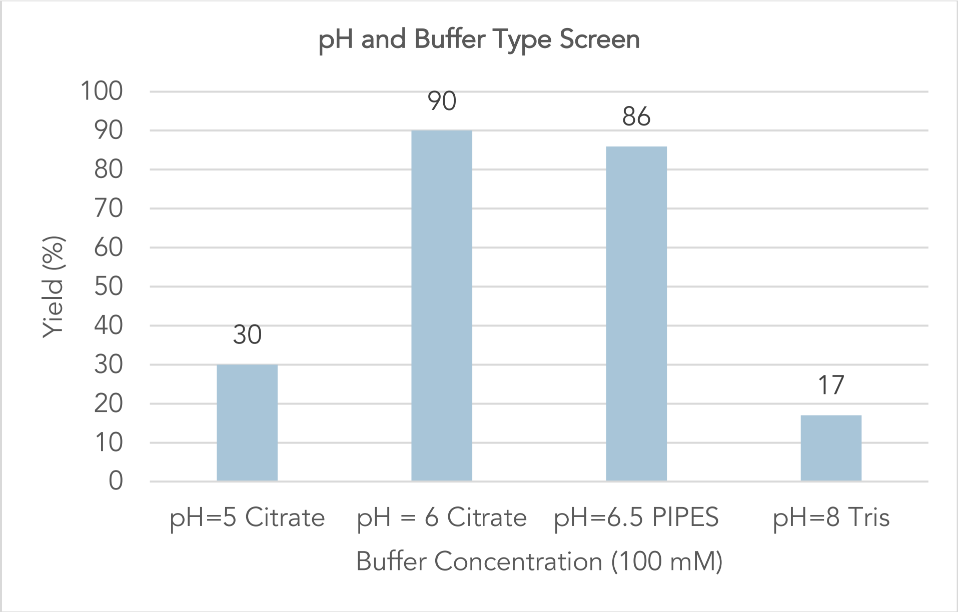

Figure S5. pH and Buffer Type Screen

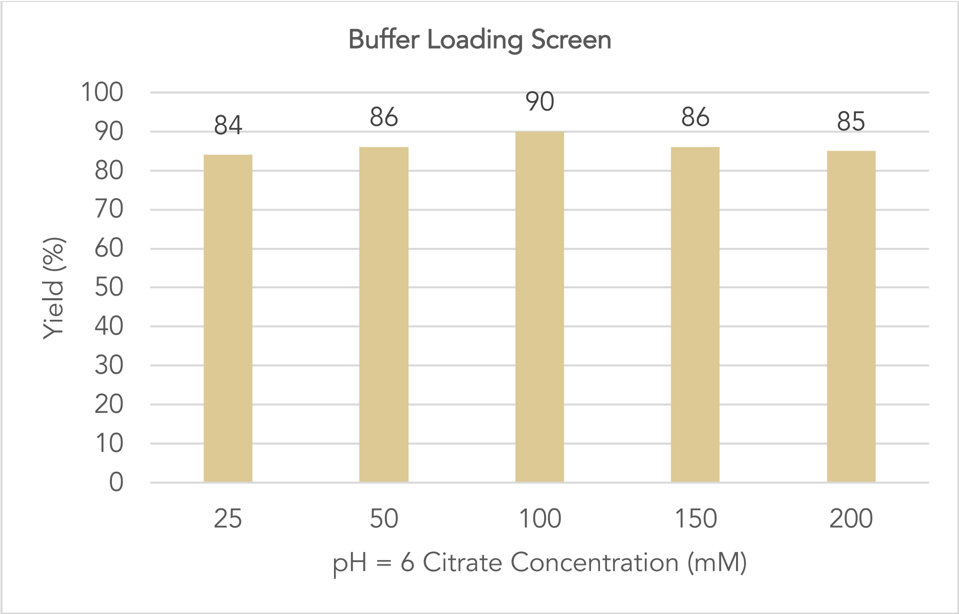

Figure S6. Buffer Loading Screen

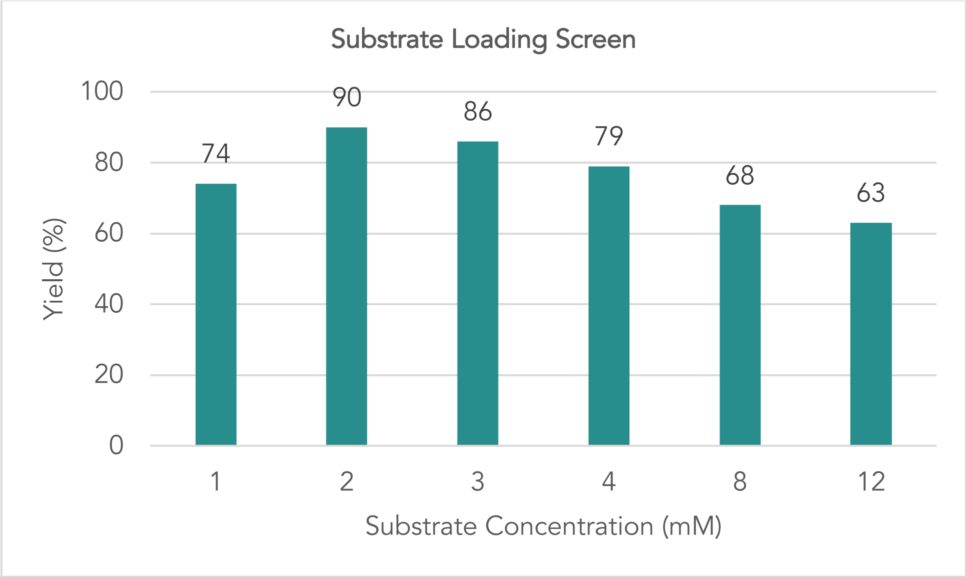

Figure S7. Substrate Loading Screen

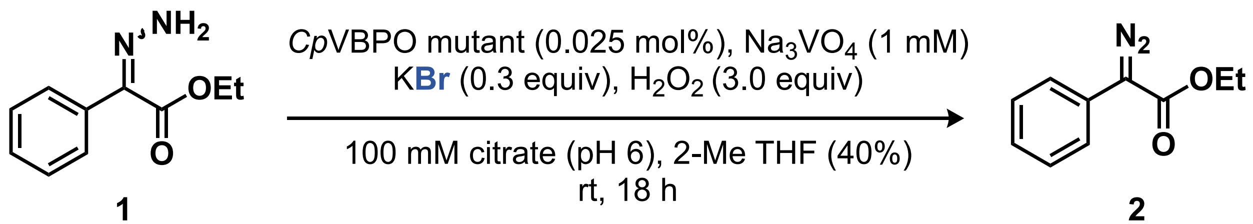

1 **2**

**Mutant**	**E/Z-mixture (1/3) Yield (%)**	**(E)-Isomer Yield (%)**	**(Z)-Isomer Yield (%)**
Cp D335G	0%	0%	0%
Cp A336G	90%	93%	87%
Cp L337G	33%	50%	15%
Cp F373G	90%	93%	87%
Cp S378A	90%	93%	87%

Figure S8. Biocatalytic hydrazone oxidation with the *Cp*VBPO mutants

Molecular Docking and Dynamics Experiments

Molecular Docking: Substrates [**(Z)-1** and **(E)-1**] were parameterized using SwissParam[17] and docked to the crystal structure of bromoperoxidase from *Corallina pilulifera* after initial processing. A model of the catalytic dimer (chains A and B) was extracted from PDB accession code 7QWI[18] and prepared for input into AutoDock[19-21] by deleting alternate residue confirmations, as well as removing small molecules, ions not associated with the active site, and solvent. Unnatural amino acids, e.g. BYR and HSE, were additionally converted to canonical amino acids. Active site ions relevant for catalysis were retained; however, vanadate was converted to phosphate to simplify parametrization. AutoDockTools was used to assign partial charges and generate suitable AutoDock input files for both the ligand and receptor. Charges for the active-site ions were then manually adjusted as follows: -0.75 for phosphate each O, 0 for phosphate P, -1 for Br. To minimize spurious binding modes distant from the enzyme's active site, we defined a ~32,000 Å^3 tetragonal search space encapsulating the entire interface between the two dimers. All ligand bonds were set as rigid, and no flexible receptor residues were defined. Note that the active site of the bromoperoxidase is formed at the interface of two monomers, which is vital to properly simulating the binding of substrates. To maintain a traditional two-body docking protocol and maintain the integrity of the binding site, these chains were relabeled as a single large molecule before submission to AutoDock. Simulations utilized AutoDock's Lamarckian Genetic Algorithm[22] to assess ligand conformations for the lowest energy interactions. The resultant docking models were then clustered based on their similarity and ranked according to disperson/repulsion energies, hydrogen bonding interactions, electrostatics, and desolvation potential.[22] The program was set to 150 runs. A representative structure from the top ranked cluster, having the most energetically favored interactions with each docked substrate, was then analyzed in PyMOL.[23]

Vanadate Metal Center Parameterization and Molecular Dynamics: To gain insight into the interactions underlying **(Z)-1** and **(E)-1** binding by *Cp*VBPO, molecular dynamics (MD) of the docked enzyme-substrate complex were carried out. Using GROMACS v2019.3 and the AMBER99SB-ILDN forcefield.[24-27] All complexes were placed in an octahedral box with the edge of the box placed 1 nm from the outer edge of the centered complex coordinates, which was solvated with water described by the SPC/E model.[28-29] The complexes were neutralized with KBr ions at a concentration of 50 mM and energy minimized with a maximum force constant set to 1000 kJ/mol/nm using the steepest descent algorithm. NVT equilibration was carried out using the V-rescale modified Berendsen thermostat,[30] with the target temperature set to 300 K and the temperature time constant set to 0.1 ps. Equilibration was carried out for 100 ps at a 2-fs integration time-step with position restraints placed on the enzyme-substrate complexes. A restraining force of 1000 kJ/mol/nm was placed on all heavy atoms of the complexes. Two temperature-coupled groups, the enzyme(substrates) complex and the ionized solvent, were defined during the equilibration. Covalent bonds involving hydrogen in this, and the following steps, were constrained using the LINCS algorithm.[31-32] Both PME[33] and vdW neighbor cutoffs for calculation of the respective forces were set to 10 Å. NPT equilibration of the systems was carried out in a two-step manner with position restraints placed on the substrate first and none on the enzyme, equilibrated for 1 ns, followed by position restraints on the enzyme with none on the substrate and

a subsequent equilibration for 1 ns. Parameters such as cutoffs, coupling groups, and integration time-steps were the same as for NVT, while using the Berendsen barostat[34] with a target pressure of 1 bar and isothermal compressibility of water set to 4.5 x 10^5 bar^{-1}. Production molecular dynamics were performed with no position restraints and utilized the V-rescale thermostat and the Parrinello-Rahman barostat,[35] with all other parameters the same as for NPT. All equilibrations and molecular dynamics simulations used the vdW interaction modifier option Potential-Shift and included the dispersion correction option EnerPres.

Initial simulations were carried out using phosphate in place of vanadate. This attracted multiple positive ions into the active site and created unrealistic configurations when compared to the original crystal structure. Hydrogenated versions of the phosphate continuously yielded errors in the LINCS algorithm, which typically points to underlying fundamental issues with parameterization and molecular configuration. This led us to attempt parameterization of the vanadate metal center as has been done previously. Using the published parameters also yielded LINCS errors, with the apical O-H seemingly the underlying issue. Closer inspection of the parameters raised multiple concerns over values such as V-O bond force constants being equivalent between V-O and V-OH bonds, and a rather high force constant between the His553 N and the vanadate V. We then attempted to parameterize the orthovanadate metal center ourselves using the Metal Center Parameter Builder python script (MCPB.py).[36] All Density Functional Theory methods for MCPB.py were carried out in Gaussian16[37] using the B3LYP[38-40] functional, the LAN2DZ[41-42] basis and pseudo potential applied to V, and the 6-31G*[43] basis applied to the other atoms. Force constants were derived via the Seminario method[44] using the Hessian of a DFT optimized model of His553 bound to orthovanadate truncated to its α-carbon. The α-carbon was changed to a methyl group. The partial charges were calculated from a 2-step Restricted Electrostatic Potential (RESP)[45] fitting of a Merz-Singh-Kollman (MK) derived ESP grid.[46-47] The ESP model contained His553, orthovanadate, and methyl capped His553 amide atoms. Our parameters for His553 and the bound metal center can be found in **Table 1**. As with the previous parameters, we ran into LINCS errors at NPT equilibration even at an integration time step of 0.5 fs. Rather than lower the accuracy of the algorithm, we considered other possibilities for the vanadate protonation and coordination state.

The question of the true protonation and coordination state of vanadate in the catalytic form of VHPOs is still a matter of debate. The parameters derived previously and by us for MD simulations were based on a diprotonated version of orthovanadate within the context of *Curvularia inaequalis* VCPO.[48-50] However, there is evidence to suggest that this may not be the true protonation state at the catalytically optimal pH of 6, and there are fundamental differences in active site residues between our enzyme and VCPO.[51-53] Using the proposal set forth by Anderson et. al.,[53] we sought to simulate a triprotonated center. Towards this end, we parameterized a monoprotonated metavanadate metal center, keeping all MM waters equivalent and described by SPC/E. We hypothesized that this would introduce a coordination of water to the metal center over the course of simulations. This metal center parameterization resolved the occurrence of PME and LINCS errors upon NPT equilibration, so we moved forward with this model for all simulations.

Parameters for **(Z)-1** and **(E)-1** were obtained by first DFT optimization of each substrate model, followed by an Merz-Singh-Kollman analysis, both using the B3LYP [39,54] functional and 6-31G*[43] basis for all atoms. The generated ESP grid was then subjected to a 2-step RESP[45] fitting protocol to obtain partial charges and other parameters for the forcefield. The parameters for the substrates were supplemented with dihedral restraints on the isomeric dihedral to prevent spontaneous isomerization during simulation.

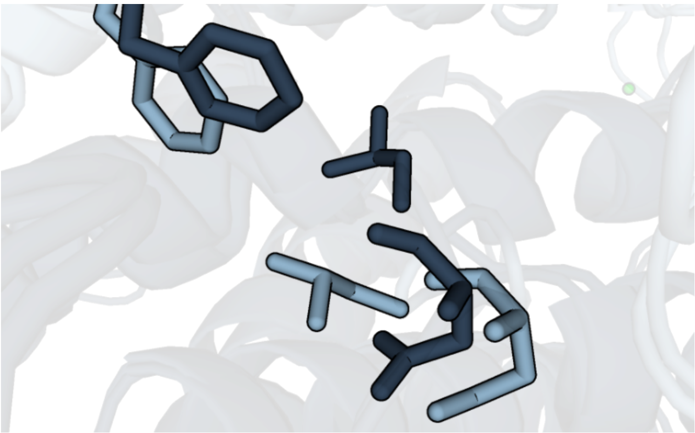

Figure S9. Overlay of ligand binding residues when **(E)-1** (light blue) vs. **(Z)-1** (dark blue) is bound after 50 ns of MD simulations. Leu337 seems to sample the "open" conformation in some frames when the **(Z)-1** hydrazone is nearby.

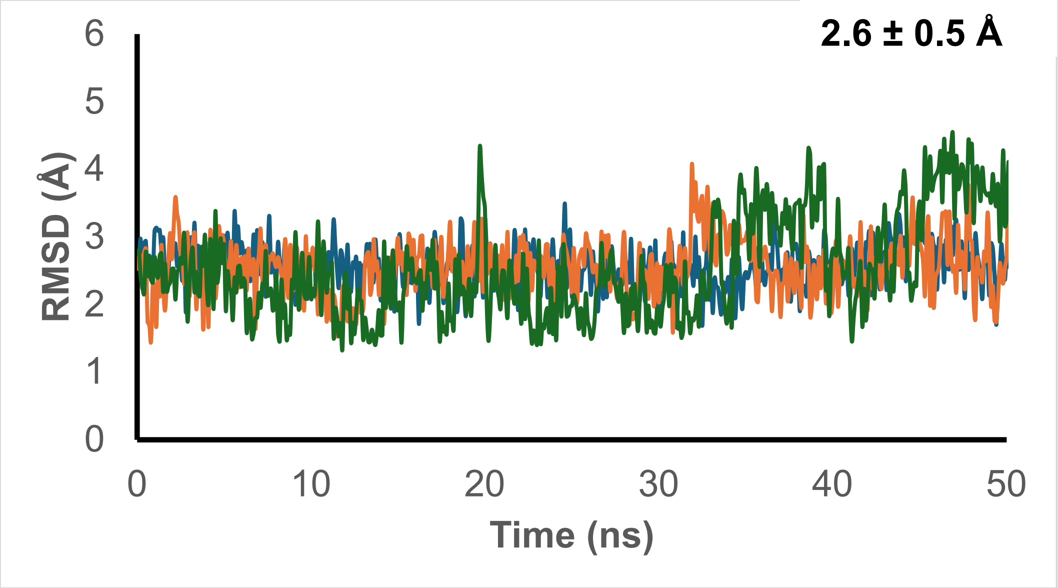

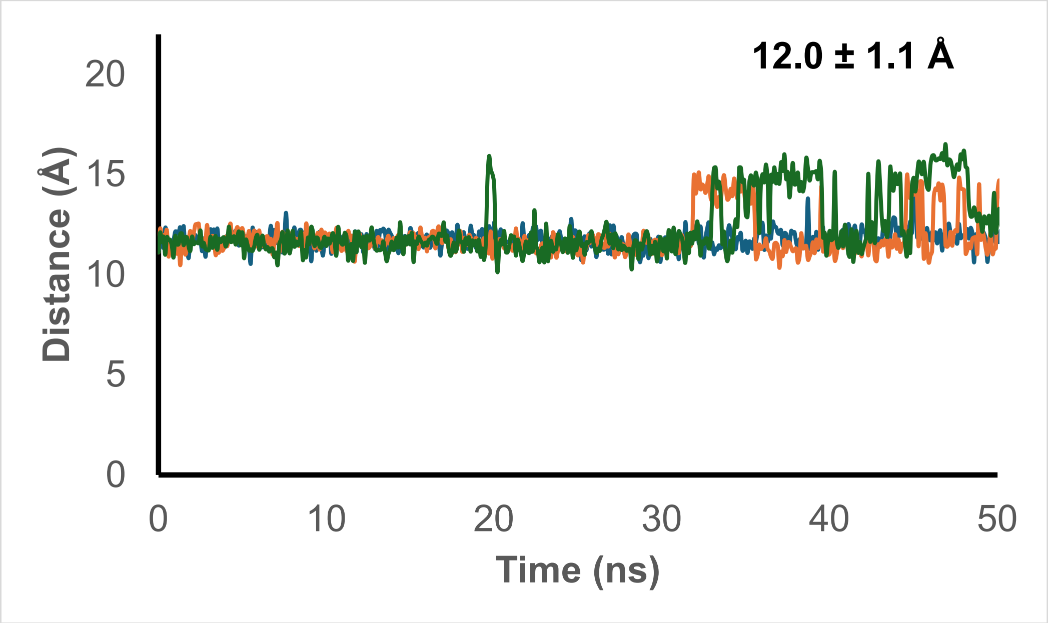

Figure S10. RMSD and distance from vanadate analysis of (E)-1 hydrazone over 50 ns MD simulations in the context of the D335G.

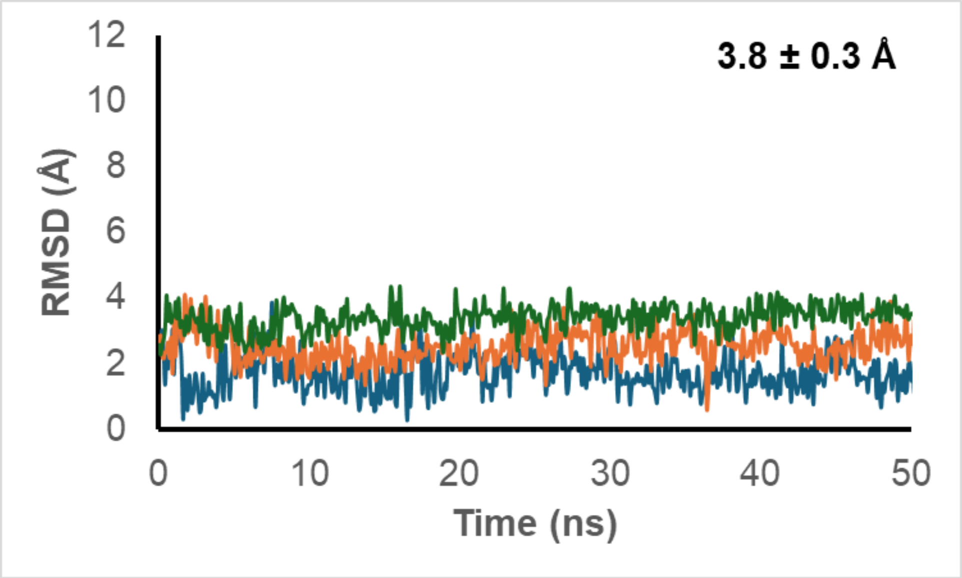

Figure S11. RMSD analysis of the active site Br^- ion over 50 ns MD simulations in the context of the native enzyme with **(E)-1** bound.

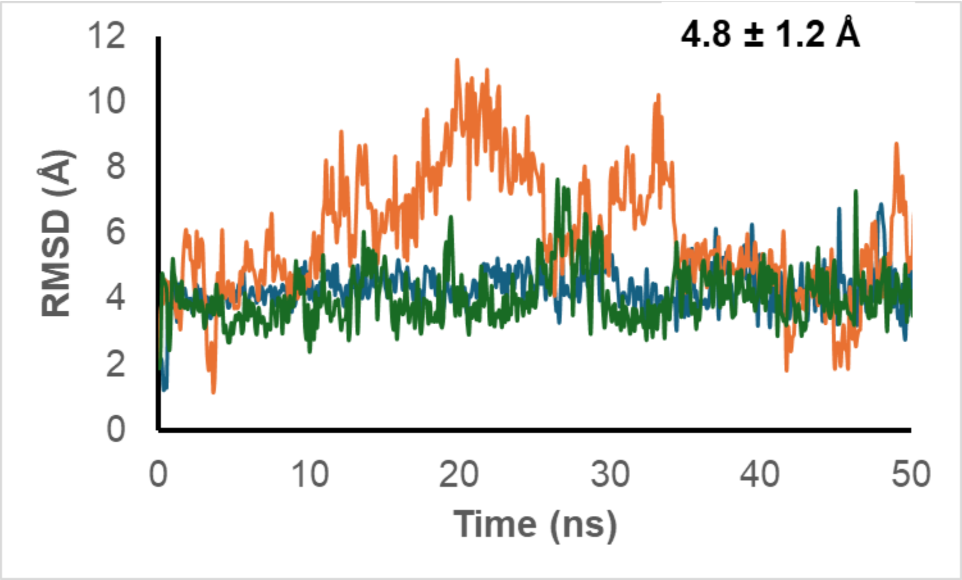

Figure S12. RMSD analysis of the active site Br^- ion over 50 ns MD simulations in the context of the native enzyme with **(Z)-1** bound.

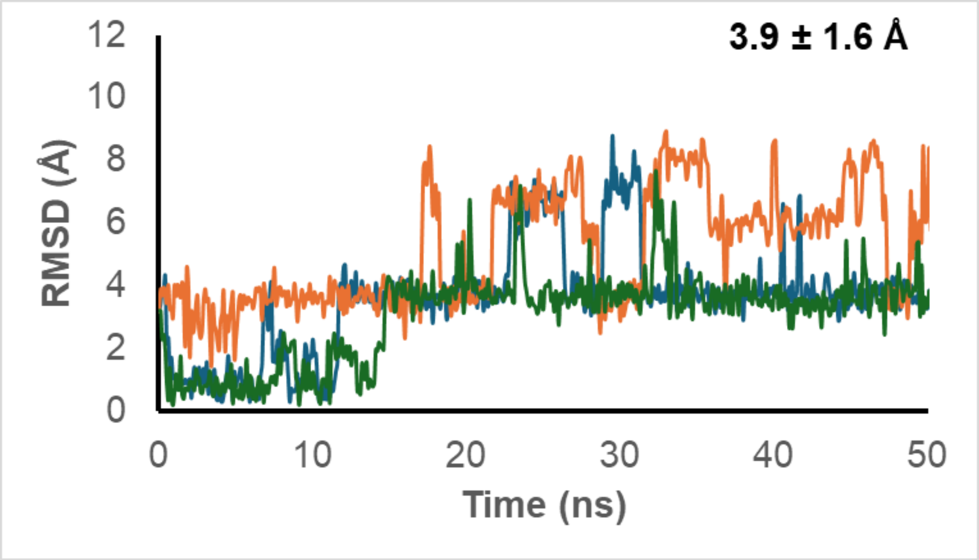

Figure S13. RMSD analysis of the active site Br- ion over 50 ns MD simulations in the context of the D335G enzyme with **(E)-1** bound.

Table S1. MCPB.py derived parameters for deprotonated orthovanadate center.

[atomtypes]												
name	bond_type	mass	charge	ptype	sigma	epsilon		Amb				
Y1	Y1	14.01	0	A	3.25E-01	7.11E-01	;	1.82	0.17			
M1	M1	50.94	0	A	2.59E-01	1.12E-01	;	1.46	0.0269			
Y2	Y2	16	0	A	2.96E-01	8.79E-01	;	1.66	0.21			
Y3	Y3	16	0	A	2.96E-01	8.79E-01	;	1.66	0.21			
Y4	Y4	16	0	A	2.96E-01	8.79E-01	;	1.66	0.21			
Y5	Y5	16	0	A	2.96E-01	8.79E-01	;	1.66	0.21			
[atoms]												
nr	type	resi	res	atom	cgnr	charge	mass	;	qtot	bond_type		
9106	M1	598	V1	V	9106	1.191488	50.94	;	qtot	-31.746		
9107	HO	599	O11	H	9107	0.348646	1.008	;	qtot	-31.397		
9108	Y2	599	O11	O	9108	-0.77184	16	;	qtot	-32.169		
9109	HO	600	O21	H	9109	0.224167	1.008	;	qtot	-31.945		
9110	Y3	600	O21	O	9110	-0.6956	16	;	qtot	-32.64		
9111	Y4	601	O31	O	9111	-0.74992	16	;	qtot	-33.39		
9112	Y5	602	O41	O	9112	-0.60994	16	;	qtot	-34		
[bonds]												
;	ai	aj	funct	r	k							
	8388	9106	1	2.39E-01	8.95E+03	;	NE2	-	V			
	9106	9108	1	1.87E-01	1.51E+05	;	V	-	O			
	9106	9110	1	1.90E-01	1.36E+05	;	V	-	O			
	9106	9111	1	1.65E-01	4.11E+05	;	V	-	O			
	9106	9112	1	1.63E-01	4.41E+05	;	V	-	O			
	9107	9108	1	9.81E-02	2.99E+05	;	H	-	O			
	9109	9110	1	9.81E-02	2.99E+05	;	H	-	O			
[angles]												
;	ai	aj	ak	funct	theta	cth						
	8386	8388	9106	1	1.21E+02	7.83E+02	;	CE1	-	NE2		
	8388	9106	9108	1	1.66E+02	5.33E+02	;	NE2	-	V		
	8388	9106	9110	1	7.45E+01	5.95E+02	;	NE2	-	V		
	8388	9106	9111	1	8.17E+01	5.32E+02	;	NE2	-	V		
	8388	9106	9112	1	8.64E+01	5.16E+02	;	NE2	-	V		
	8389	8388	9106	1	1.32E+02	8.29E+02	;	CD2	-	NE2		
	9106	9108	9107	1	1.04E+02	2.35E+02	;	V	-	O		
	9106	9110	9109	1	1.03E+02	2.29E+02	;	V	-	O		
	9108	9106	9110	1	9.19E+01	7.10E+02	;	O	-	V		
	9108	9106	9111	1	1.03E+02	8.06E+02	;	O	-	V		
	9108	9106	9112	1	1.04E+02	7.18E+02	;	O	-	V		
	9110	9106	9111	1	1.22E+02	1.03E+03	;	O	-	V		
	9110	9106	9112	1	1.17E+02	9.23E+02	;	O	-	V		
	9111	9106	9112	1	1.13E+02	9.60E+02	;	O	-	V		
[dihedrals] ; propers												
; for gromacs 4.5 or higher, using funct 9												
	i	j	k	l	func	phase	kd	pn				
	9107	9108	9106	9110	9	0	0	0	H-	O-	V-	O
	9107	9108	9106	9111	9	0	0	0	H-	O-	V-	O
	9107	9108	9106	9112	9	0	0	0	H-	O-	V-	O
	9108	9106	9110	9109	9	0	0	0	O-	V-	O-	H
	9109	9110	9106	9111	9	0	0	0	H-	O-	V-	O
	9109	9110	9106	9112	9	0	0	0	H-	O-	V-	O

Microscale Thermophoresis Experiments

Binding Experiments using MST: Wild-type *Cp*VBPO MST and the D335G mutant were labeled with RED NHS dye (Cat #MO-L011, NanoTemper Technologies) in the provided labeling buffer. Subsequently, the proteins were buffer exchanged into 100 mM citrate, pH 6. The labeling ratio of dye to protein was determined spectroscopically following the instrument manufacturer's guidelines.

Aliquots of 1.2 μM labeled protein solution were stored at –80 °C. Aliquots were diluted to 10-25 nM before use. Sodium vanadate and Tween-80 were added to aliquots to achieve concentrations of 0.6 mM, 90 mM, and 0.05% respectively.

Binding experiments were performed using 10-25 nM protein and 162-600 mM hydrazone (E and Z) solutions. A series of 16 dilutions (1:2) of the hydrazone were prepared and mixed with the labeled protein. Hydrazone E and Z were dissolved in DMSO to afford stock concentrations of 810 mM and 600 mM. To ensure all samples contained a constant DMSO concentration throughout the dilution series, 37% or 50 % DMSO was added to the buffer for serial dilutions. The samples were incubated for 1-3 hours prior to running measurements. All measurements were carried out on a Monolith NT.115 Pico (NanoTemper Technologies) at 34 °C. Assays were conducted at 5-20% IR-laser power and medium MST power. The fluorescence change was plotted against the concentration of the hydrazone and curves were fitted with K_d model in the MO. Affinity Analysis Software (NanoTemper Technologies). The buffer used for the binding assays was 100 mM citrate pH 6, 0.05% Tween.

Summary of Results: Using microscale thermophoresis (MST), we assessed the binding affinity of wild-type and D335G mutant *Cp*VBPO for both hydrazone isomers. For wild-type *Cp*VBPO, the binding constant with hydrazone E was estimated to be ~33 mM. Critically, the lack of a well-defined plateau at higher ligand concentrations indicates that a higher maximum ligand concentration is required. However, solubility limitations of the ligand prevented this. Multiple solvents (DMF, MeOH, MeCN) were tested to improve solubility, but DMSO proved most effective and was therefore used for all experiments with calculated K_d values. Under these conditions, *Cp*VBPO bound hydrazone Z with a K_d of ~2.4 mM, indicating that hydrazone Z associates much more strongly than hydrazone E.

The D335G mutant was also evaluated for binding with both hydrazone isomers. The K_d values were ~35 mM for hydrazone E and ~174 mM for hydrazone Z, suggesting that, in contrast to the wild type, hydrazone E binds more strongly than hydrazone Z.

Table S2. Summary of K_d Measurements for *Cp*VBPO with Hydrazone E by MST*

Concentration of WT *Cp*VBPO (nM)	Maximum Concentration of E-hydrazone (mM)	K_D (mM)
25	81	35.7
25	300	32.2
25	300	30.4
		**Average: 32.8 ± 2.7**

*time 1.5 sec

Table S3. Summary of K_d Measurements for *Cp*VBPO with Hydrazone Z by MST*

Concentration of WT *Cp*VBPO (nM)	Maximum Concentration of E-hydrazone (mM)	K_D (mM)
25	81	2.6
25	300	2.1
10	200	2.4
		**Average: 2.4 ± 0.3**

*time 1.5 sec

Table S4. Summary of K_d Measurements for D335G *Cp*VBPO with Hydrazone E by MST*

Concentration of WT *Cp*VBPO (nM)	Maximum Concentration of E-hydrazone (mM)	K_D (mM)
25	81	44.1
25	300	38.5
25	300	22.6
		**Average: 35.1 ± 11.2**

*time 1.5 sec

Table S5. Summary of K_d Measurements for D335G *Cp*VBPO with Hydrazone Z by MST*

Concentration of WT *Cp*VBPO (nM)	Maximum Concentration of E-hydrazone (mM)	K_D (mM)
25	300	79.1
25	300	184.1
25	300	258.9
		**Average: 174.0 ± 90.3**

*time 15 sec

References:

1. Wells, C. E.; Ramos, L. P. T.; Harstad, L. J.; Hessefort, L. Z.; Lee, H. J.; Sharma, M.; Biegasiewicz, K. F. Decarboxylative Bromooxidation of Indoles by a Vanadium Haloperoxidase. *ACS Catal.* **2023**, *13*, 4622-4628.
2. Sharma, M.; Pascoe, C. A.; Jones, S. K.; Barthel, S. G.; Davis, K. M.; Biegasiewicz, K. F. Intermolecular 1,2,4-Thiadiazole Synthesis Enabled by Enzymatic Halide Recycling with Vanadium-Dependent Haloperoxidases. *J. Am. Chem. Soc.* **2025**, *147*, 10698–10705.
3. Nicolle, S. M.; Moody, C. J. Potassium *N* -Iodo *p* -Toluenesulfonamide (TsNIK, Iodamine-T): A New Reagent for the Oxidation of Hydrazones to Diazo Compounds. *Chem. Eur. J.* **2014**, *20* (15), 4420–4425.
4. Dhara, K.; Mandal, T.; Das, J.; Dash, J. Synthesis of Carbazole Alkaloids by Ring-Closing Metathesis and Ring Rearrangement–Aromatization. *Angew. Chem. Int. Ed.* **2015**, *54* (52), 15831–15835.
5. Fan, Y.-S.; Jiang, Y.-J.; An, D.; Sha, D.; Antilla, J. C. Zhang, S. H_8-BINOL Chiral Imidodiphosphoric Acids Catalyzed Enantioselective Synthesis of Dihydroindolo-/-pyrrolo[1,2- *a*]quinoxalines. *Org. Lett.* **2014**, 16, 6112–6115.
6. Liu, W.; Twilton, J.; Wei, B.; Lee, M.; Hopkins, M. N.; Bacsa, J.; Stahl, S. S.; Davies, H.M.L. Copper-Catalyzed Oxidation of Hydrazones to Diazo Compounds Using Oxygen as the Terminal Oxidant. *ACS Catal.* **2021**, *11* (5), 2676–2683.
7. Kumar, N.; Venkatesh, R.; Kandasamy, J. Synthesis of functionalized *S* -benzyl dithiocarbamates from diazo-compounds *via* multi-component reactions with carbon disulfide and secondary amines. *Org. Biomol. Chem.* **2022**, *20* (34), 6766–6770.
8. Tanbouza, N.; Caron, L.; Khoshoei, A.; Ollevier, T. Catalytic Bismuth(V)-Mediated Oxidation of Hydrazones into Diazo Compounds. *Org. Lett.* **2022**, *24* (14), 2675–2678.
9. Ye, F.; Qu, S.; Zhou, L.; Peng, C.; Wang, C.; Cheng, J.; Hossain, M. L.; Liu, Y.; Zhang, Y.; Wang, Z.-X.; Wang, J. Palladium-Catalyzed C–H Functionalization of Acyldiazomethane and Tandem Cross-Coupling Reactions. *J. Am. Chem. Soc.* **2015**, *137* (13), 4435–4444.
10. Lefebvre, Q.; Fava, E.; Nikolaienko, P.; Rueping. M. Hydrotrifluoromethylthiolation of α-diazo esters – synthesis of α-SCF3 substituted esters. *Chem. Commun.* **2014**, *50* (50), 6617.
11. Ma, B.; Wu, P.; Wang, X.; Wang, Z.; Lin, H.; Dai. H. Efficient Synthesis of Spirooxindole Pyrrolones by a Rhodium(III)-Catalyzed C−H Activation/Carbene Insertion/Lossen Rearrangement Sequence. *Angew. Chem. Int. Ed.* **2019**, *58* (38), 13335–13339.
12. Liu, J.; Mallick, S.; Xie, Y.; Grassin, C.; Lucas, B.; Schölermann, B.; Pahl, A.; Scheel, R.; Strohmann, C.; Protzel, C.; Berg, T.; Merten, C.; Ziegler, S.; Waldmann, H. Morphological Profiling Identifies the Motor Protein Eg5 as Cellular Target of Spirooxindoles. *Angew. Chem. Int. Ed.* **2023**, *62*, e202301955.
13. Yao, X.; Wang, T.; Zhang, Z. Gold(I)-Catalyzed Dimerization of 3-Diazooxindoles towards Isoindigos. *Eur. J. Org. Chem.* **2018**, *2018* (32), 4475–4478.

14. Sasane, A. V.; Kuo, T.-C.; Cheng, M.-J.; Liu, R.-S. Gold-Catalyzed Rearrangement of α-Carbonyl Cyclopropanes to Form 3-(Cyclopenta-1,3-dien-1-ylmethyl)oxindoles via a Postulated 1,5-Enolate Shift. *Org. Lett***. 2022**, *24* (28), 5220–5225.
15. Tanimoto, H.; Adachi, R.; Tanisawa, K.; Tomohiro, T. Amphos-Mediated Conversion of Alkyl Azides to Diazo Compounds and One-Pot Azide-Site Selective Transient Protection, Click Conjugation, and Deprotective Transformation. *Org. Lett.* **2024**, *26*, 2409–2413.
16. Xu, W.; Yamakawa, T.; Huang, M.; Tian, P.; Jiang, Z.; Xu, M. Conformational Locking Induced Enantioselective Diarylcarbene Insertion into B−H and O−H Bonds Using a Cationic Rh(I)/Diene Catalyst. *Angew. Chem. Int. Ed*. **2024**, *63,* No. e202412193.
17. Bugnon, M.; Goullieux, M.; Röhrig, U. T.; Perez, M. A. S.; Daina, A.; Michielin, O.; Zoete, V. SwissParam 2023: A Modern Web-Based Tool for Efficient Small Molecule Parameterization. *J. Chem. Inf. and Model.* **2023,** *63* (21), 6469-6475.
18. Mitchell, D. E.; Garcia-Rodriguez, E.; Isupov, M. N.; Littlechild, J. A. Insights into the Mechanism of a Vanadium Bromoperoxidase from the Marine Macro-Algae *Corallina pilulifera* for Biocatalytic Halogenation. *ChemCatChem*, 2025, *17*(16), e202400792.
19. Goodsell, D. S.; Olson, A. J. Automated docking of substrates to proteins by simulated annealing. *Proteins:Struct., Funct., Bioinf.* **1990**, *8*, 195–202.
20. Goodsell, D. S.; Sanner, M. F.; Olson, A. J.; Forli, S. The AutoDock suite at 30. *Protein Sci.* **2021**, *30*, 31–43.
21. Forli, S.; Huey, R.; Pique, M. E.; Sanner, M. F.; Goodsell, D. S.; Olson, A. J. Computational protein–ligand docking and virtual drug screening with the AutoDock suite. *Nat. Protoc.* **2016**, *11*, 905–919.
22. Morris, G. M.; Huey, R.; Lindstrom, W.; Sanner, M. F.; Belew, R. K.; Goodsell, D. S.; Olson, A. J. AutoDock4 and AutoDockTools4: Automated Docking with Selective Receptor Flexibility. *J. Comput. Chem.* **2009**, *30,* 2785-2791.
23. DeLano, W. L., *CCP4 Newsl. Protein Crystallogr* **2002,** *40*, 82-92.
24. Lindorff-Larsen, K.; Piana, S.; Palmo, K.; Maragakis, P.; Klepeis, J. L.; Dror, R. O.; Shaw, D. E. Improved side-chain torsion potentials for the Amber ff99SB protein force field. *Proteins: Proteins:Struct., Funct., Bioinf.* **2010**, *78*, 1950–1958.
25. GROMACS user manual version 2019.3. Abraham, M. J.; van der Spoel, D.; Lindahl, E.; Hess, B. and The Gromacs Development Team.
26. van der Spoel, D.; Lindahl, E.; Hess, B.; Groenhof, G.; Mark, A. E.; Berendsen, H. J. C. GROMACS: Fast, flexible, and free. *J. Comput. Chem.* **2005**, *26*, 1701–1718.
27. Berendsen, H. J. C.; van der Spoel, D.; van Drunen, R. GROMACS: A message-passing parallel molecular dynamics implementation. *Comput. Phys. Commun.***1995**, *91*, 43–56.
28. Mark, P.; Nilsson, L. Structure and Dynamics of the TIP3P, SPC, and SPC/E Water Models at 298 K. *J. Phys. Chem. A.* **2001**, *105*, 9954–9960.
29. Berendsen, H. J. C.; Grigera, J. R.; Straatsma, T. P. The missing term in effective pair potentials. *J. Phys. Chem.* **1987**, *91*, 6269–6271.
30. Bussi, G.; Donadio, D.; Parrinello, M. Canonical sampling through velocity rescaling. *J. Chem. Phys.* **2007**, *126*, e1.2408420.
31. Hess, B. P-LINCS: A Parallel Linear Constraint Solver for Molecular Simulation. *J. Chem. Theory Comput.* **2008**, *4*, 116–122.

32. Miyamoto, S.; Kollman, P. A. Settle: An analytical version of the SHAKE and RATTLE algorithm for rigid water models. *J. Comput. Chem.***1992**, *13*, 952–962.
33. Essmann, U.; Perera, L.; Berkowitz, M. L.; Darden, T.; Lee, H.; Pedersen, L. G. A smooth particle mesh Ewald method. *J. Chem. Phys.* **1995**, *103*, 8577–8593.
34. Berendsen, H. J. C.; Postma, J. P. M.; van Gunsteren, W. F.; DiNola, A.; Haak, J. R. Molecular dynamics with coupling to an external bath. *J. Chem. Phys.* **1984**, *81*, 3684–3690.
35. Parrinello, M.; Rahman, A. Polymorphic transitions in single crystals: A new molecular dynamics method. *J. Appl. Phys.* **1981**, *52*, 7182–7190.
36. Li, P.; Merz, K. M. MCPB.py: A Python Based Metal Center Parameter Builder. *J. Chem. Inf. Model.* **2016**, *56*, 599–604.
37. Gaussian 16, Revision C.01, Frisch, M. J.; Trucks, G. W.; Schlegel, H. B.; Scuseria, G. E.; Robb, M. A.; Cheeseman, J. R.; Scalmani, G.; Barone, V.; Petersson, G. A.; Nakatsuji, H.; Li, X.; Caricato, M.; Marenich, A. V.; Bloino, J.; Janesko, B. G.; Gomperts, R.; Mennucci, B.; Hratchian, H. P.; Ortiz, J. V.; Izmaylov, A. F.; Sonnenberg, J. L.; Williams-Young, D.; Ding, F.; Lipparini, F.; Egidi, F.; Goings, J.; Peng, B.; Petrone, A.; Henderson, T.; Ranasinghe, D.; Zakrzewski, V. G.; Gao, J.; Rega, N.; Zheng, G.; Liang, W.; Hada, M.; Ehara, M.; Toyota, K.; Fukuda, R.; Hasegawa, J.; Ishida, M.; Nakajima, T.; Honda, Y.; Kitao, O.; Nakai, H.; Vreven, T.; Throssell, K.; Montgomery, J. A., Jr.; Peralta, J. E.; Ogliaro, F.; Bearpark, M. J.; Heyd, J. J.; Brothers, E. N.; Kudin, K. N.; Staroverov, V. N.; Keith, T. A.; Kobayashi, R.; Normand, J.; Raghavachari, K.; Rendell, A. P.; Burant, J. C.; Iyengar, S. S.; Tomasi, J.; Cossi, M.; Millam, J. M.; Klene, M.; Adamo, C.; Cammi, R.; Ochterski, J. W.; Martin, R. L.; Morokuma, K.; Farkas, O.; Foresman, J. B.; Fox, D. J. Gaussian, Inc., Wallingford CT, **2016**.
38. Lee, C.; Yang, W.; Parr, R. G. Development of the Colle-Salvetti correlation-energy formula into a functional of the electron density. *Phys. Rev. B* **1988**, *37*, 785–789.
39. Becke, A. D. Density-functional thermochemistry. III. The role of exact exchange. *J. Chem. Phys.* **1993**, *98,* 5648–5652.
40. Becke, A. D. Density-functional exchange-energy approximation with correct asymptotic behavior. *Phys. Rev. A* **1988**, *38*, 3098–3100.
41. Hay, P. J.; Wadt, W. R. *Ab initio* effective core potentials for molecular calculations. Potentials for K to Au including the outermost core orbitals. *J. Chem. Phys.* **1985**, *82*, 299–310.
42. Hay, P. J.; Wadt, W. R. *Ab initio* effective core potentials for molecular calculations. Potentials for the transition metal atoms Sc to Hg. *J. Chem. Phys.* **1985**, *82*, 270–283.
43. Krishnan, R.; Binkley, J. S.; Seeger, R.; Pople, J. A. Self-consistent molecular orbital methods. XX. A basis set for correlated wave functions. *J. Chem. Phys.* **1980**, *72*, 650–654.
44. Seminario, J. M. Calculation of intramolecular force fields from second-derivative tensors. *Int. J. Quantum Chem.* **1996**, *60*, 1271–1277.
45. Bayly, C. I.; Cieplak, P.; Cornell, W.; Kollman, P. A. A well-behaved electrostatic potential based method using charge restraints for deriving atomic charges: the RESP model. *J. Phys. Chem.* **1993**, *97*, 10269–10280.
46. Singh, U. C.; Kollman, P. A. An approach to computing electrostatic charges for molecules. *J. Comput. Chem.* **1984**, *5*, 129–145.

47. Besler, B. H.; Merz, K. M.; Kollman, P. A. Atomic charges derived from semiempirical methods. *J. Comput. Chem.* **1990**, *11,* 431–439.
48. Mubarak, M. Q. E.; Gérard, E. F.; Blanford, C. F.; Hay, S.; de Visser, S. P. How Do Vanadium Chloroperoxidases Generate Hypochlorite from Hydrogen Peroxide and Chloride? A Computational Study. *ACS Catal.* **2020**, *10*, 14067–14079.
49. Gérard, E. F.; Mokkawes, T.; Johannissen, L. O.; Warwicker, J.; Spiess, R. R.; Blanford, C. F.; Hay, S.; Heyes, D. J.; de Visser, S. P. How Is Substrate Halogenation Triggered by the Vanadium Haloperoxidase from *Curvularia inaequalis* ? *ACS Catal.* **2023***, 13*, 8247–8261.
50. Kravitz, J. Y.; Pecoraro, V. L.; Carlson, H. A. Quantum Mechanics/Molecular Mechanics Calculations of the Vanadium Dependent Chloroperoxidase. *J. Chem. Theory Comput.* **2005**, *1*, 1265–1274.
51. Zampella, G.; Fantucci, P.; Pecoraro, V. L.; de Gioia, L. Insight into the Catalytic Mechanism of Vanadium Haloperoxidases. DFT Investigation of Vanadium Cofactor Reactivity. *Inorg. Chem.* **2006**, *45*, 7133–7143.
52. Zhang, Y.; Gascón, J. A. QM/MM investigation of structure and spectroscopic properties of a vanadium-containing peroxidase. *J. Inorg. Biochem*. **2008**, *102*, 1684–1690.
53. Anderson, G. A.; Behera, R. N.; Gomatam, R. Calculation of higher protonation states and of a new resting state for vanadium chloroperoxidase using QM/MM, with an Atom-in-Molecules analysis. J. *Mol. Graphics Modell*. **2020**, *99*, 107624.
54. Lee, C., Yang, W.; Parr, R. G. Development of the Colle-Salvetti correlation-energy formula into a functional of the electron density. *Phys. Rev. B* **1988**, *37*, 785–789.

Spectroscopic Data

Ethyl (*Z*)-2-hydrazineylidene-2-phenylacetate ((Z)-1)

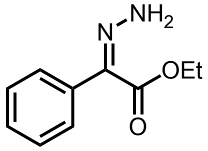

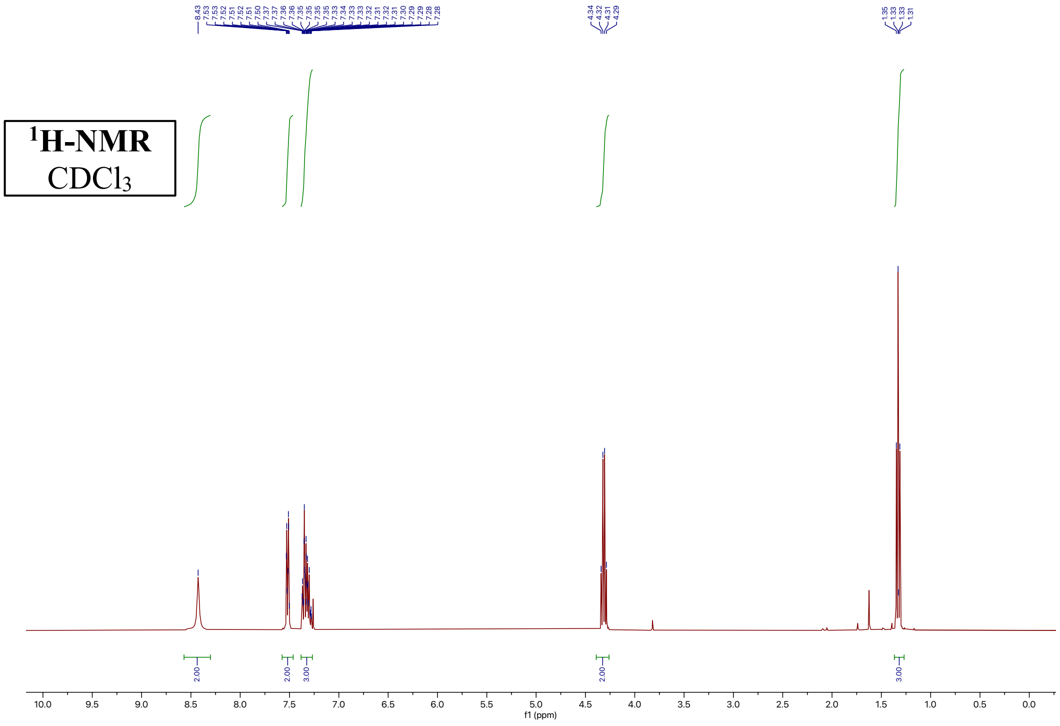

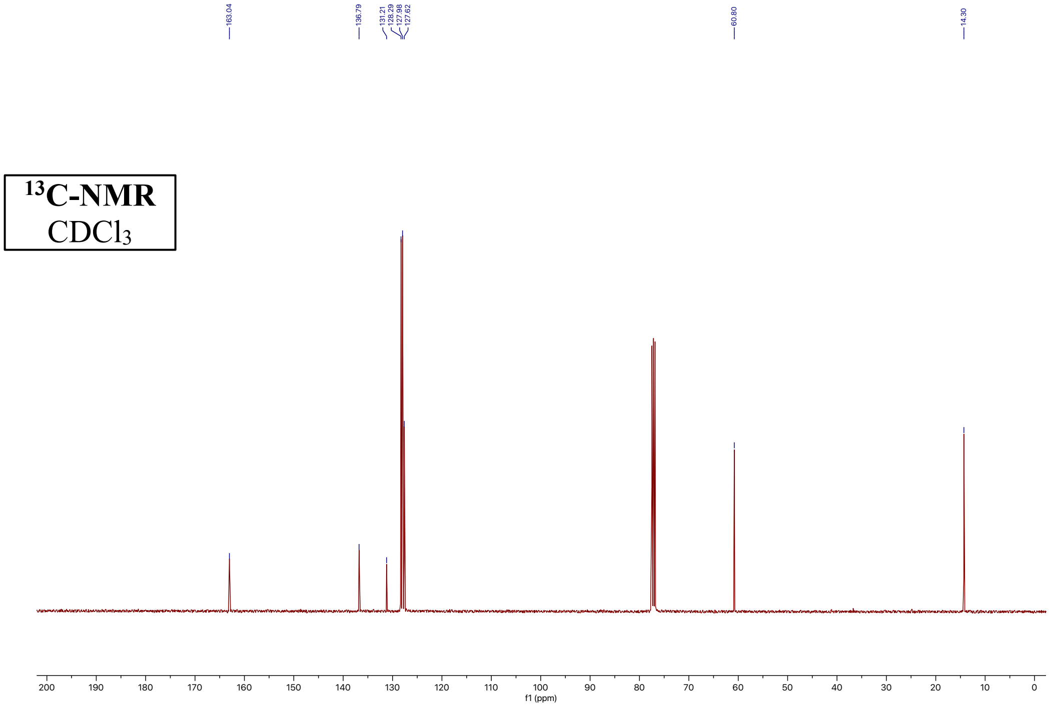

Ethyl (*E*)-2-hydrazineylidene-2-phenylacetate ((E)-1)

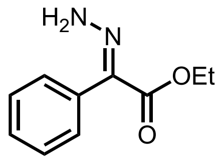

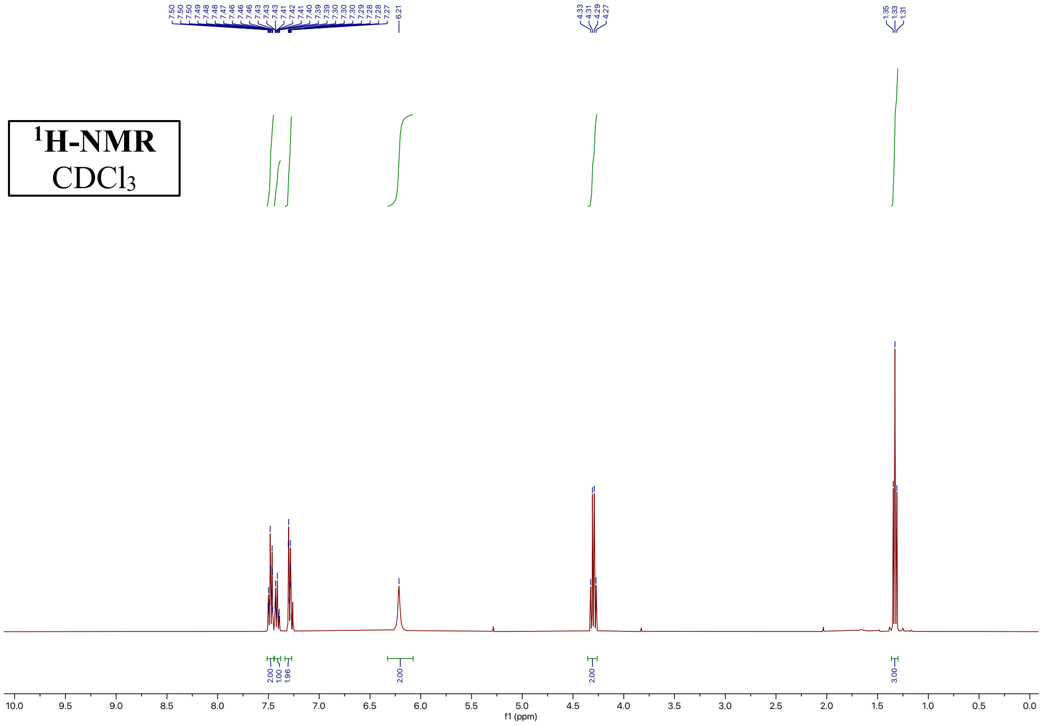

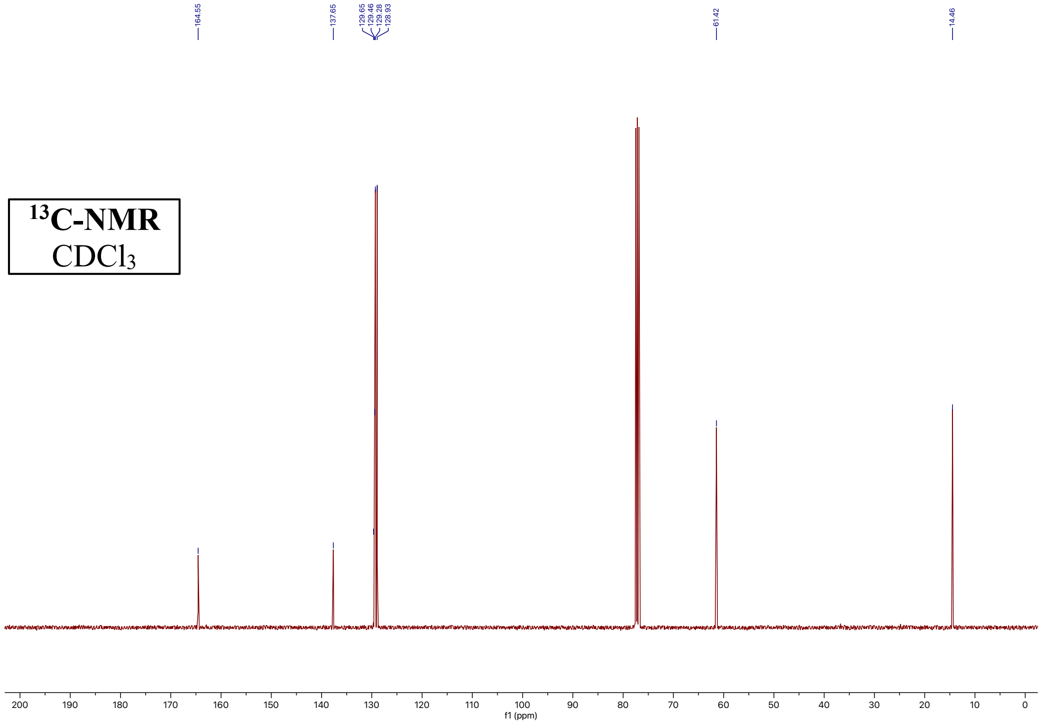

1-Benzyl-5-methylindoline-2,3-dione (SM-1)

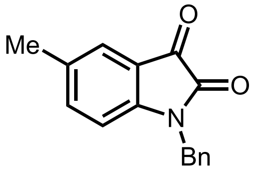

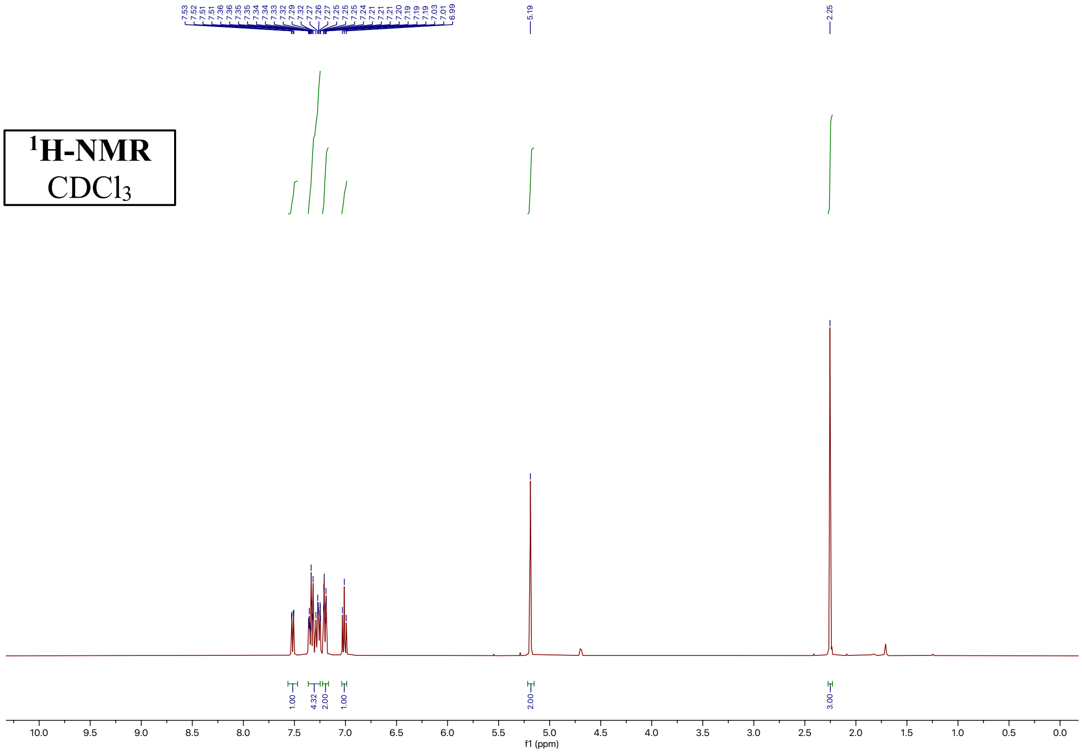

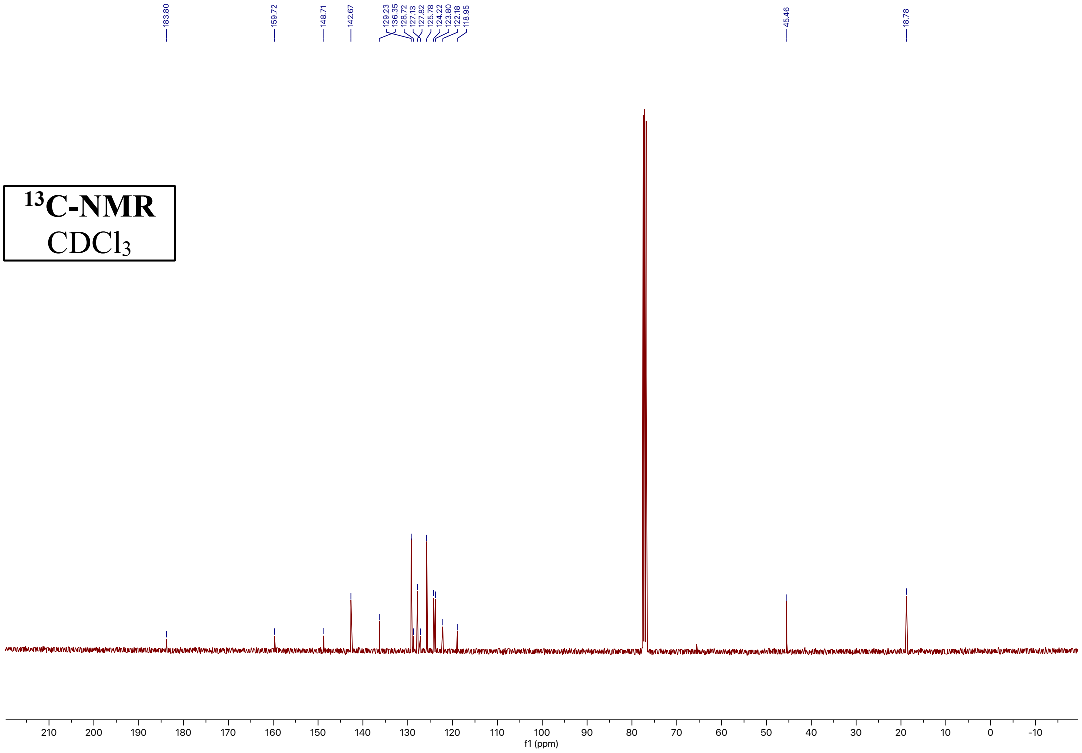

1-Benzyl-5-methoxyindoline-2,3-dione (SM-2)

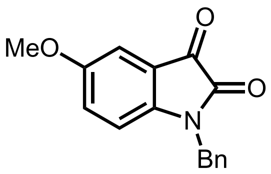

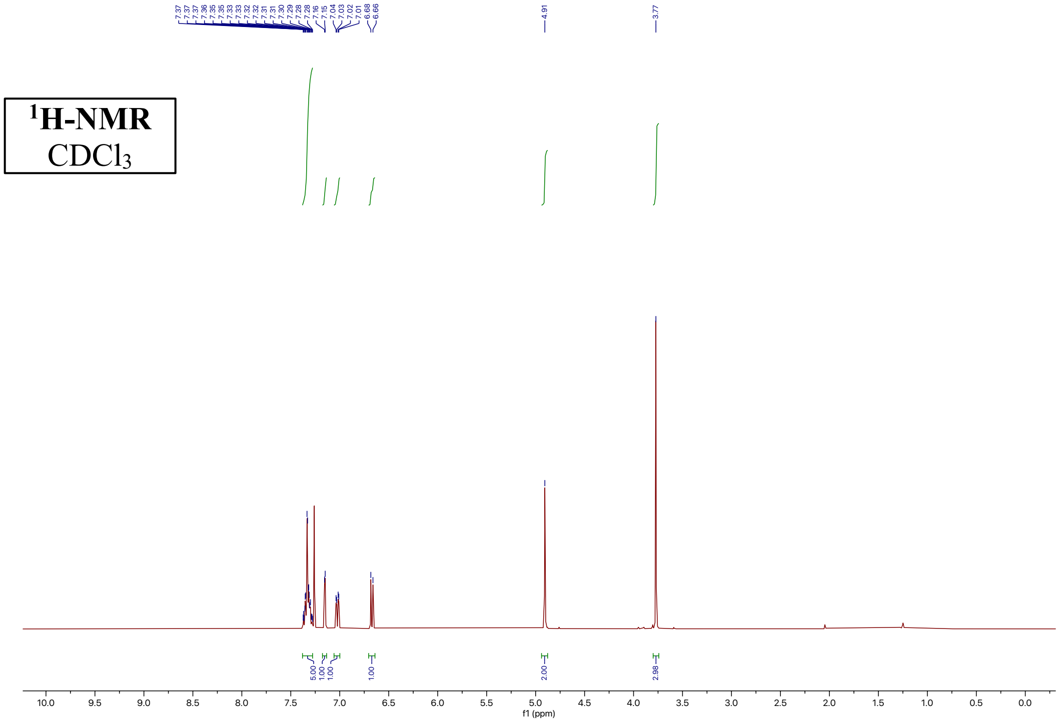

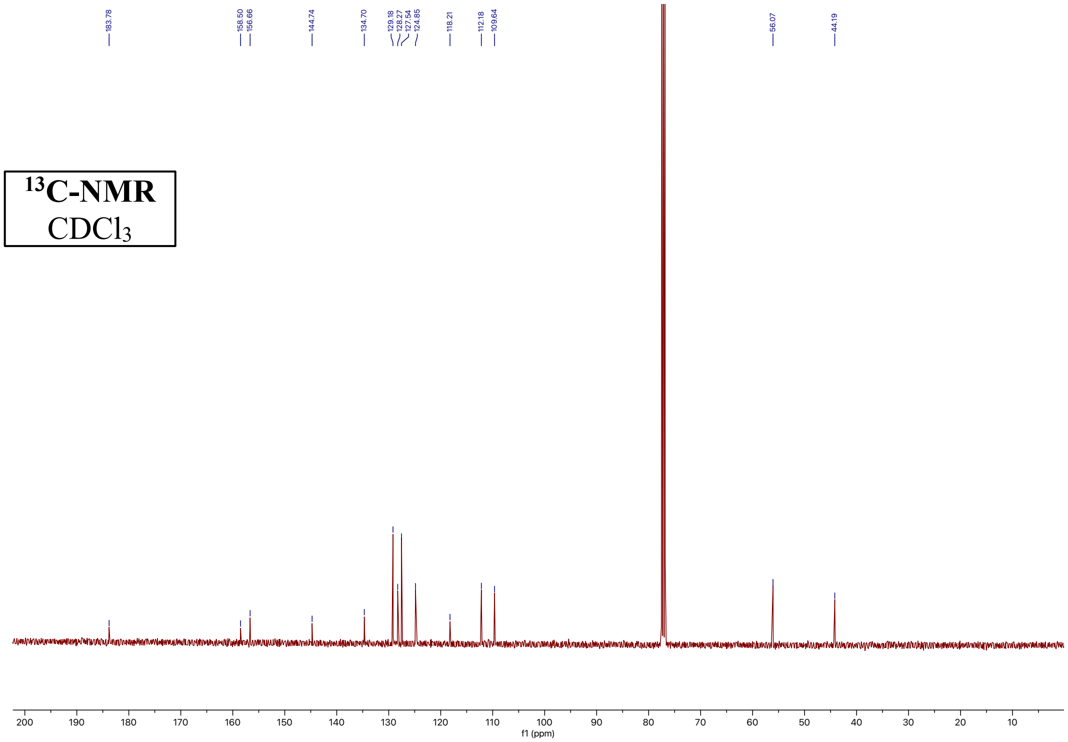

1-Benzyl-7-methylindoline-2,3-dione (SM-3)

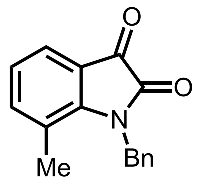

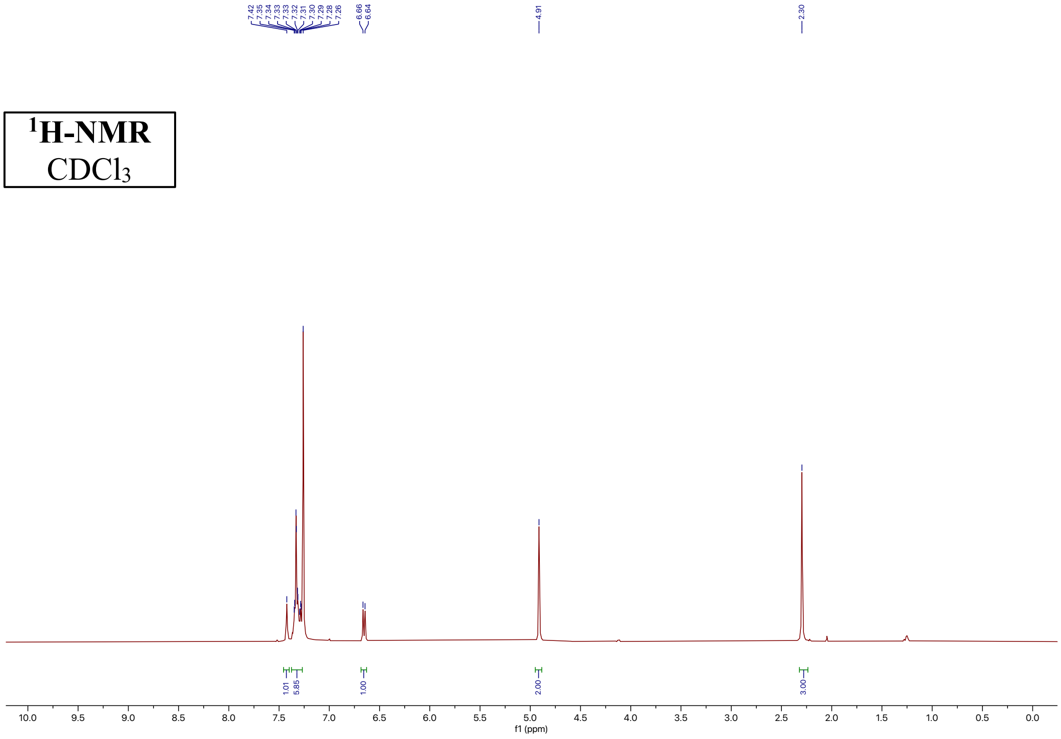

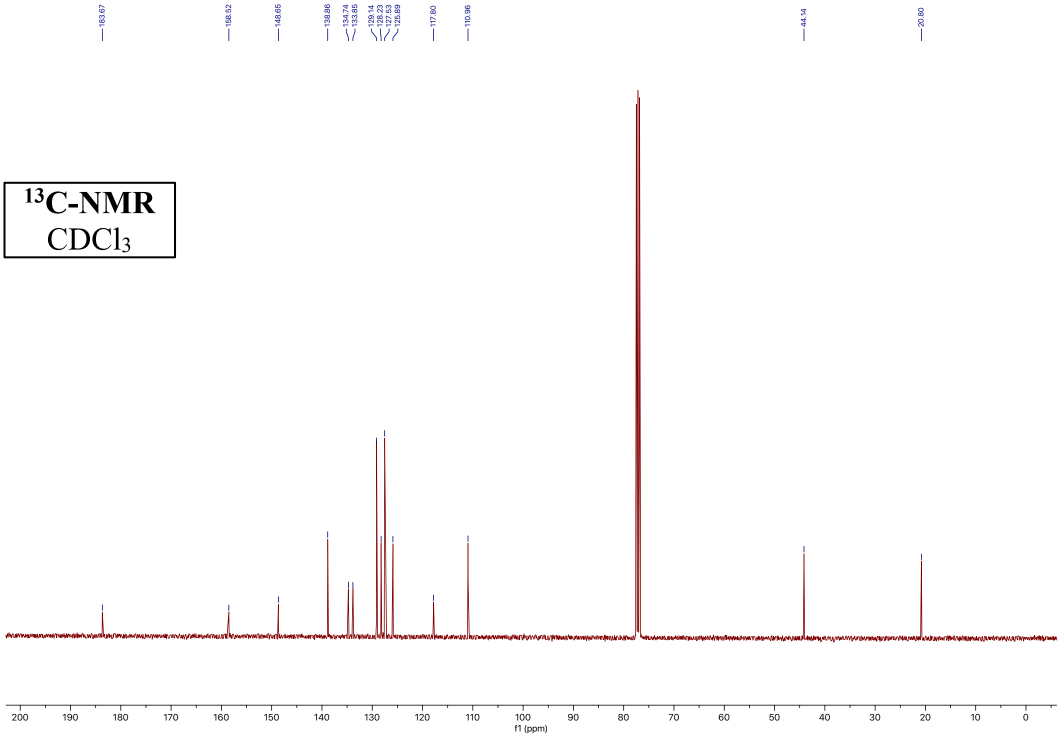

Benzyl 2-oxopropanoate (SM-4)

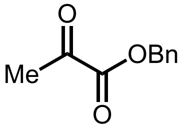

^{1}H-NMR
$CDCl_3$

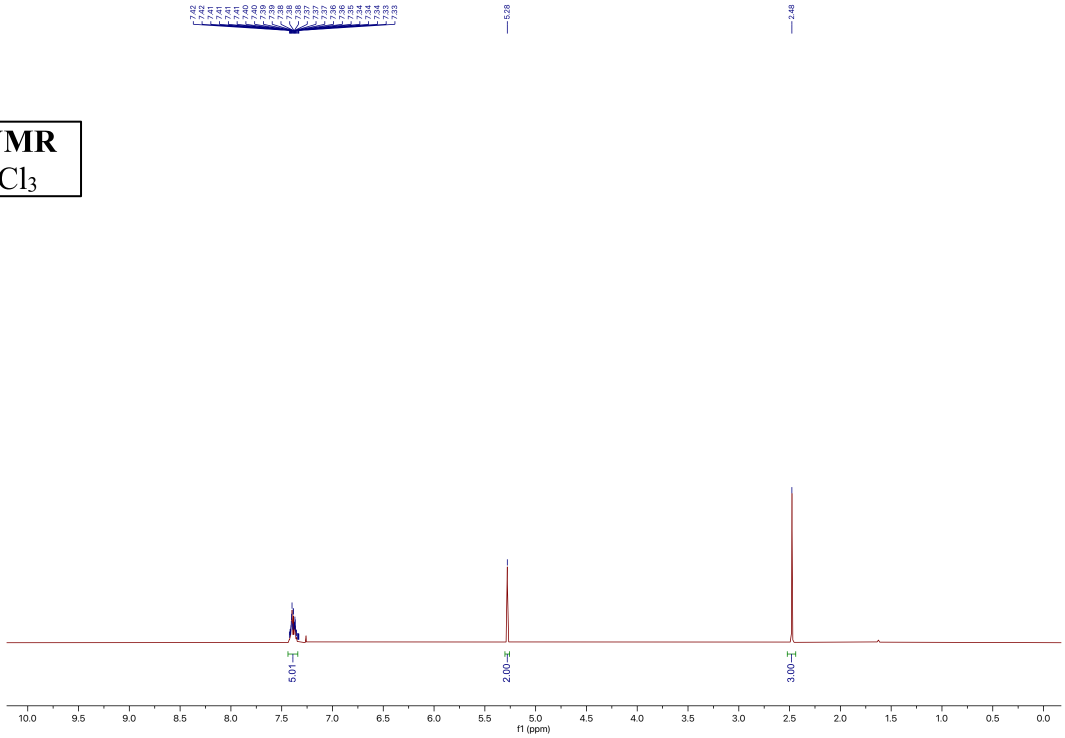

^{13}C-NMR
$CDCl_3$

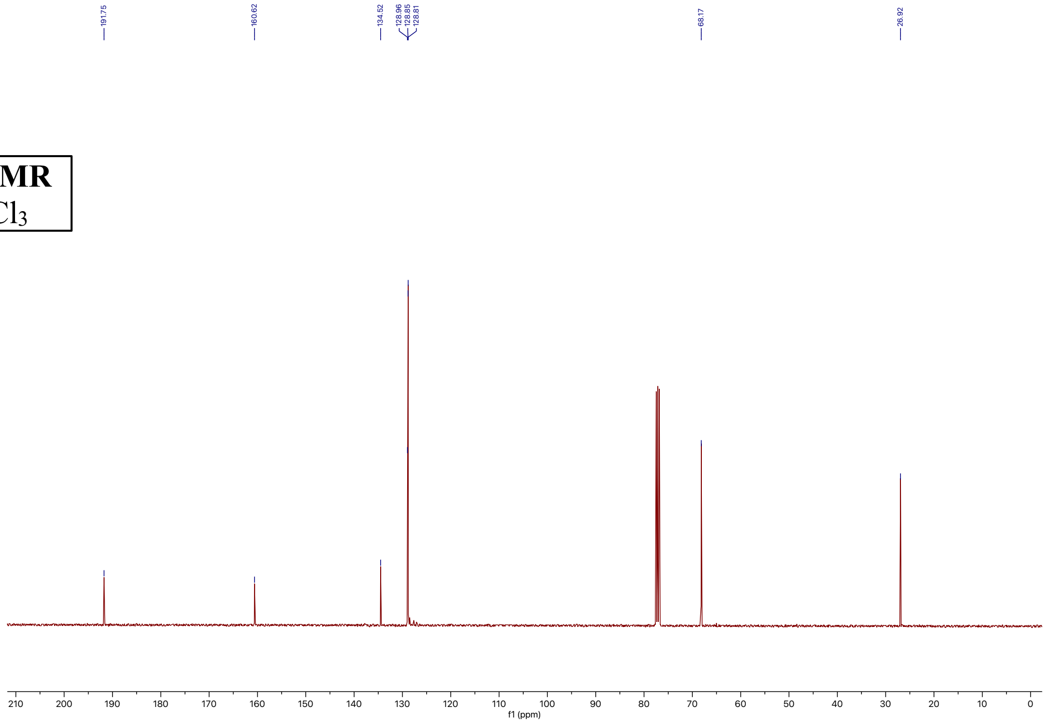

Ethyl 2-diazo-2-phenylacetate (2)

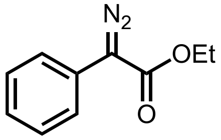

^{1}H-NMR
$CDCl_3$

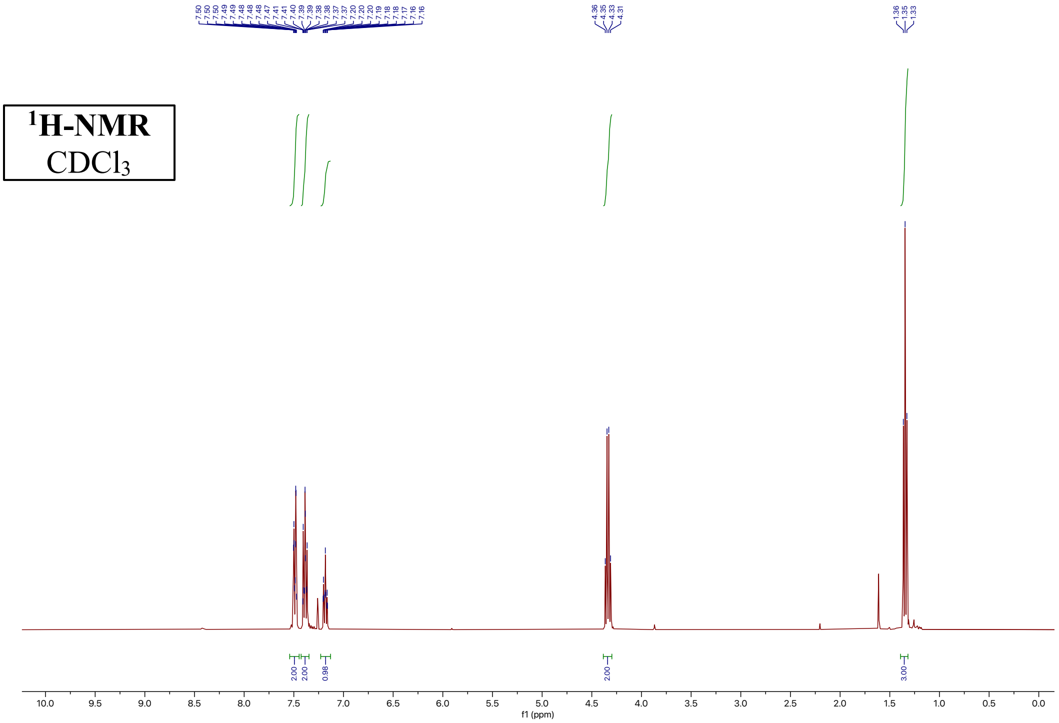

^{13}C-NMR
$CDCl_3$

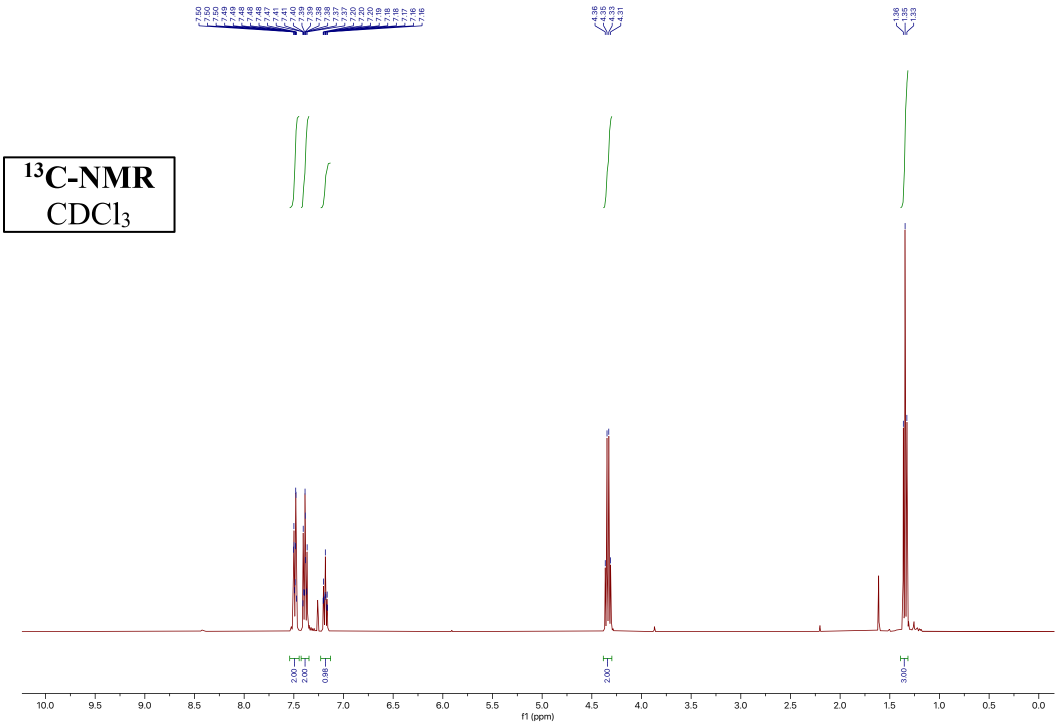

Ethyl 2-diazo-2-(p-tolyl)acetate (3)

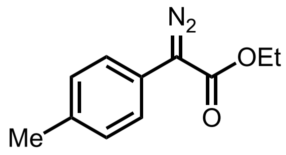

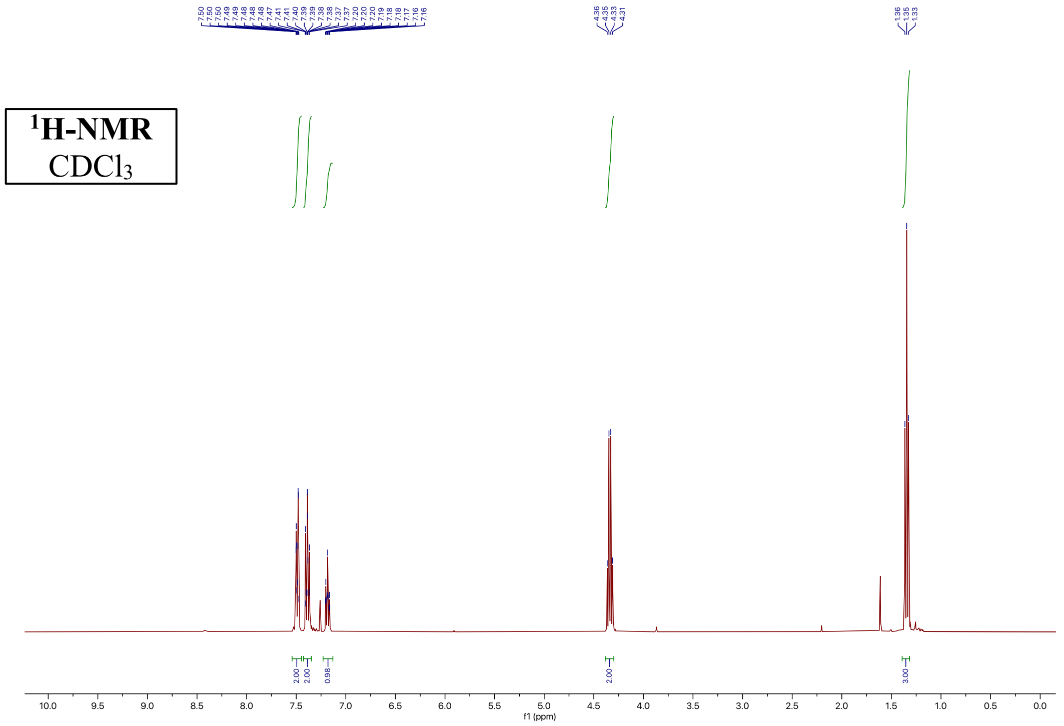

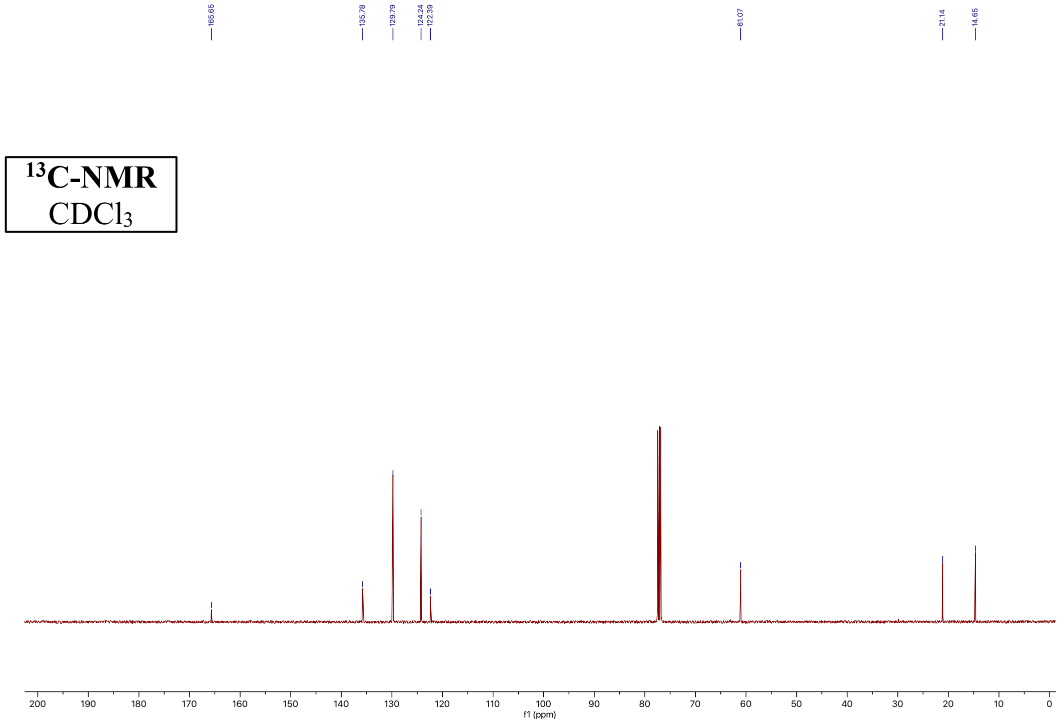

Ethyl 2-(4-(tert-butyl)phenyl)-2-diazoacetate (4)

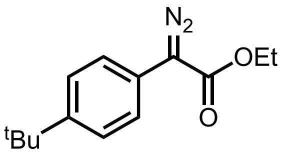

^{1}H-NMR
$CDCl_3$

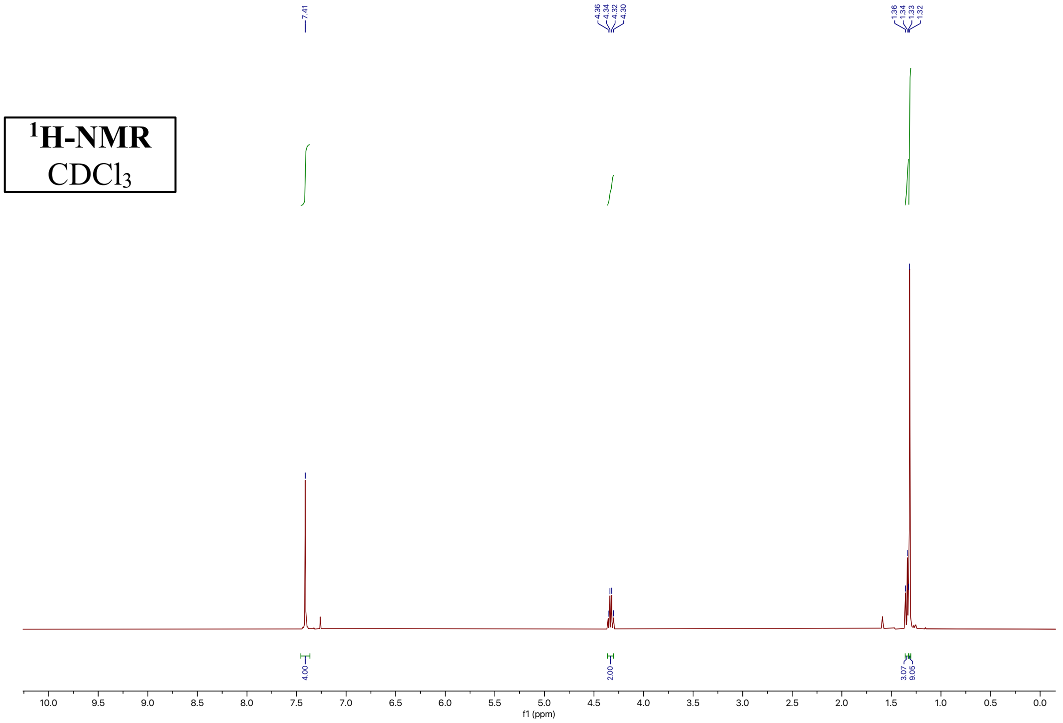

^{13}C-NMR
$CDCl_3$

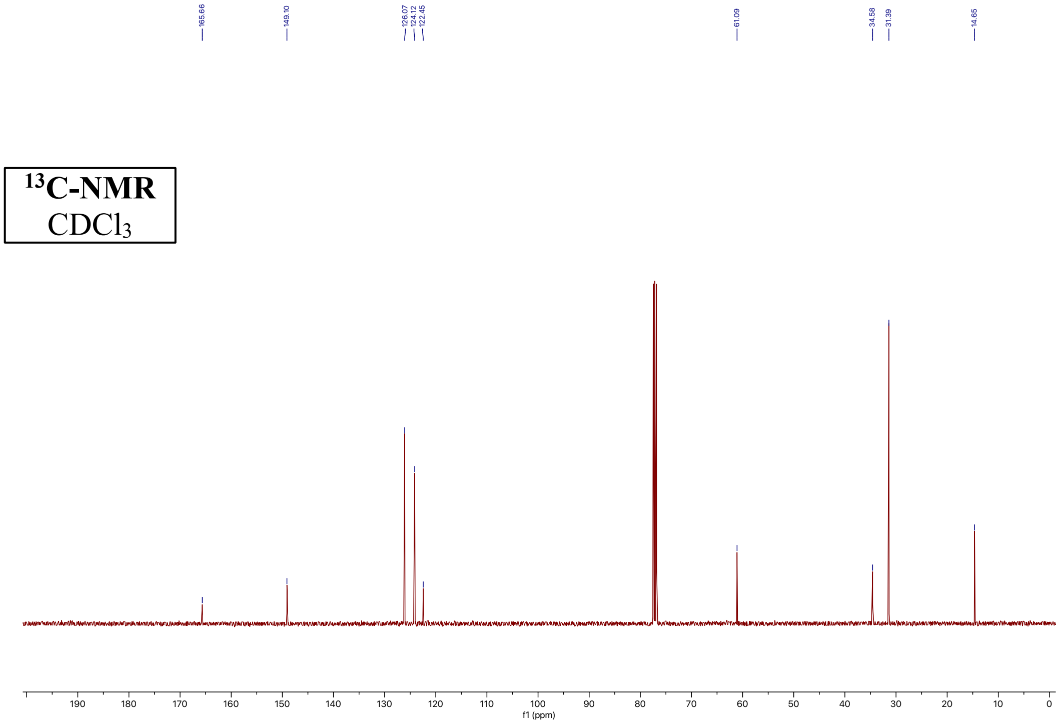

Ethyl 2-diazo-2-(4-methoxyphenyl)acetate (5)

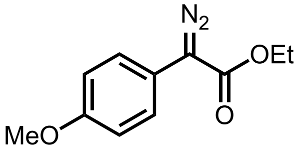

^{1}H-NMR
$CDCl_3$

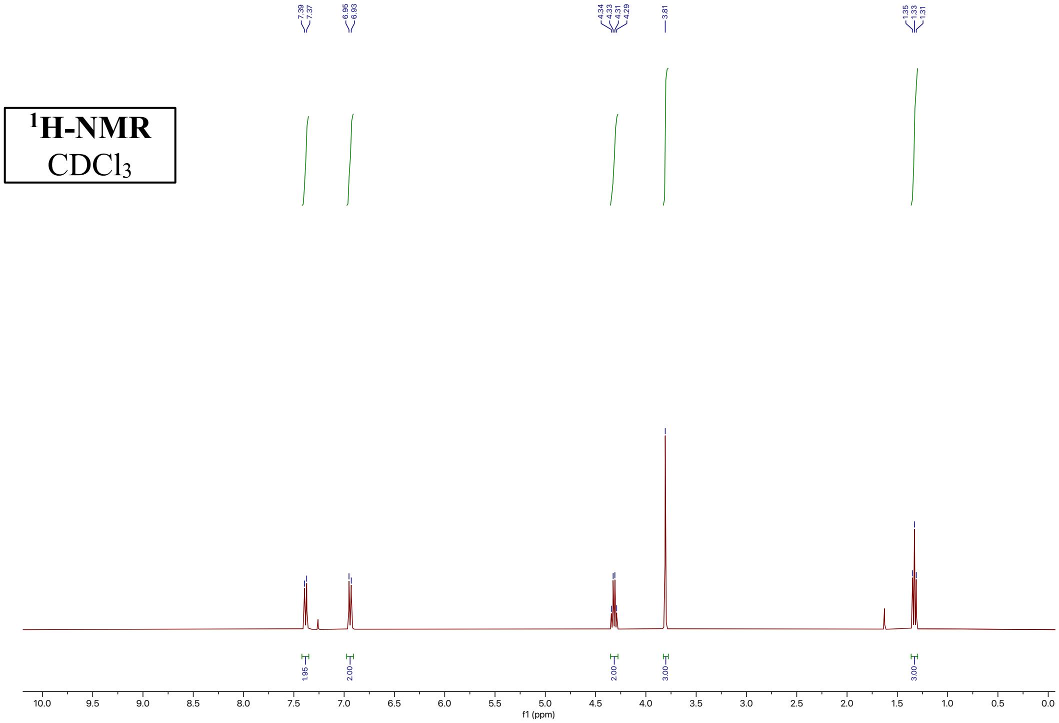

^{13}C-NMR
$CDCl_3$

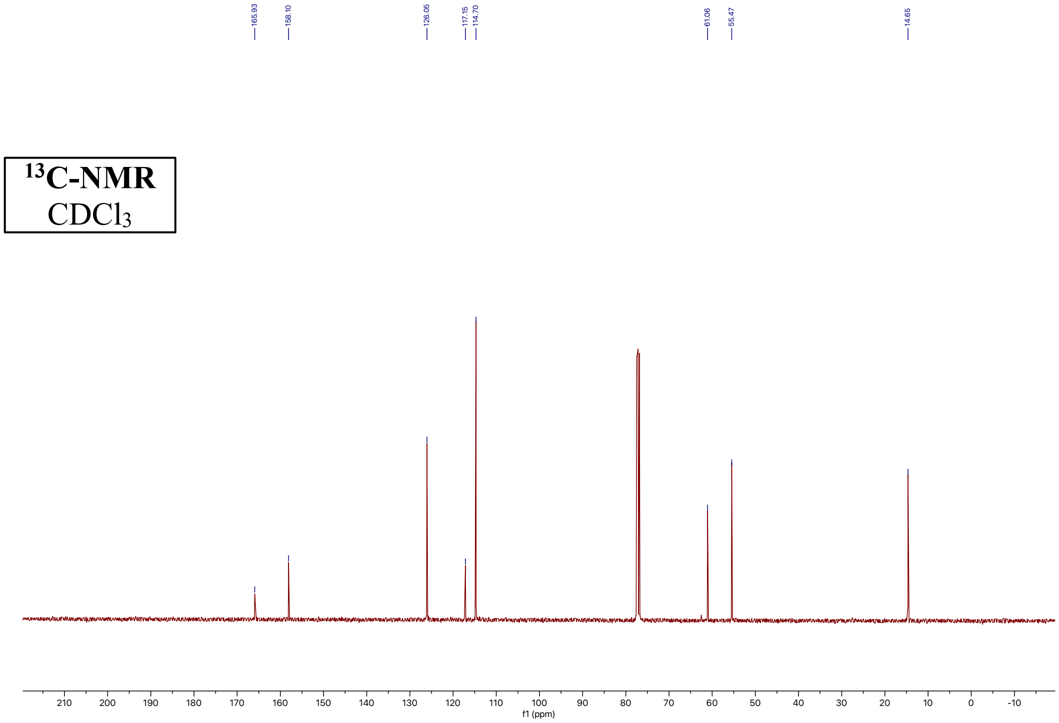

Ethyl 2-(4-chlorophenyl)-2-diazoacetate (6)

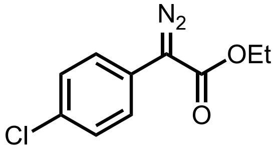

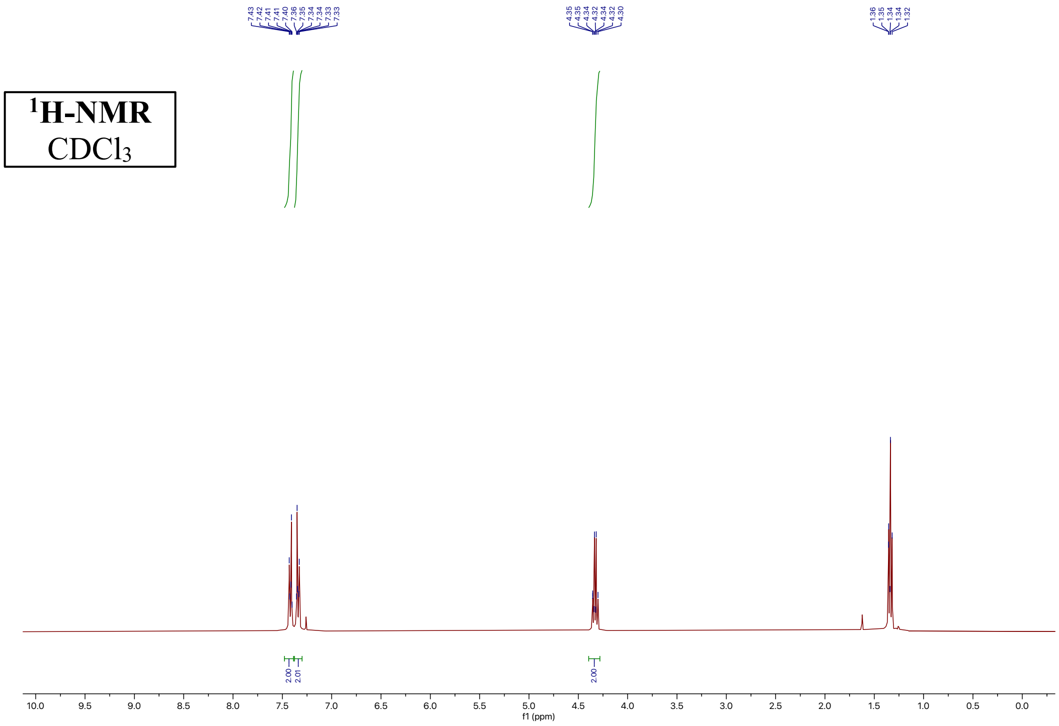

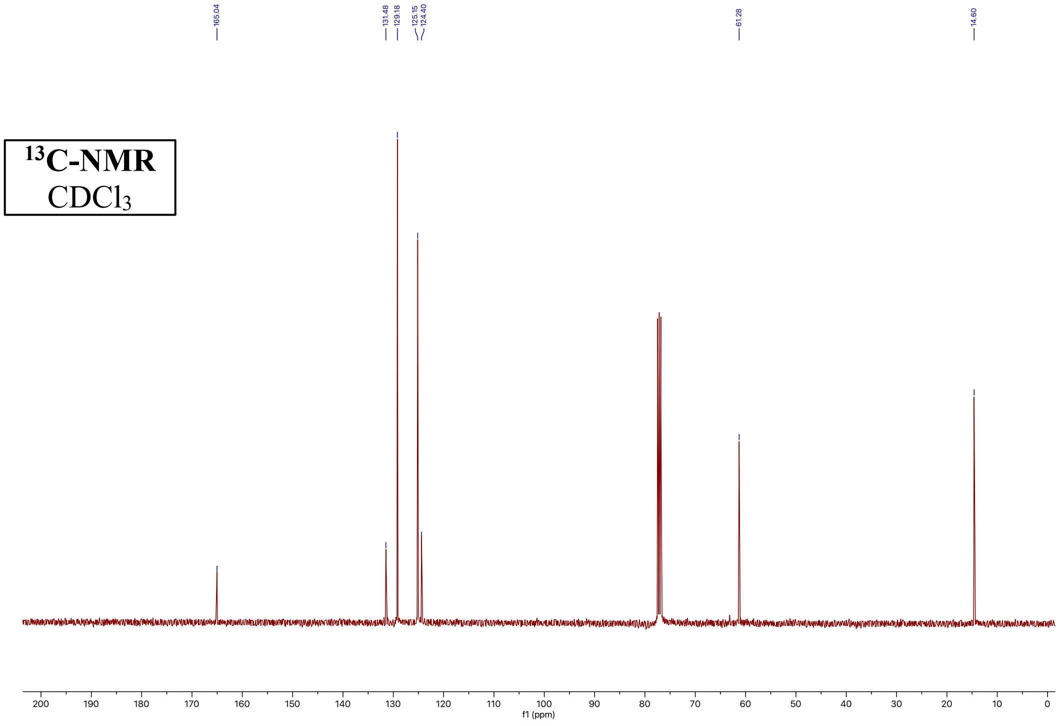

Ethyl 2-(4-bromophenyl)-2-diazoacetate (7)

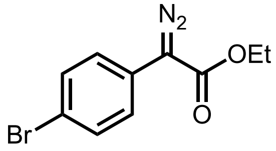

^{1}H-NMR
CDCl$_3$

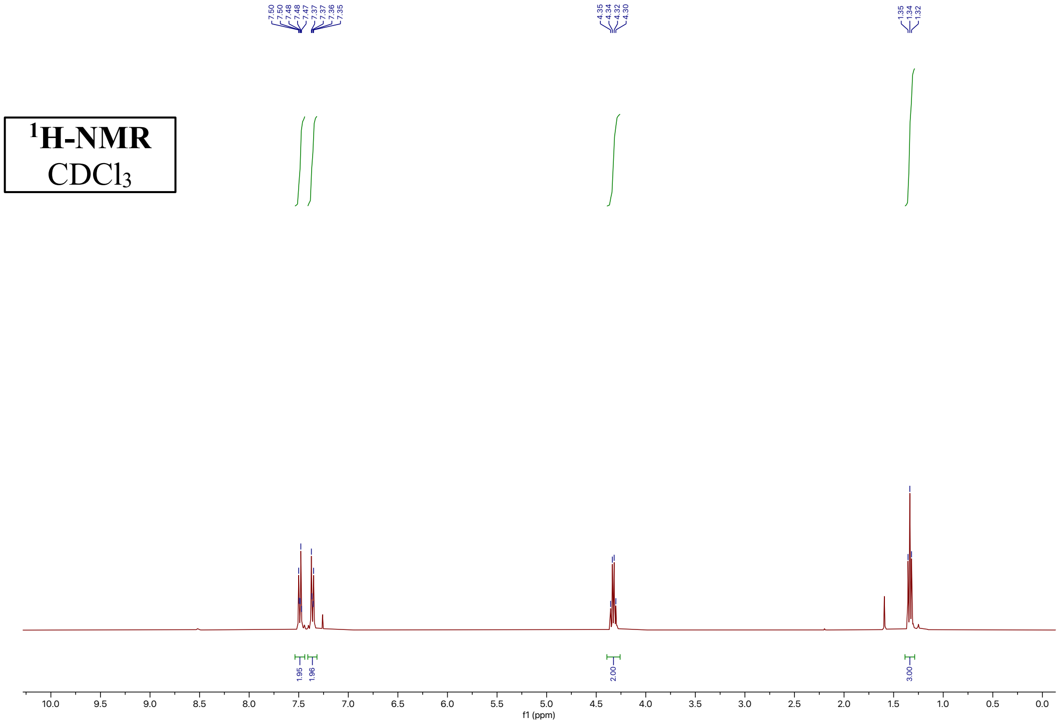

^{13}C-NMR
CDCl$_3$

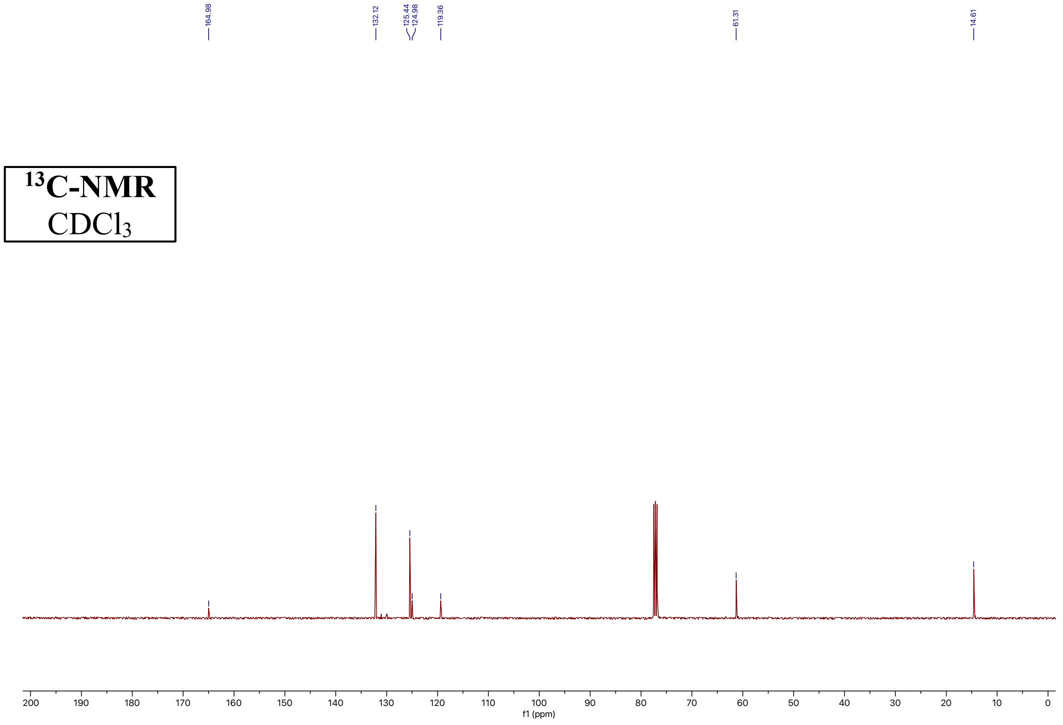

Ethyl 2-diazo-2-(4-fluorophenyl)acetate (8)

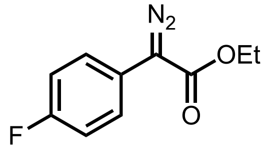

1H-NMR
CDCl3

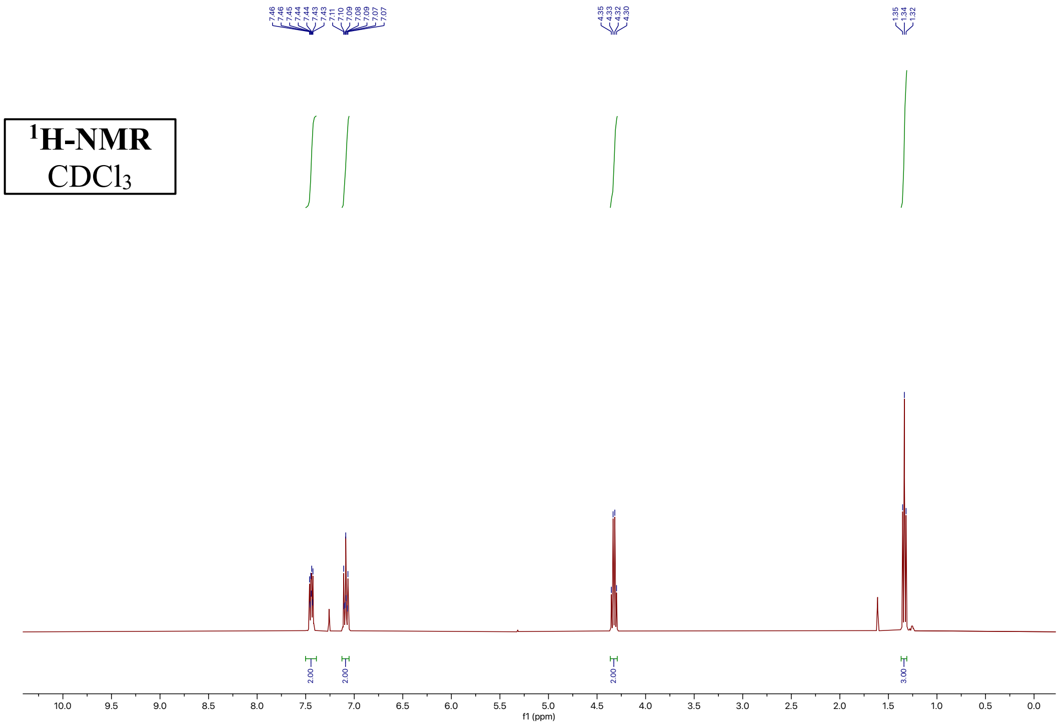

13C-NMR
CDCl3

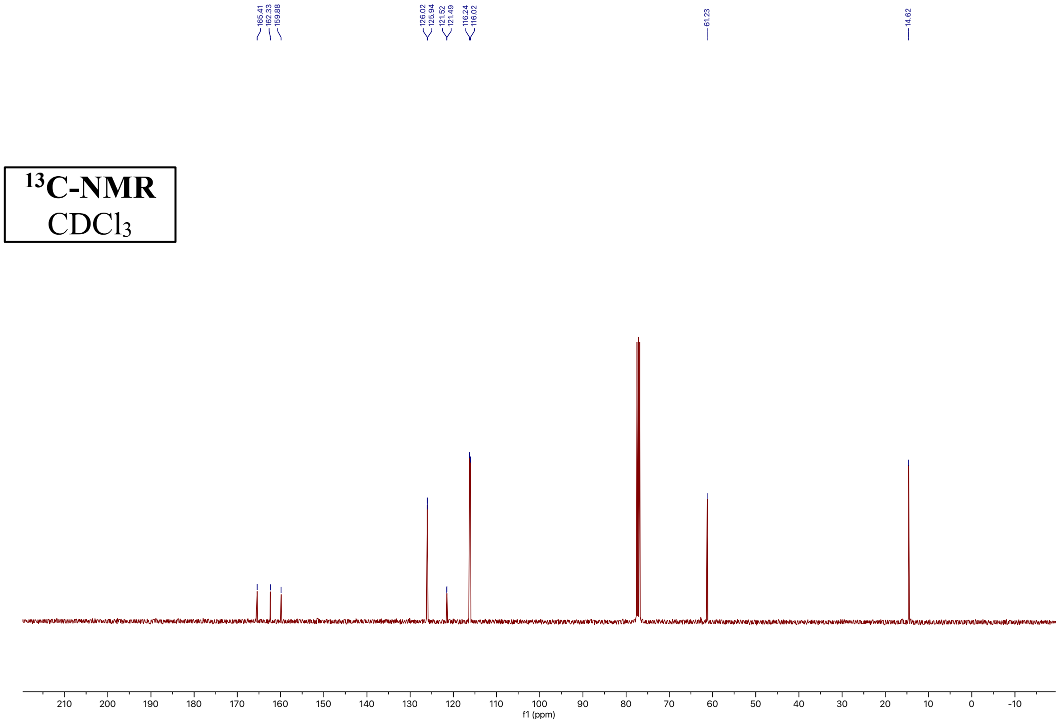

Ethyl 2-(4-cyanophenyl)-2-diazoacetate (9)

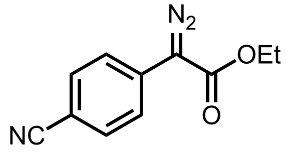

^{1}H-NMR
$CDCl_3$

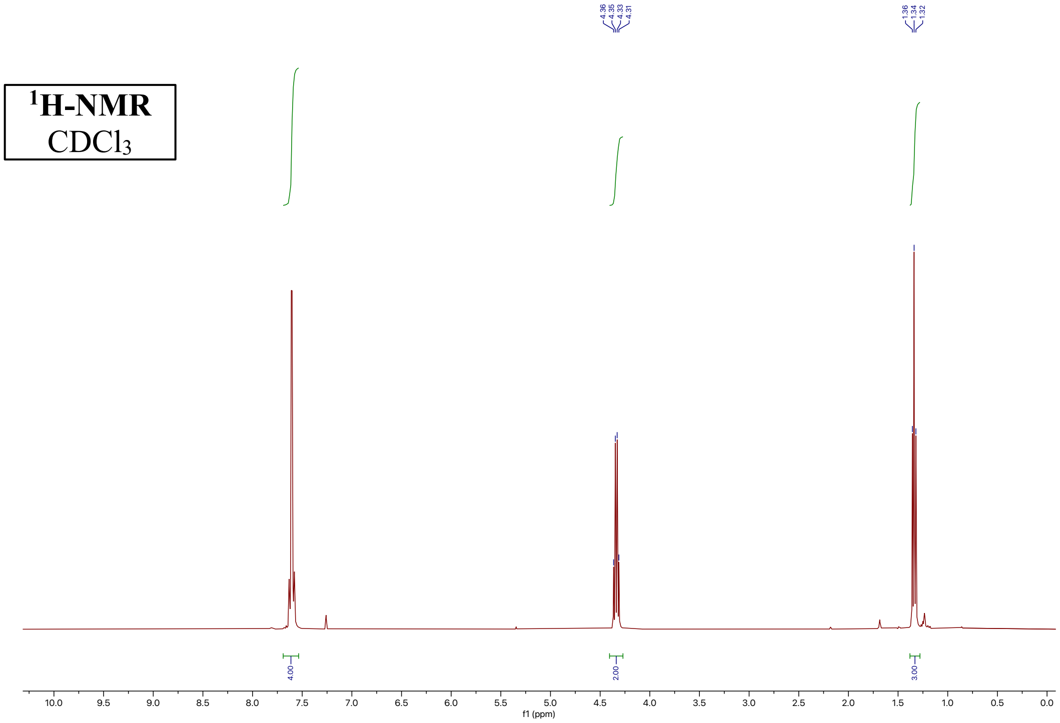

^{13}C-NMR
$CDCl_3$

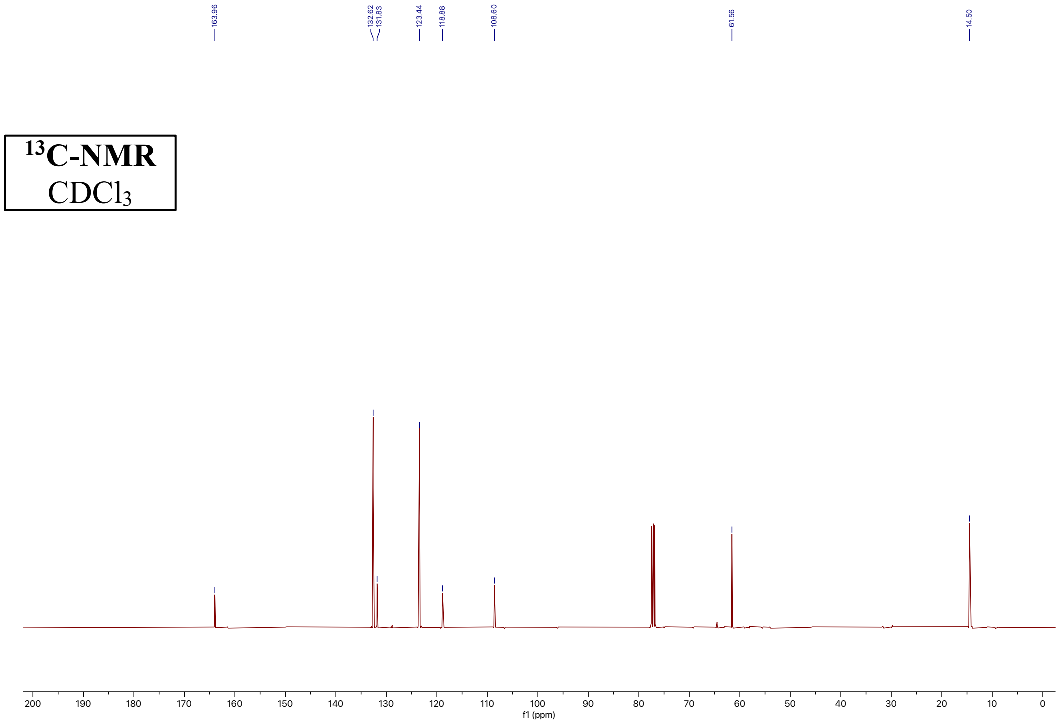

Ethyl 2-diazo-2-(3-methoxyphenyl)acetate (10)

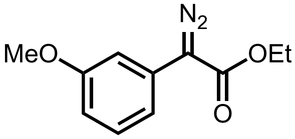

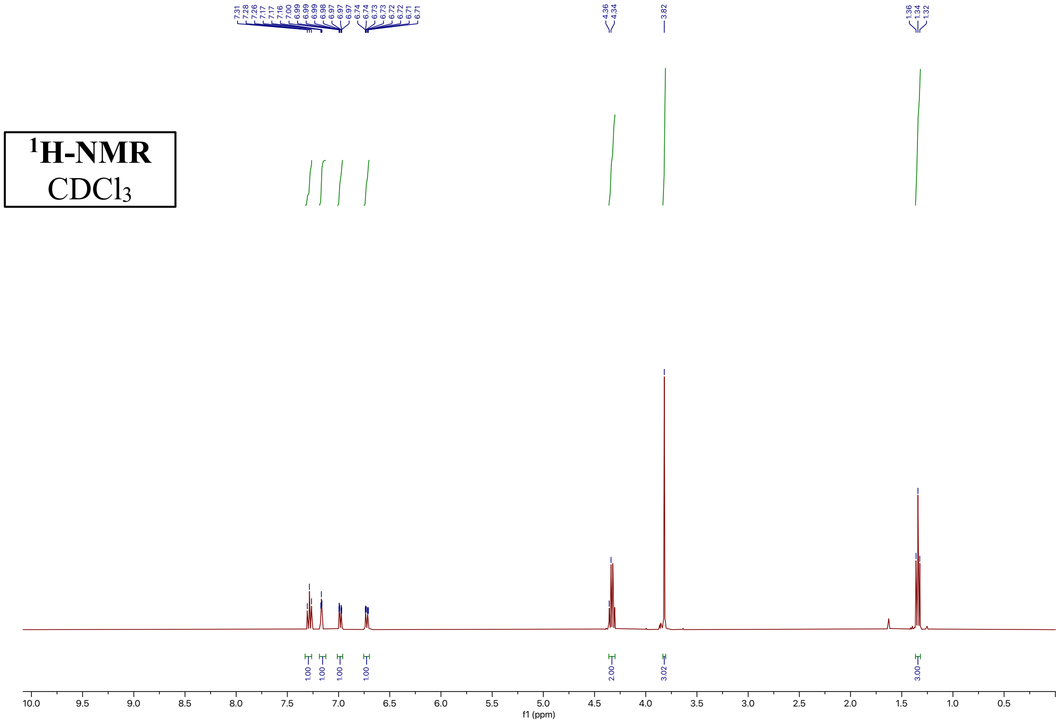

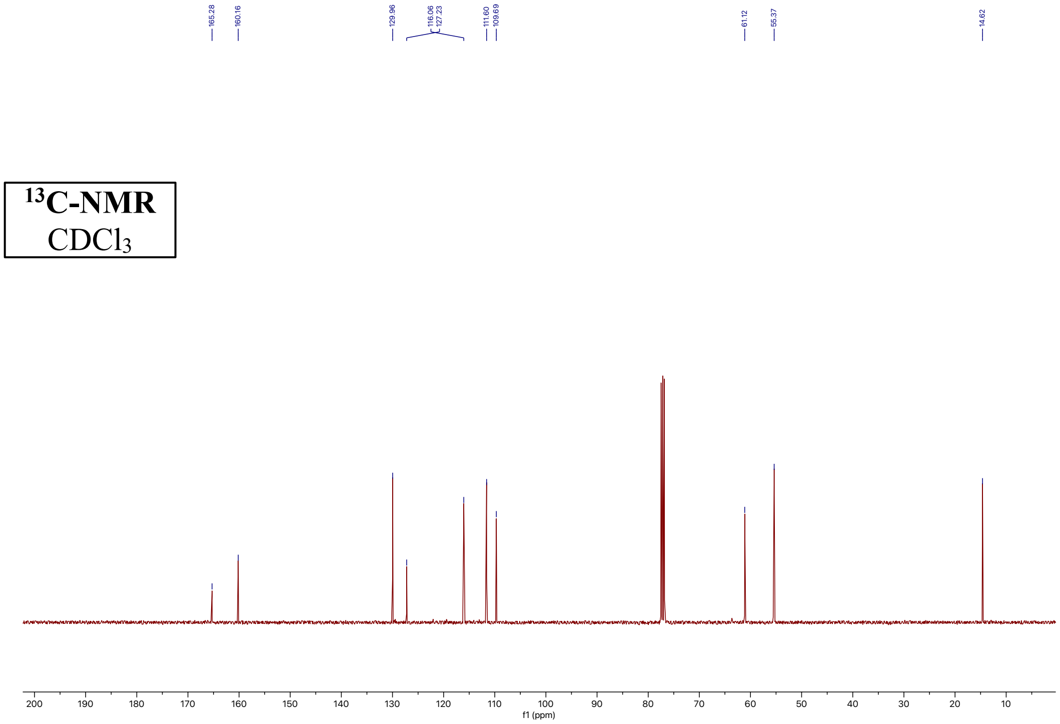

3-Diazo-1-phenylindolin-2-one (11)

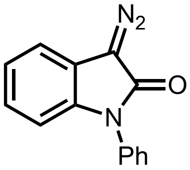

^{1}H-NMR
$CDCl_3$

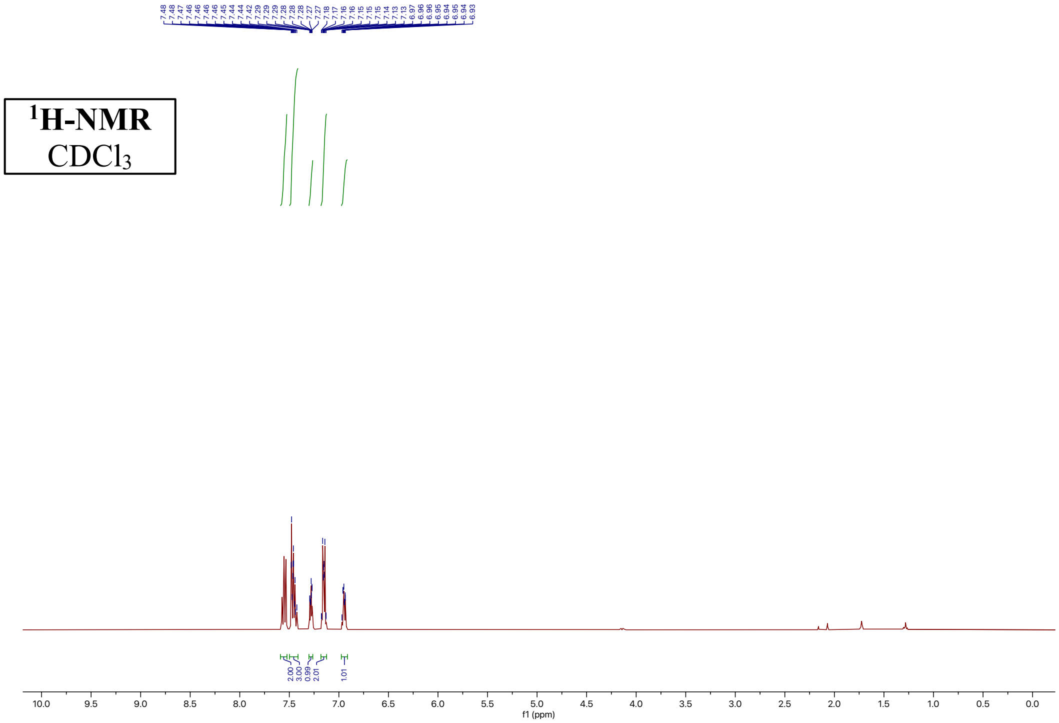

^{13}C-NMR
$CDCl_3$

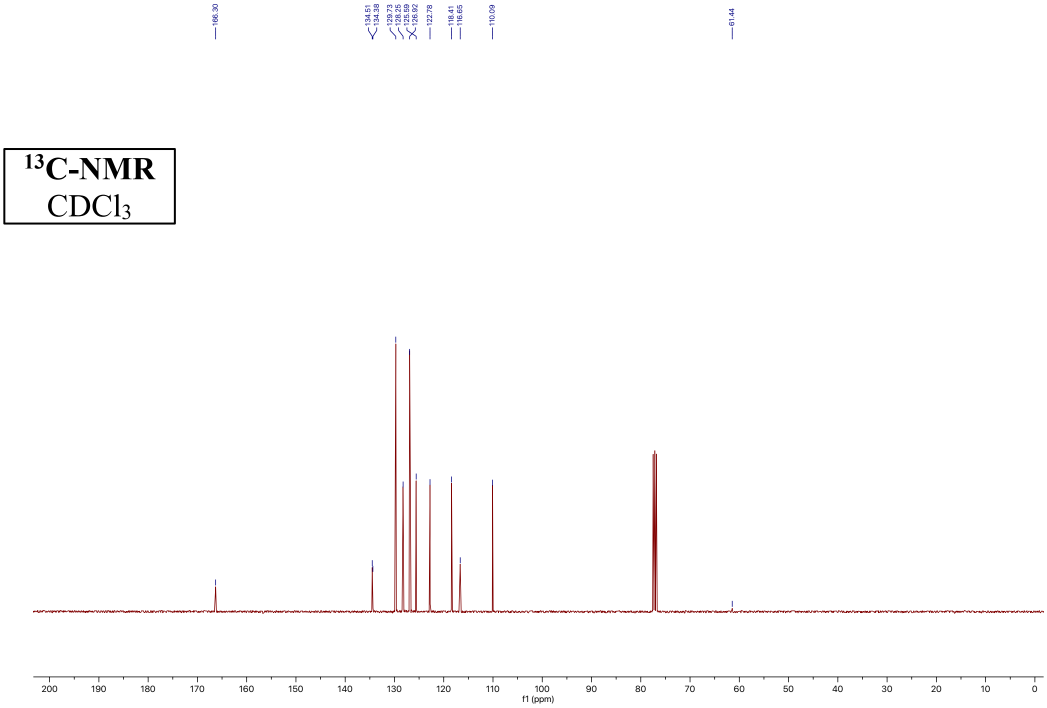

3-Diazo-1-methylindolin-2-one (12)

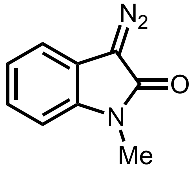

^{1}H-NMR
$CDCl_3$

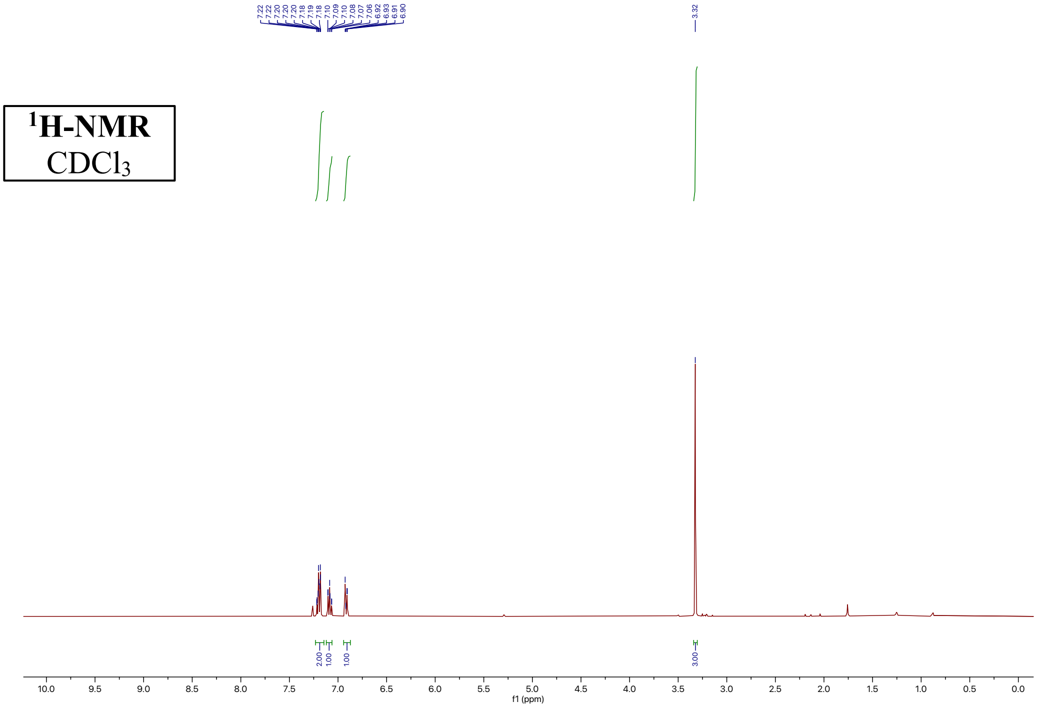

^{13}C-NMR
$CDCl_3$

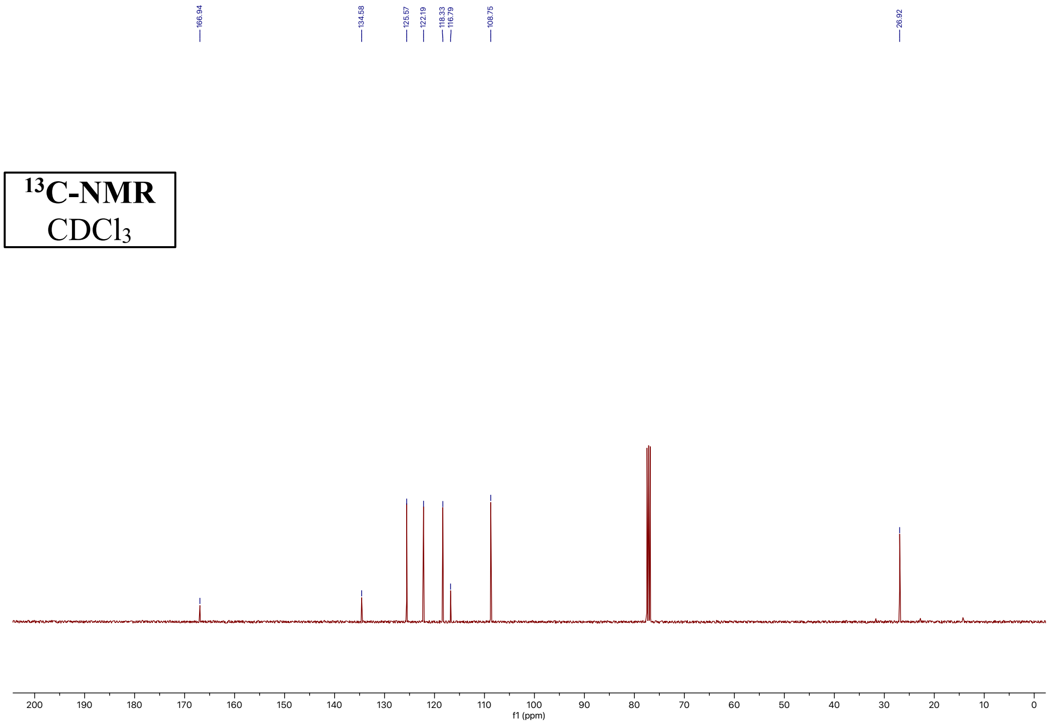

1-Benzyl-3-diazoindolin-2-one (13)

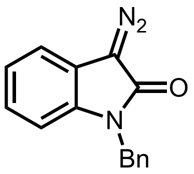

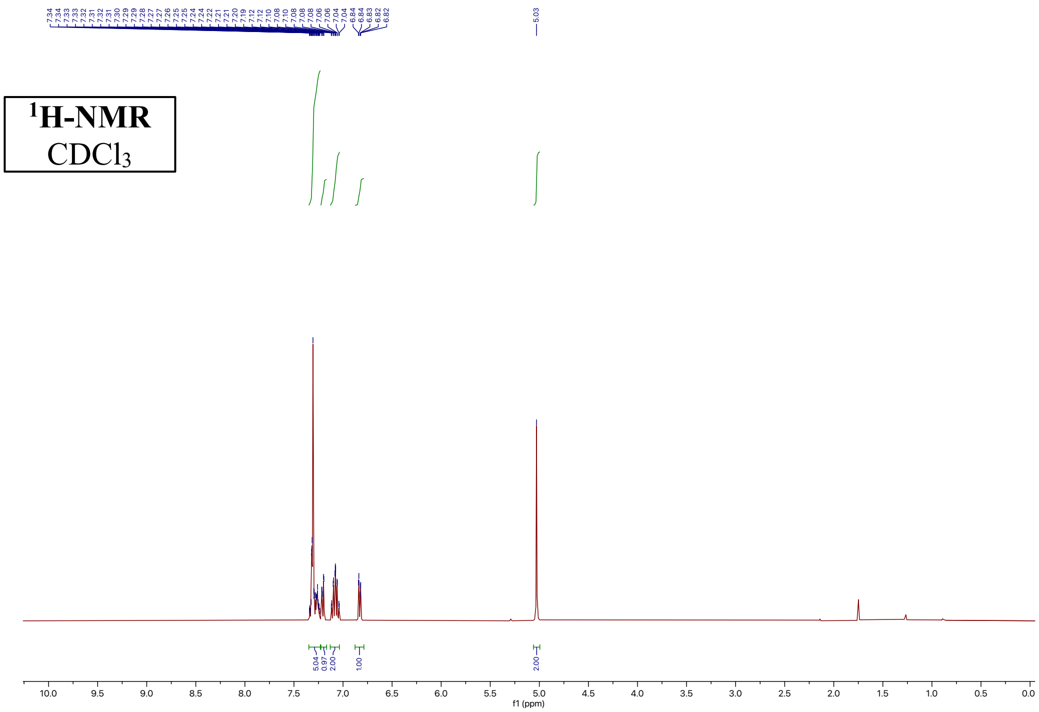

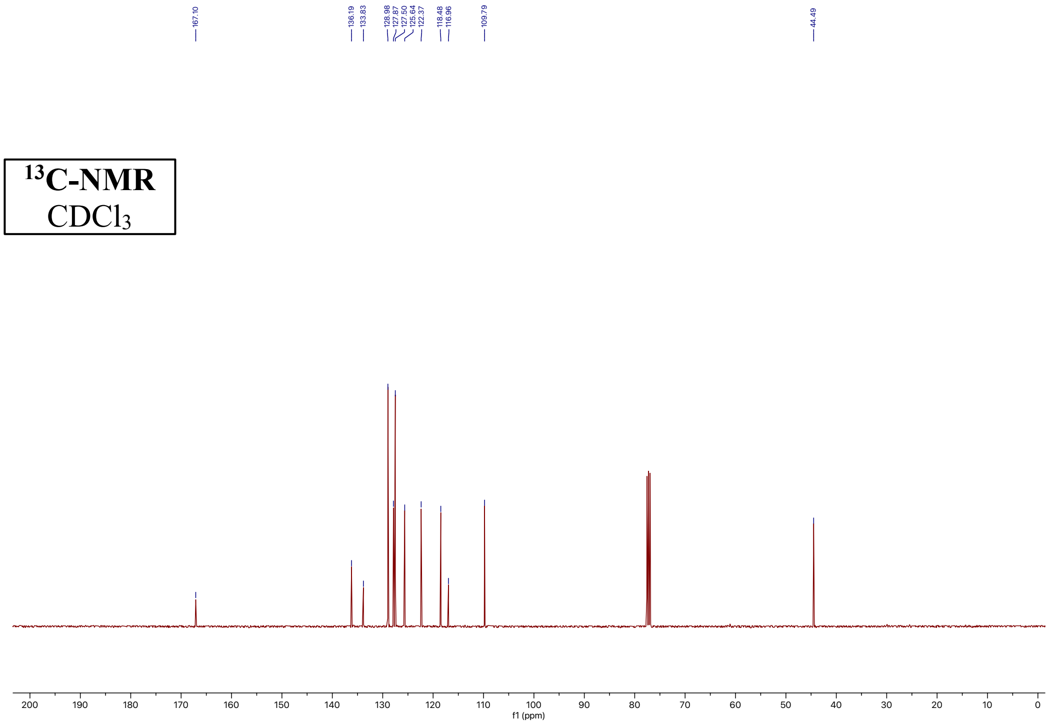

1-Benzyl-3-diazo-5-methylindolin-2-one (14)

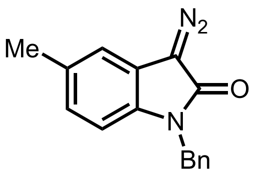

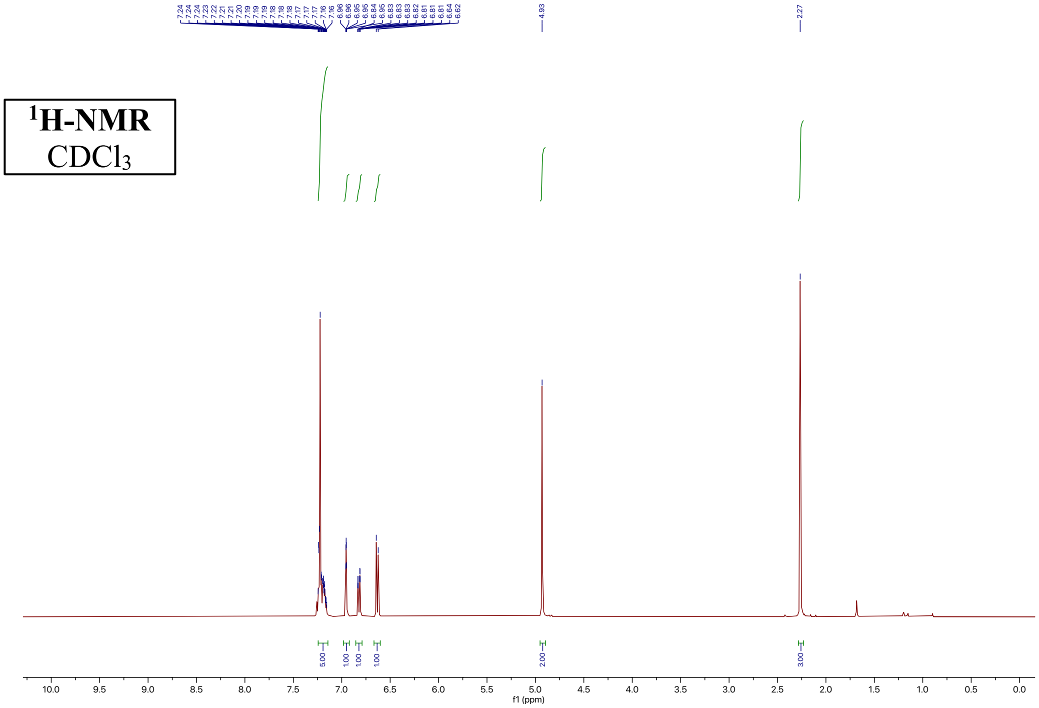

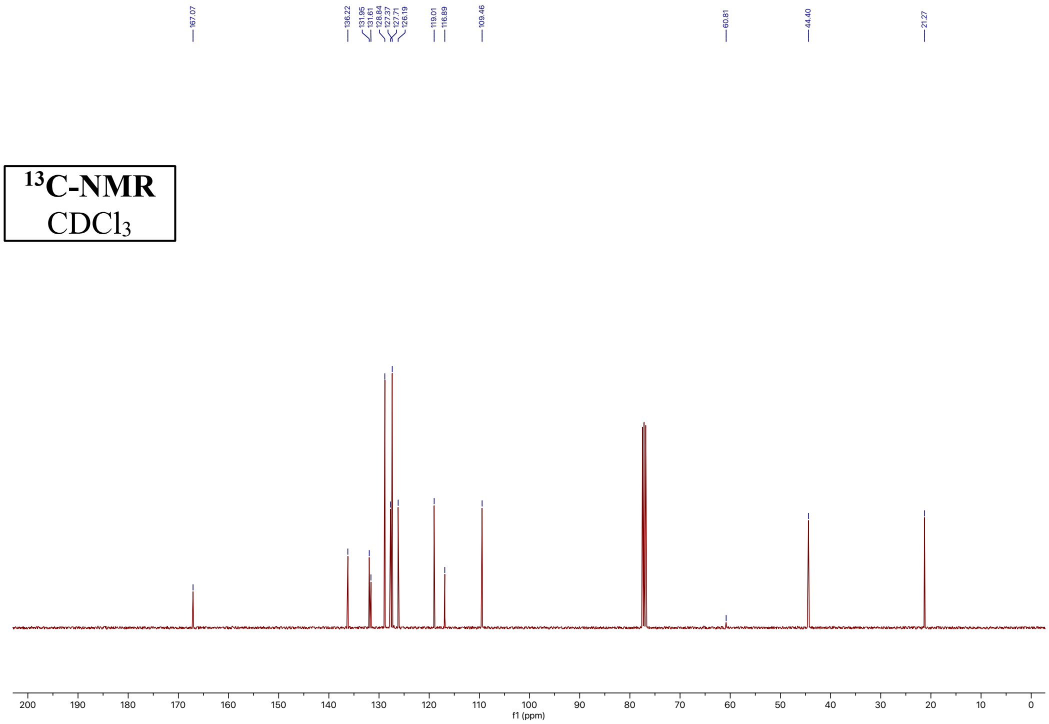

1-Benzyl-3-diazo-5-methoxyindolin-2-one (15)

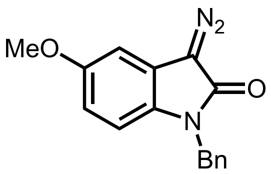

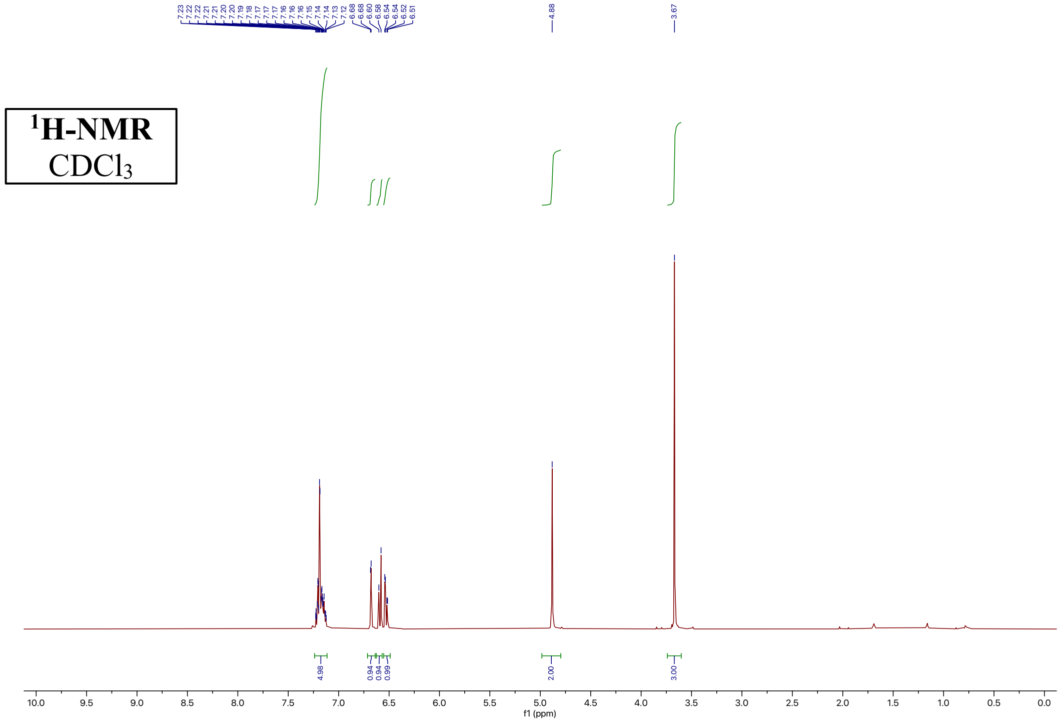

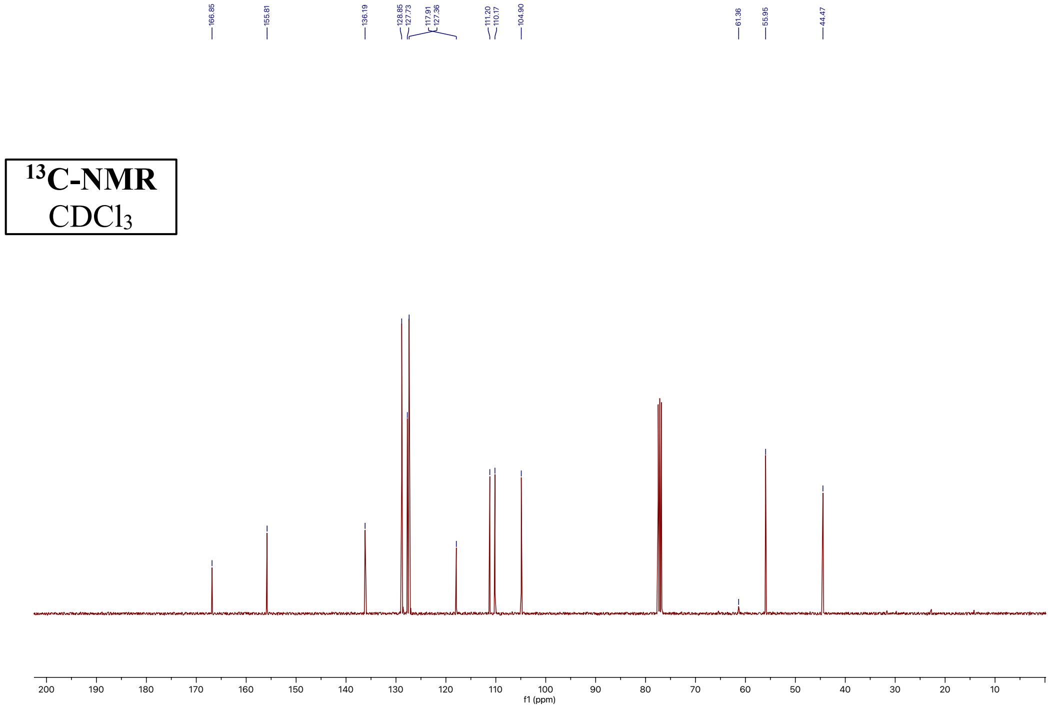

1-Benzyl-3-diazo-7-methylindolin-2-one (16)

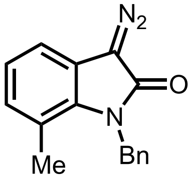

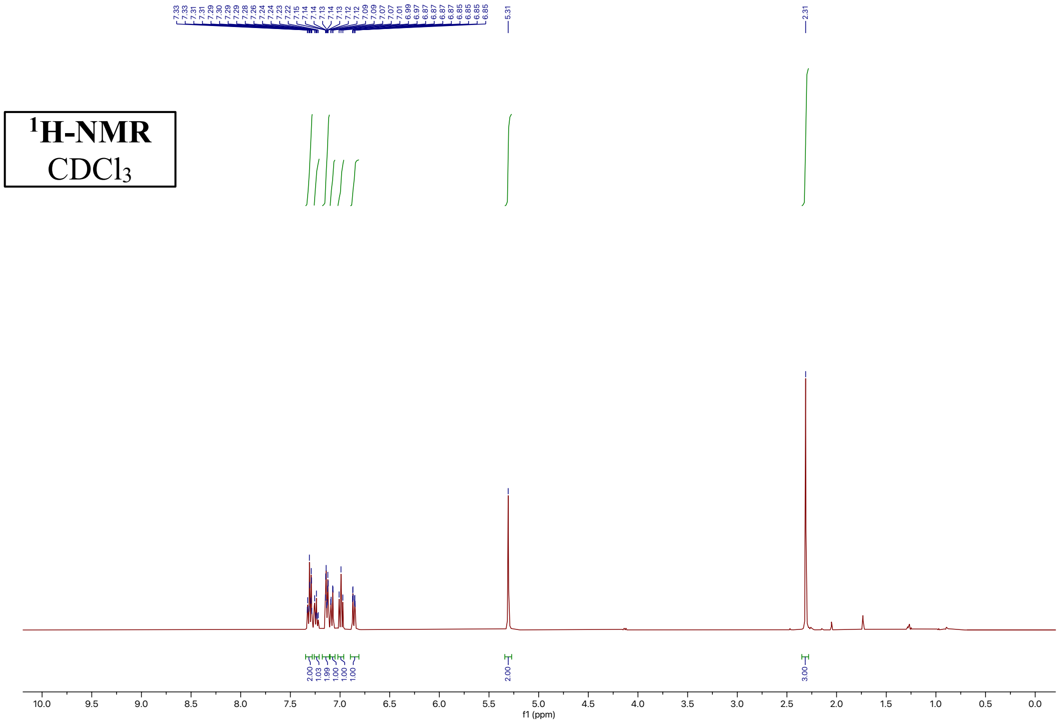

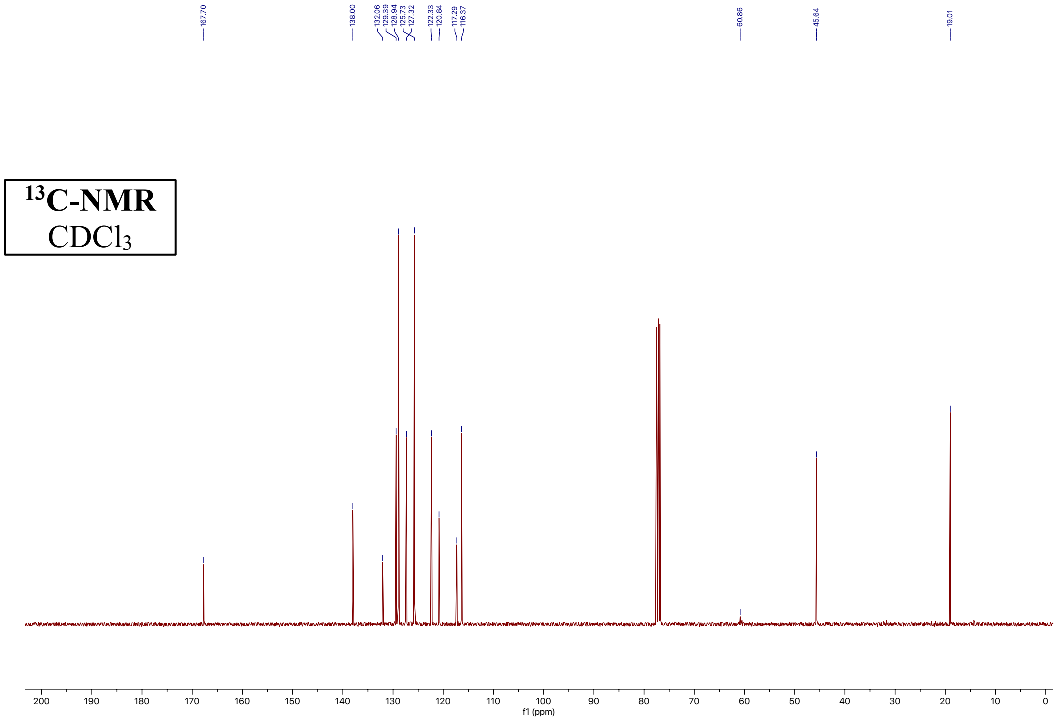

3-Diazoindolin-2-one (17)

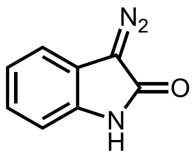

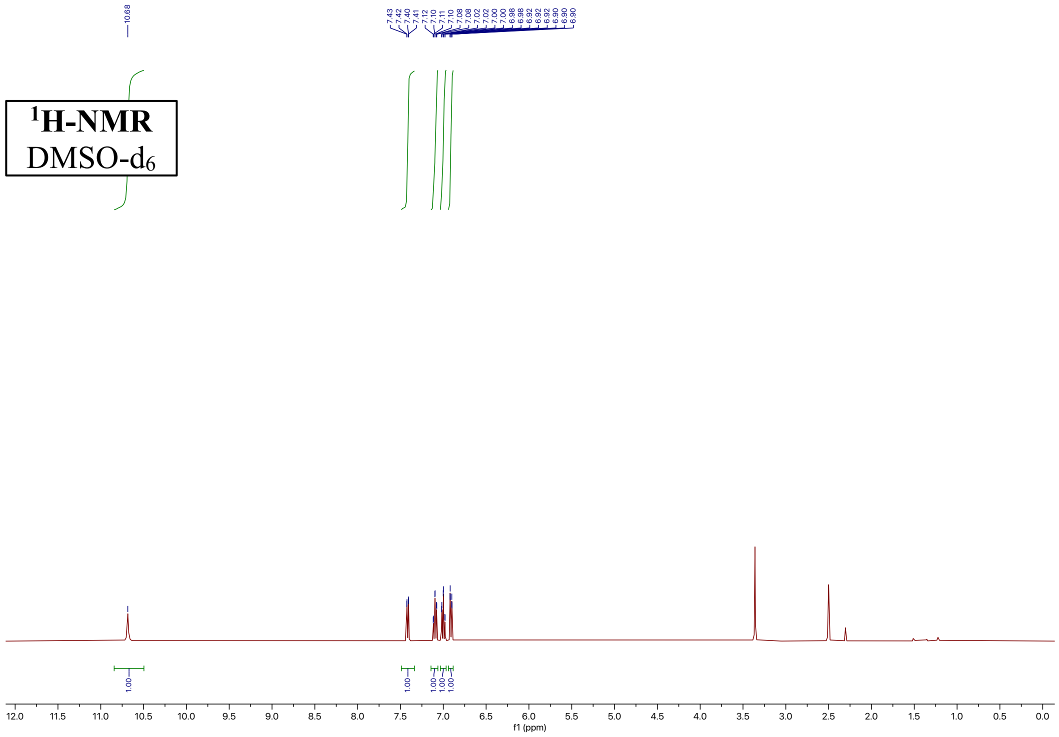

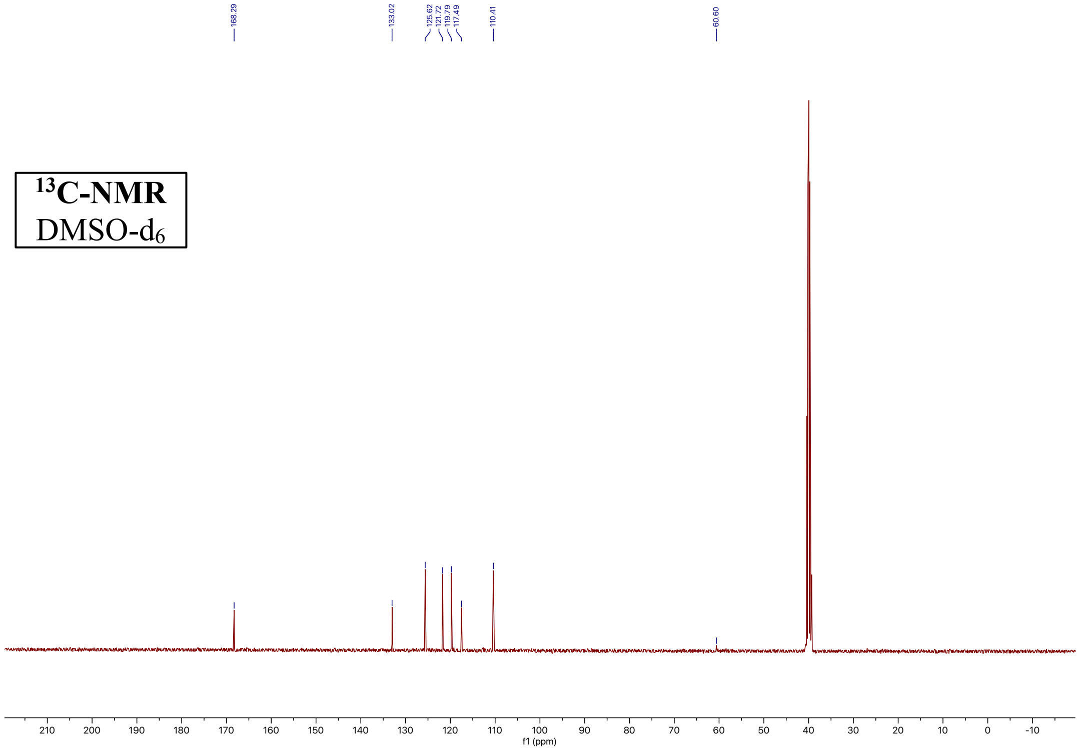

Benzyl 2-diazopropanoate (18)

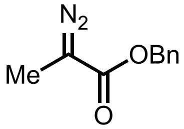

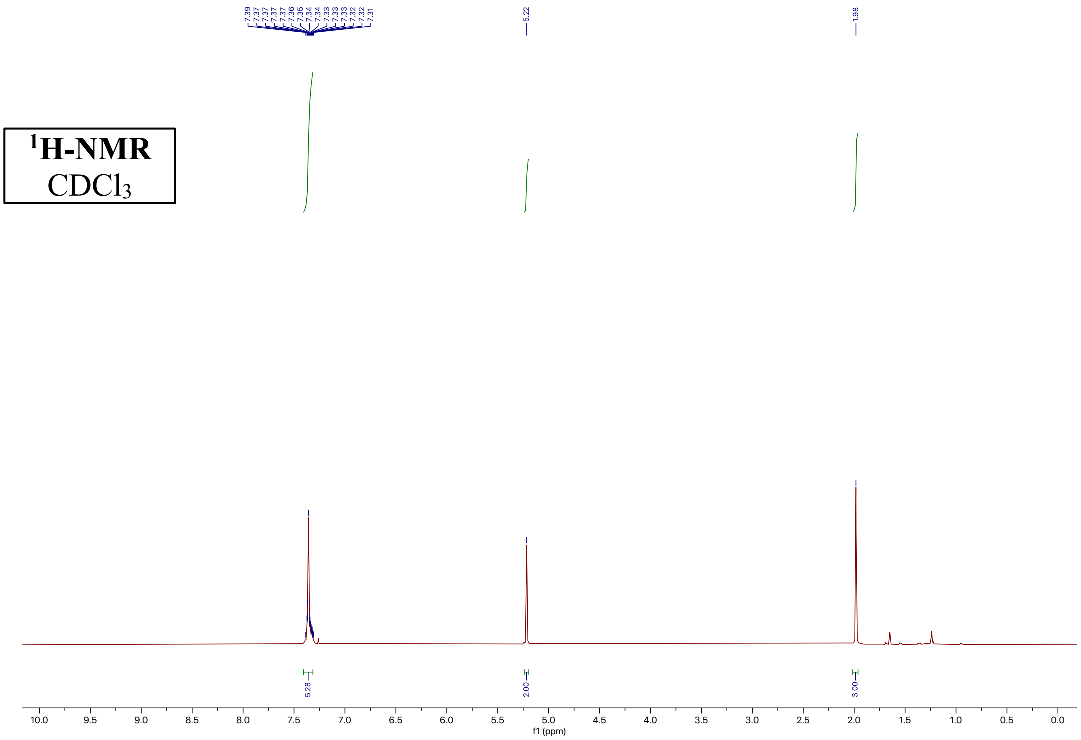

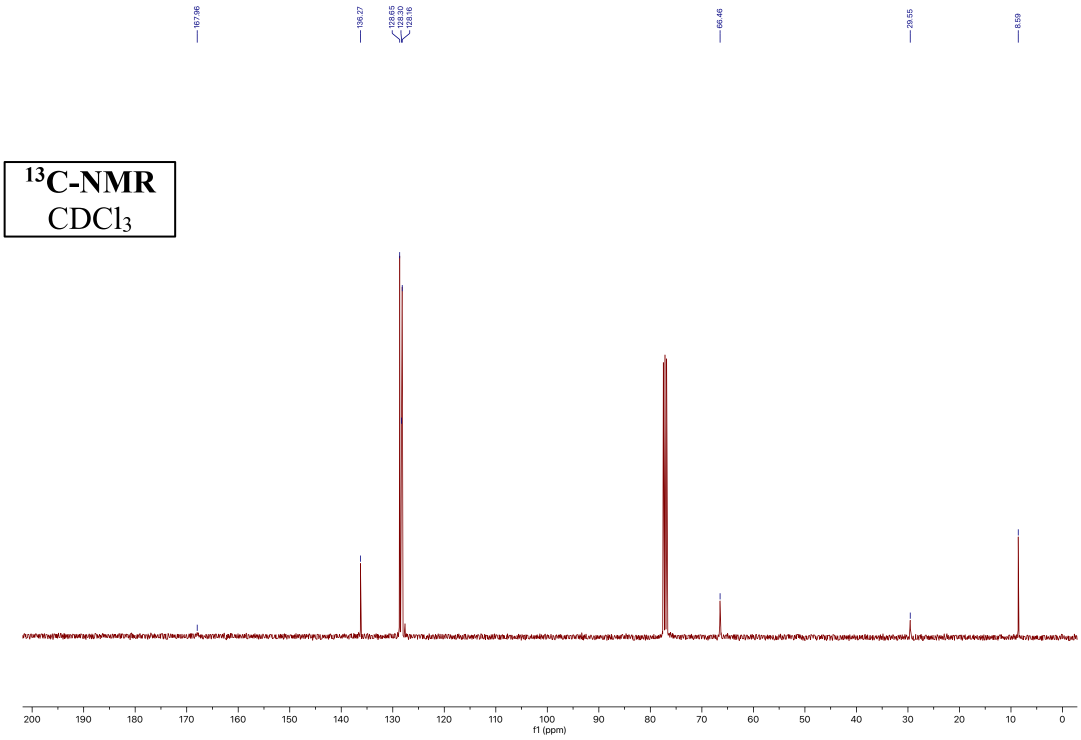

Diazomethylene)dibenzene (19)

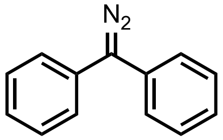

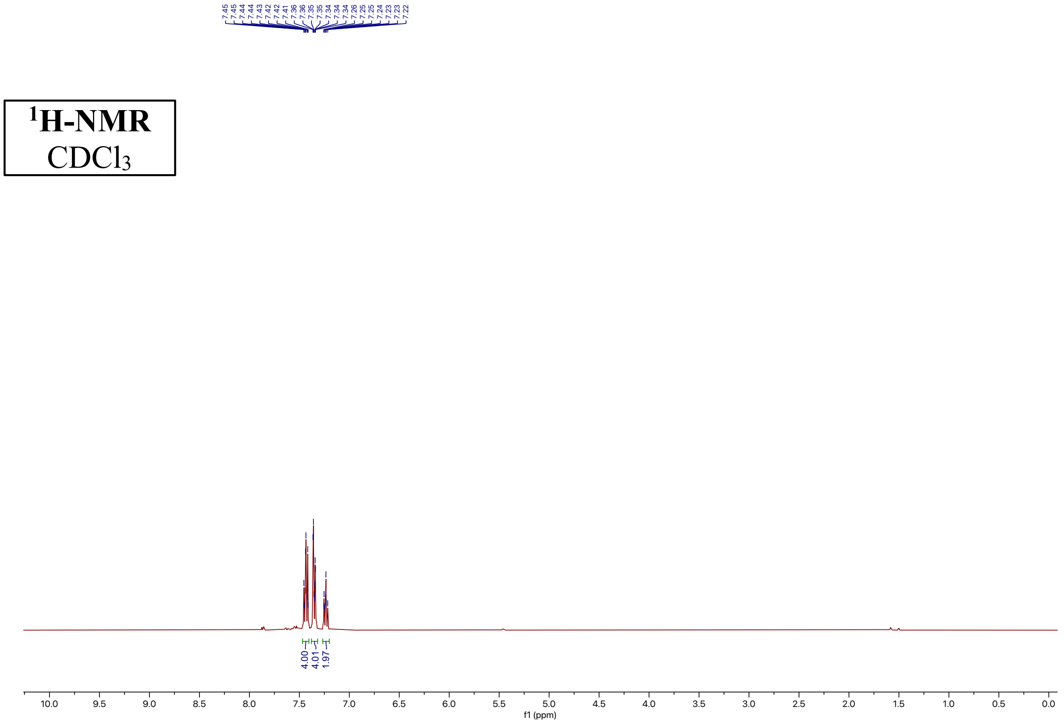

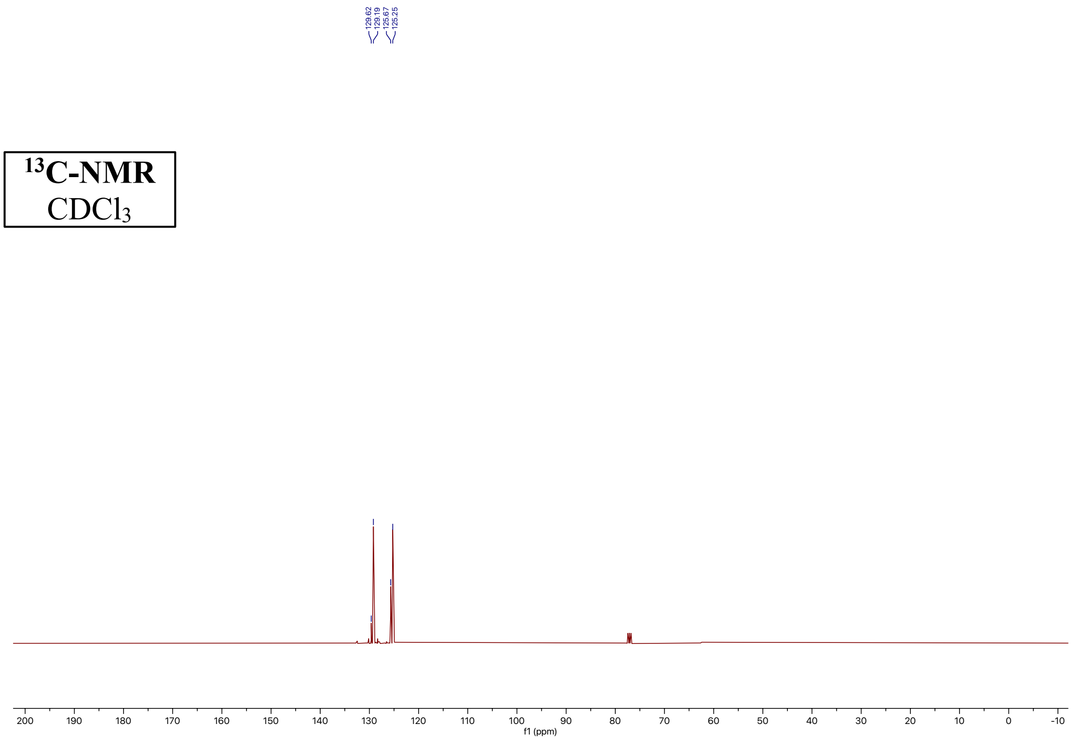

1-(Diazo(phenyl)methyl)-4-methoxybenzene (20)

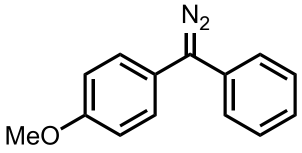

^{1}H-NMR
CDCl3

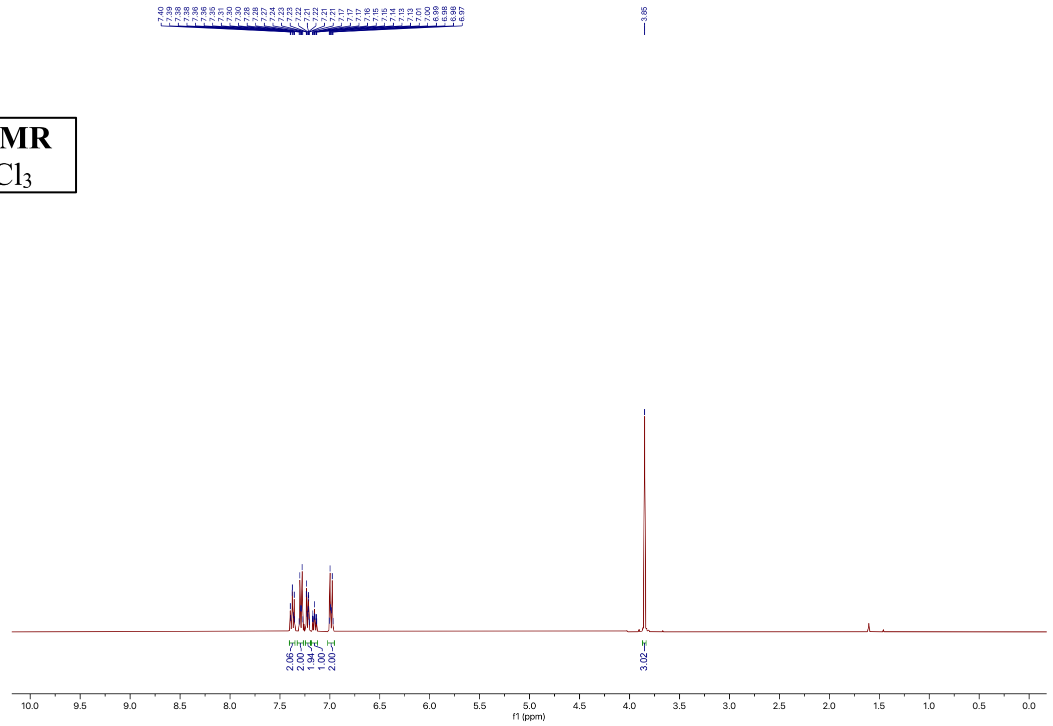

^{13}C-NMR
CDCl3

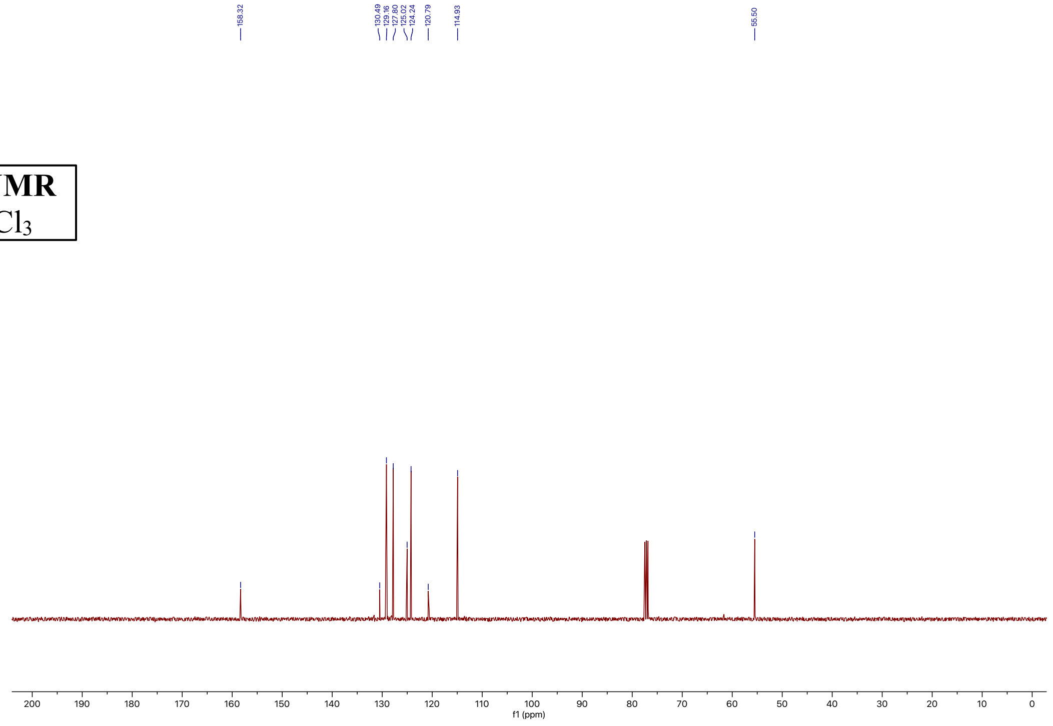

1-(Diazo(4-methoxyphenyl)methyl)-4-nitrobenzene (21)

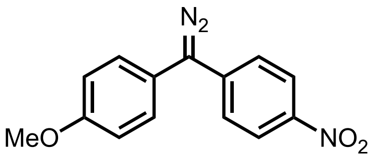

^{1}H-NMR
$CDCl_3$

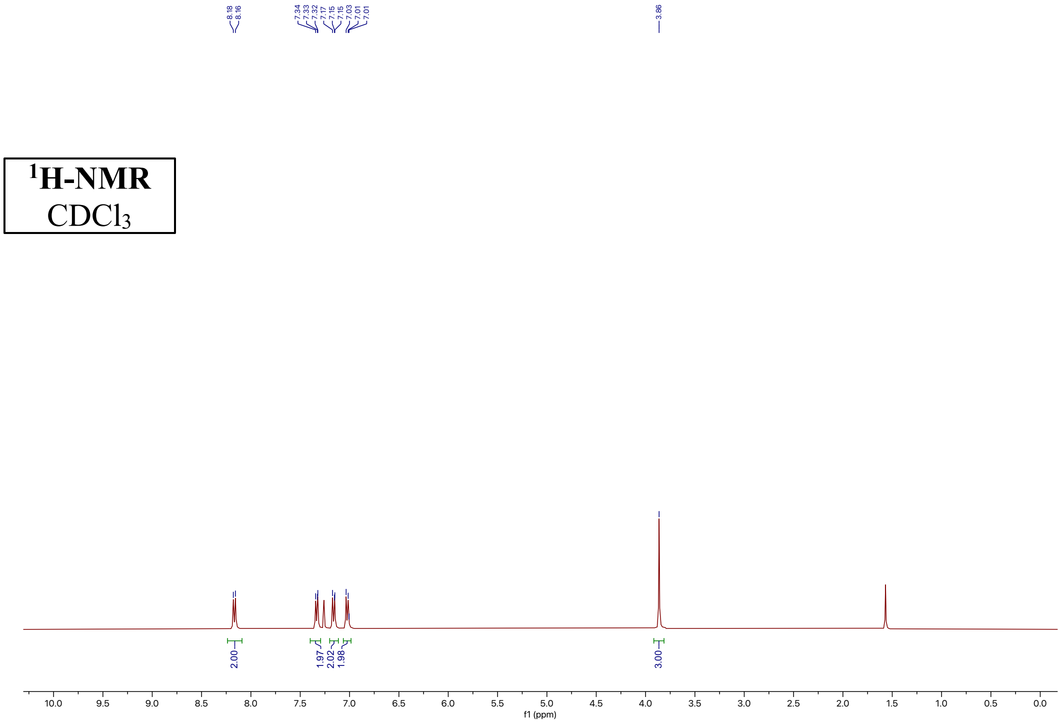

^{13}C-NMR
$CDCl_3$

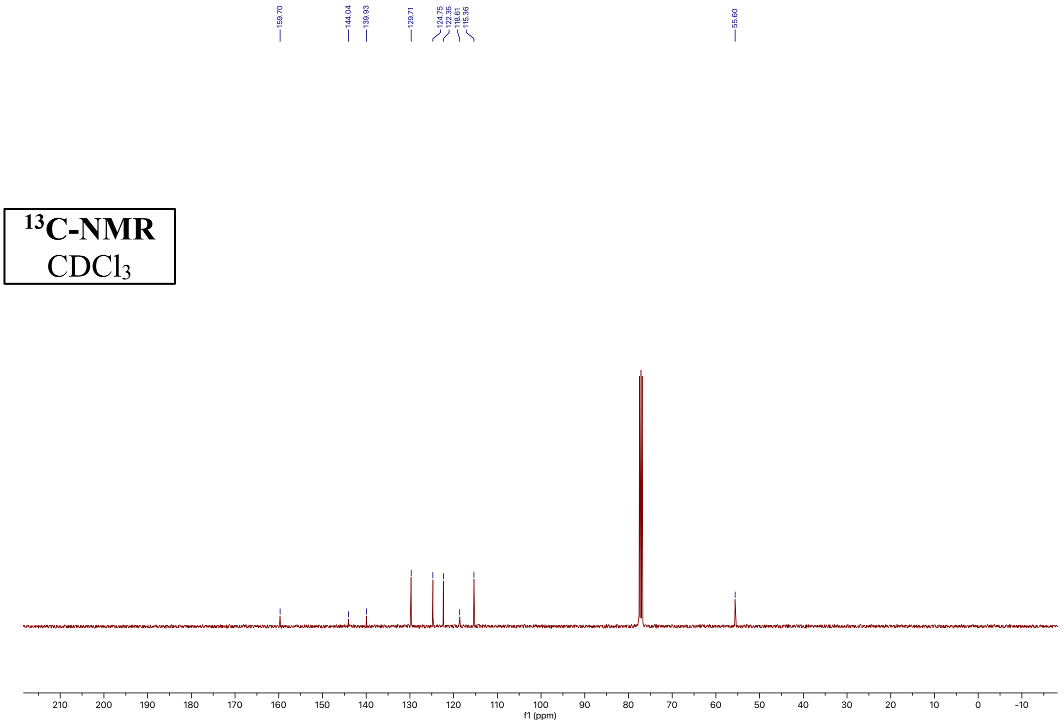

Methyl 2-diazo-2-phenylacetate (23)

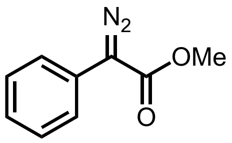

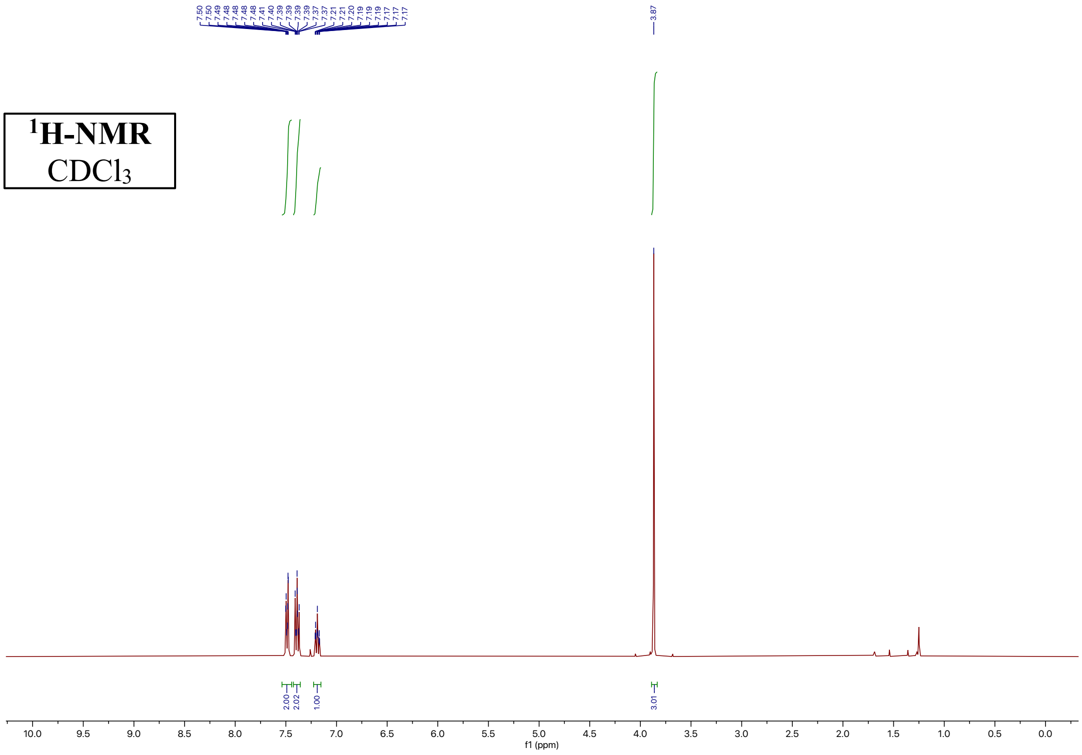

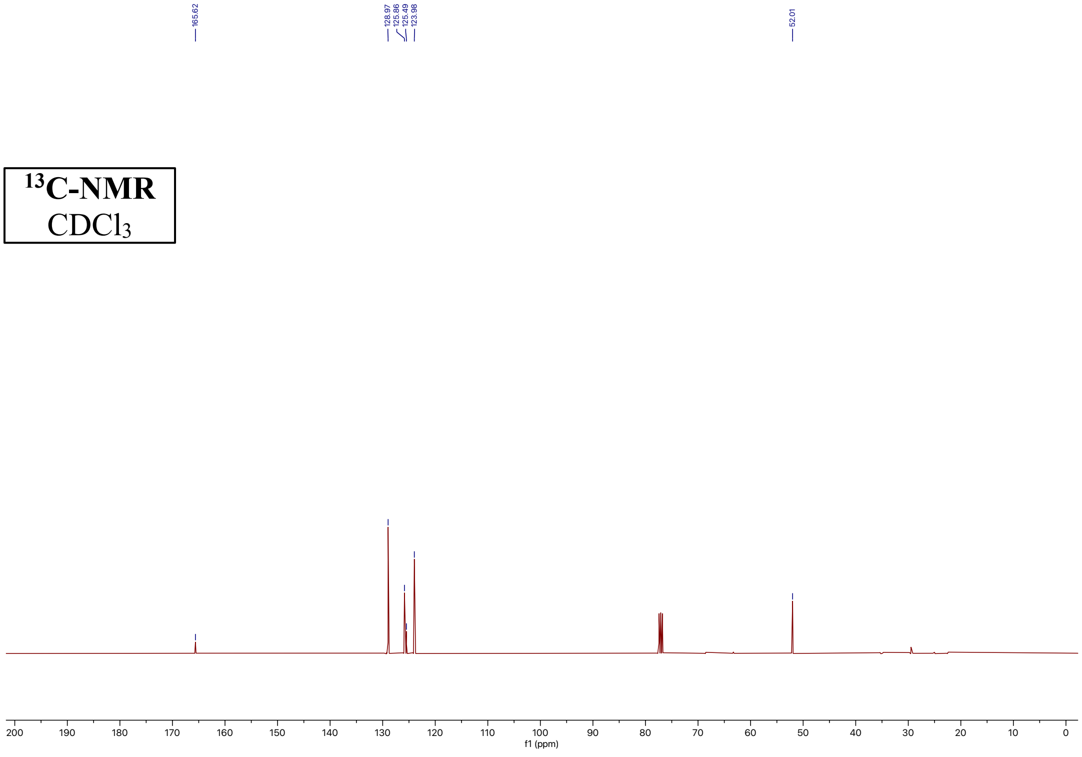

Propyl 2-diazo-2-phenylacetate (24)

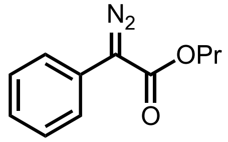

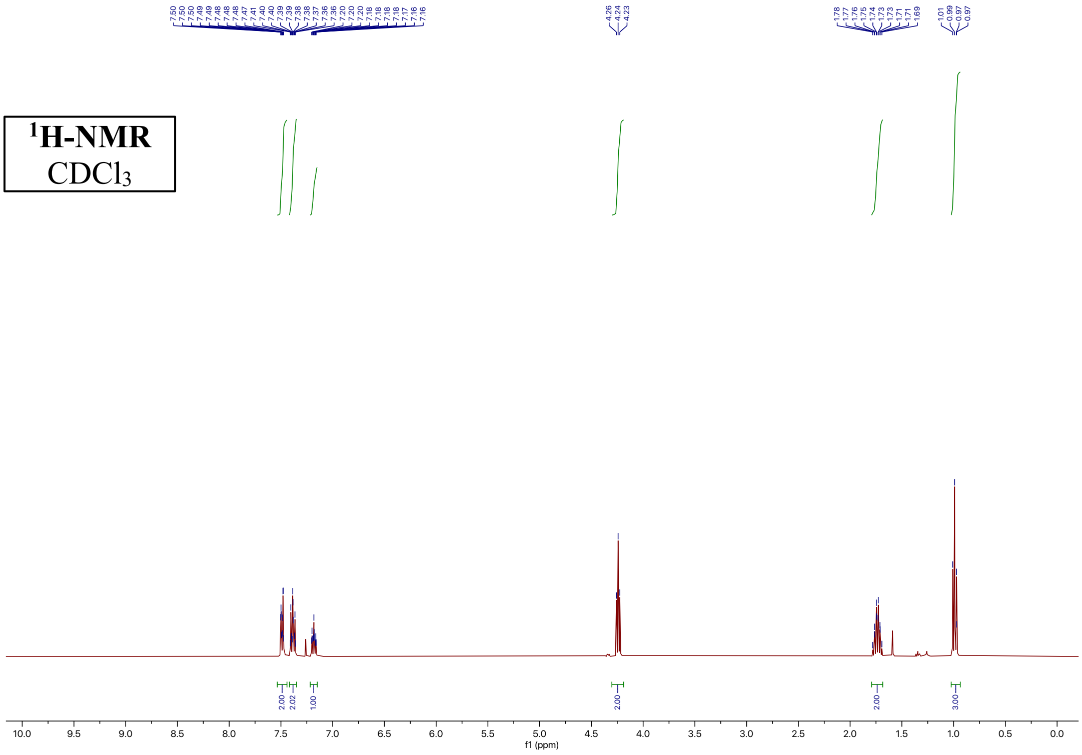

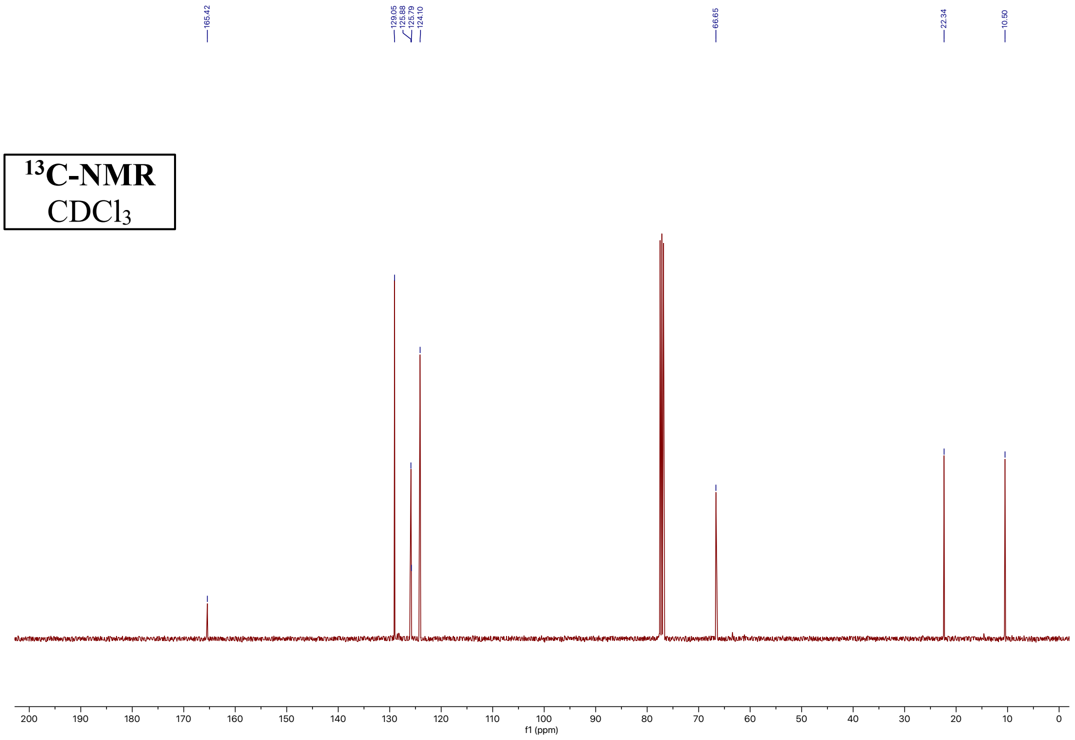

Isopropyl 2-diazo-2-phenylacetate (25)

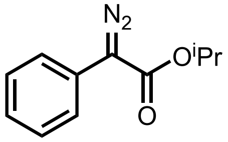

^{1}H-NMR
$CDCl_3$

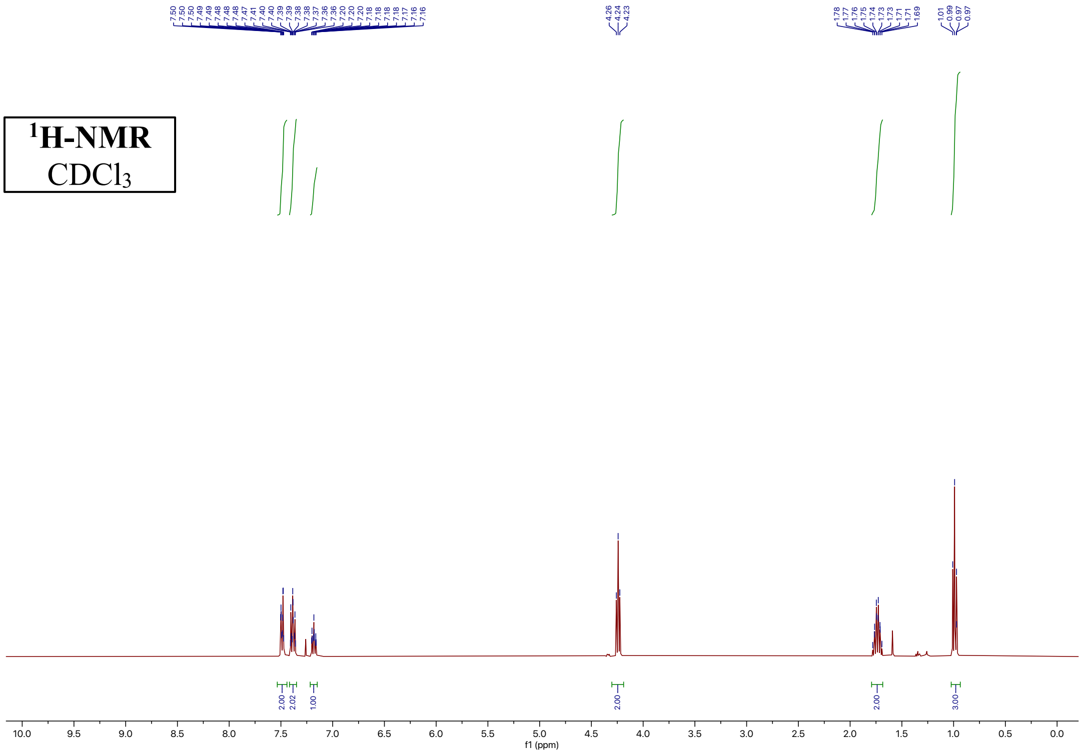

^{13}C-NMR
$CDCl_3$

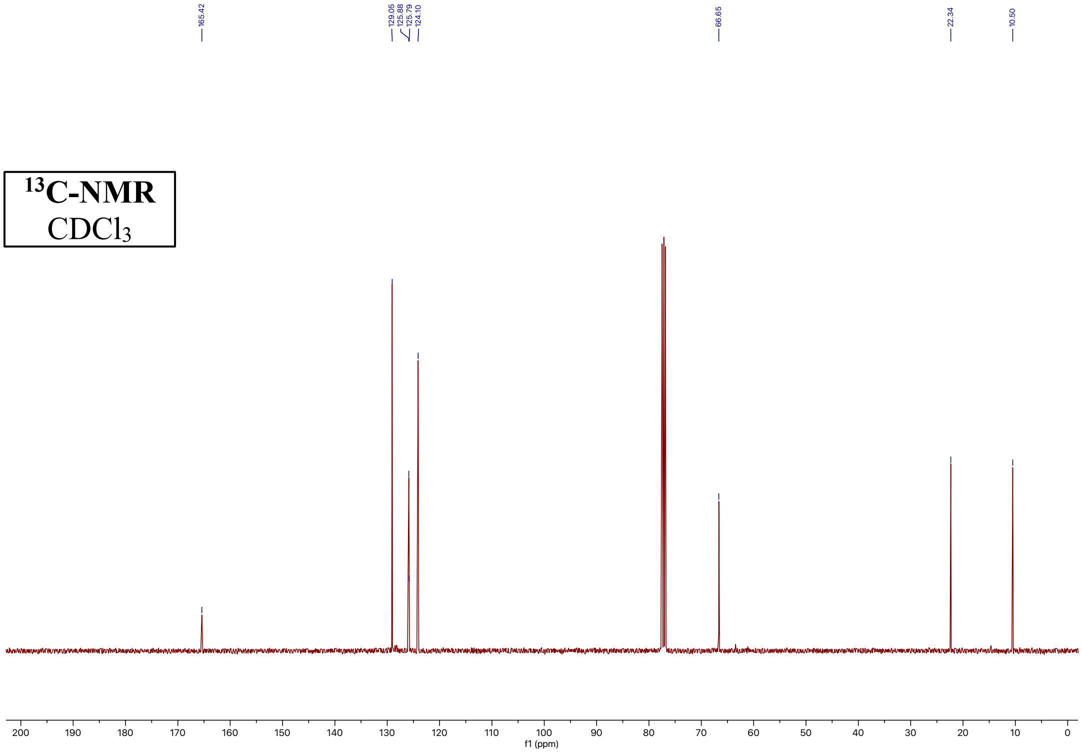

Butyl 2-diazo-2-phenylacetate (26)

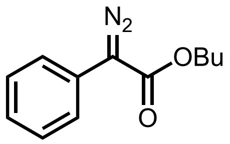

^{1}H-NMR
CDCl3

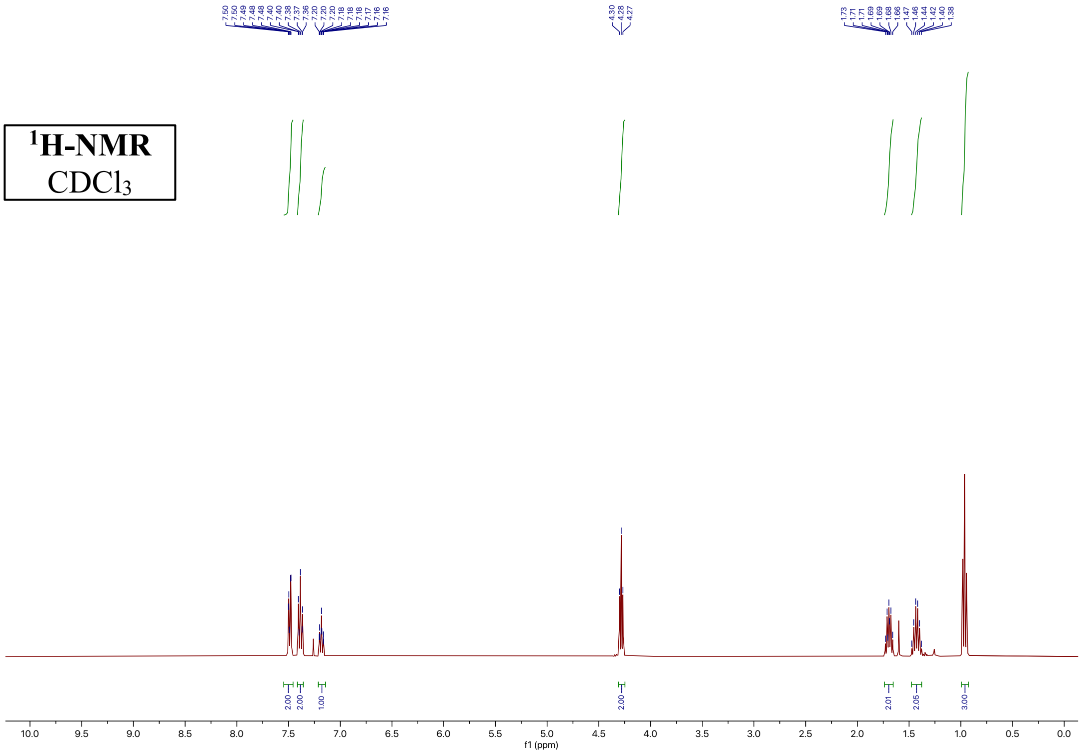

^{13}C-NMR
CDCl3

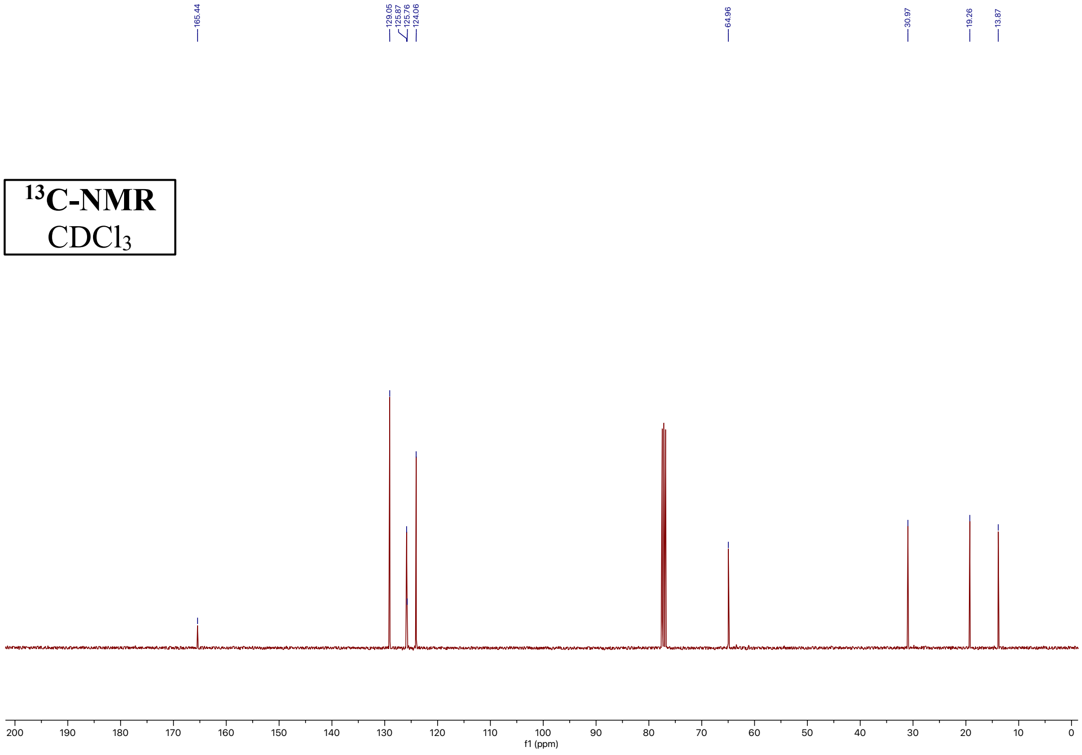

Supplement: Supplementary file 1 [file ja5c17554_si_001.pdf]
